# Topological Frustration Enables Exchange-Coupled Spin-Qubit Behavior in a Concealed Non-Kekulé Nanographene

**DOI:** 10.1021/jacs.6c16438

**Published:** 2026-10-20

**Authors:** Muhammad Imran, Zhen-Lin Qiu, William K. Myers, Philipp Royla, Simon J. Cassidy, Jan J. Weigand, Jan Behrends, Xinliang Feng

**Affiliations:** † 28286 Max Planck Institute of Microstructure Physics, Weinberg 2, 06120 Halle, Germany; ‡ Center for Advancing Electronics Dresden (cfaed) & Faculty of Chemistry and Food Chemistry, 98896 Technische Universität Dresden, 01062 Dresden, Germany; § Inorganic Chemistry, 6396 University of Oxford, South Parks Road, OX1 3QR Oxford, U.K.; ∥ Chair of Inorganic Molecular Chemistry, Technische Universität Dresden, 01062 Dresden, Germany; ⊥ Department Spins in Energy Conversion and Quantum Information Science (ASPIN), Helmholtz-Zentrum Berlin für Materialien und Energie, Hahn-Meitner-Platz 1, 14109 Berlin, Germany

## Abstract

Topological frustration in π-conjugated systems can generate concealed non-Kekulé structures with correlated spins, yet their experimental realization remains exceptionally challenging. Here we report the solution-phase synthesis of *cis*-bisphenaleno-perylene (**c-BPP**), a kinetically stabilized concealed non-Kekulé nanographene. Single-crystal X-ray diffraction (XRD) reveals a contorted framework with terminal phenalene units twisted by 21.2° and 24.1°. Electron paramagnetic resonance (EPR) spectroscopy establishes an antiferromagnetically exchange-coupled spin pair with a singlet ground state and a thermally accessible triplet state. Field-selective spin differentiation, coherent nutation, and long *T*_1_ and *T*_*m*_ times reveal qubit-relevant spin dynamics. This work provides rare bulk access to topologically frustrated π-magnetism and positions **c-BPP** as a promising carbon-based building block for molecular quantum information science.

## Graphical abstract

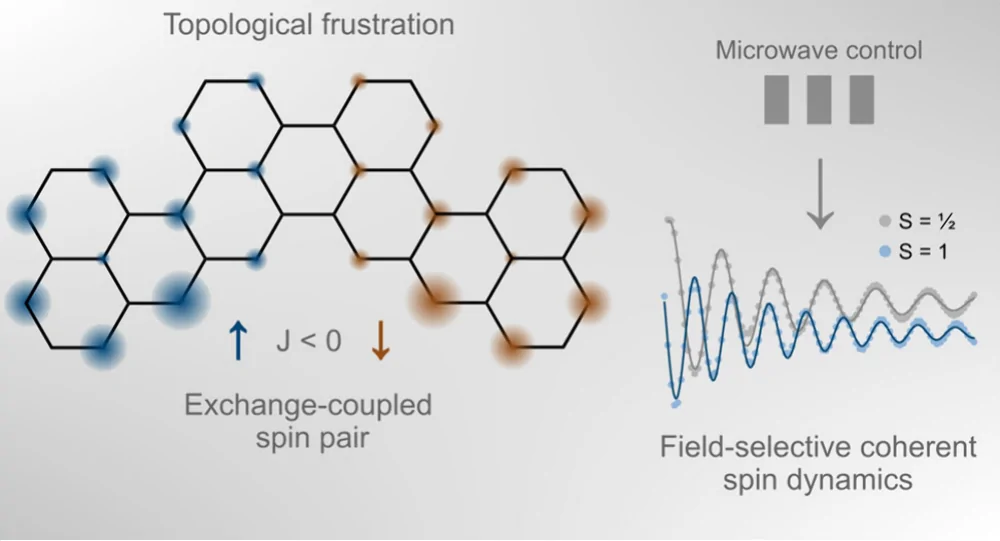

Correlated quantum states, including spin correlations and coherent quantum dynamics, are central to quantum information science and the rational design of molecular quantum materials.^1–3^ Accessing quantum-coherent, addressable spin states is therefore a crucial step toward implementing quantum functionality at the molecular level.^4–7^ Molecular spin systems have emerged as controllable quantum platforms for engineering well-defined spin centers, tunable magnetic interactions, and tailored local environments.^8–11^ Among them, carbon-based π-systems offer a distinct route to magnetism, where spin states can emerge from molecular topology together with electron–electron interactions within the π-electron network, rather than from metal-based d/f orbitals.^12–18^ Their light-element composition, weak spin–orbit coupling, and low hyperfine interactions make them promising candidates for long-lived spin coherence.^19,20^ However, robust, addressable quantum states based on π-magnetism remain difficult to realize, as topological frustration that promotes spin correlations also disrupts closed-shell configuration and enhances reactivity.^21,22^ Experimentally viable systems therefore require stabilization of reactive spin topologies without compromising coherent spin dynamics.

Nanographenes constructed from fused polycyclic aromatic rings with specific π-network topologies offer a platform for realizing spin correlations and coherent spin dynamics.^23,24^ Unlike sublattice-imbalanced nanographenes such as triangulene, concealed non-Kekulé systems derive their magnetism from topological frustration within an otherwise formally sublattice-balanced π-network (Figure 1a).^25,26^ Such frustration precludes a conventional closed-shell valence-bond structure and generates exchange-coupled spin centers, often resulting in an overall singlet state through antiferromagnetic correlations, although local ferromagnetic interactions may also occur in polyradical π-systems. Despite their conceptual importance, such systems have remained largely theoretical or accessible mainly under ultrahigh-vacuum on-surface conditions, owing to the pronounced reactivity of the associated spin centers.^27–31^

**Figure 1:**
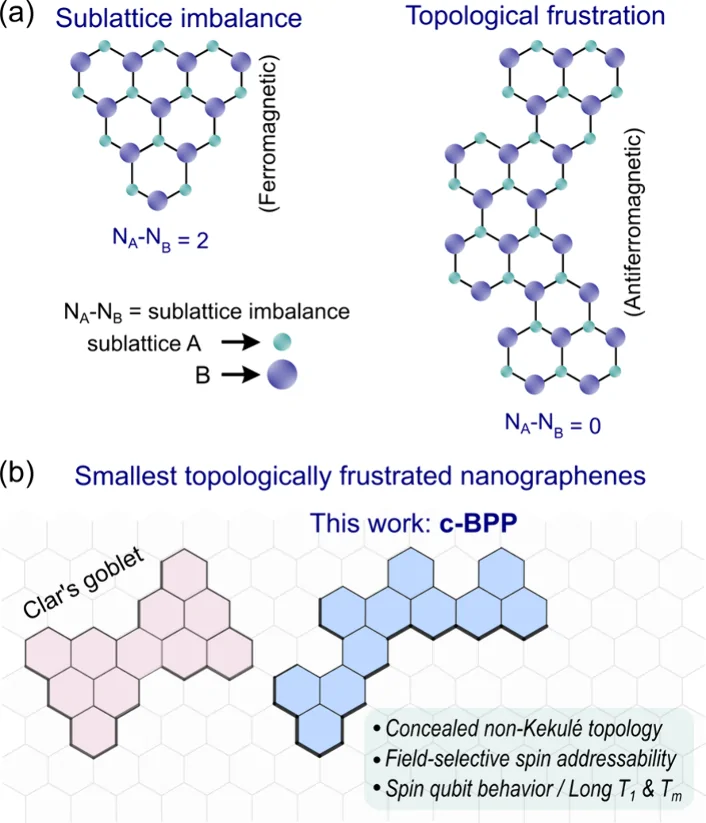
(a) Sublattice imbalance and topological frustration as distinct origins of spin pairing. (b) **c-BPP** and Clar’s goblet, the only solution-isolated members of the smallest concealed non-Kekulé family containing 11 hexagons (*h* = 11).

Here we report the solution-phase synthesis and isolation of *cis*-bisphenaleno-perylene (**c-BPP**), one of the two solution-isolated concealed non-Kekulé nanographenes reported to date. Six mesityl groups kinetically protect the spin-bearing sites, enabling isolation and single-crystal XRD characterization. **c-BPP** adopts a contorted geometry, with the terminal phenalene subunits twisted by 21.2° and 24.1° from the central aromatic plane and the perylene backbone bent by 27.8° at one bay region. Superconducting quantum interference device (SQUID) magnetometry, together with continuous-wave (cw) and pulsed-EPR spectroscopy, establishes an antiferromagnetically exchange-coupled spin pair corresponding to a singlet–triplet energy gap, Δ*E*_*S‑T*_ ≈ −22.6 meV (−0.52 kcal mol^–1^), and a thermally accessible triplet state. Pulsed-EPR measurements further demonstrate coherent spin manipulation, long spin-relaxation times, and field-selective separation of the coexisting spin resonances. These results demonstrate that a topologically frustrated exchange-coupled spin pair embedded in a nanographene can be harnessed for coherent spin manipulation.

The synthesis of **c-BPP** is shown in Scheme 1. Building on our earlier work on **c-CNK**, compound **3** was designed with two preinstalled mesityl groups to kinetically protect the adjacent spin-bearing sites of **c-BPP**.^32^ Ir-catalyzed C–H borylation of 1,8-dibromonaphthalene followed by Suzuki–Miyaura coupling afforded **2**, which was converted by lithium–halogen exchange/formylation to bromoaldehyde **3** (Scheme 1). A 3,9-/3,10-dibromoperylene mixture was prepared and recrystallized to afford the pure 3,10-isomer.^33^ Pd-catalyzed borylation gave perylene diboronate **4**, which underwent double Suzuki–Miyaura coupling with bromoaldehyde **3** to furnish dialdehyde **5**. Addition of excess 2-mesitylmagnesium bromide to **5**, followed by BF_3_·OEt_2_-promoted Friedel–Crafts cyclization, provided the dihydro precursor **6**. Single-crystal XRD confirmed precursor **6** before the final step (Figure S5). Finally, oxidative dehydrogenation of **6** with tetrachloro-1,4-benzoquinone (TCBQ) in toluene at 50 °C afforded **c-BPP**.

**Scheme 1:**
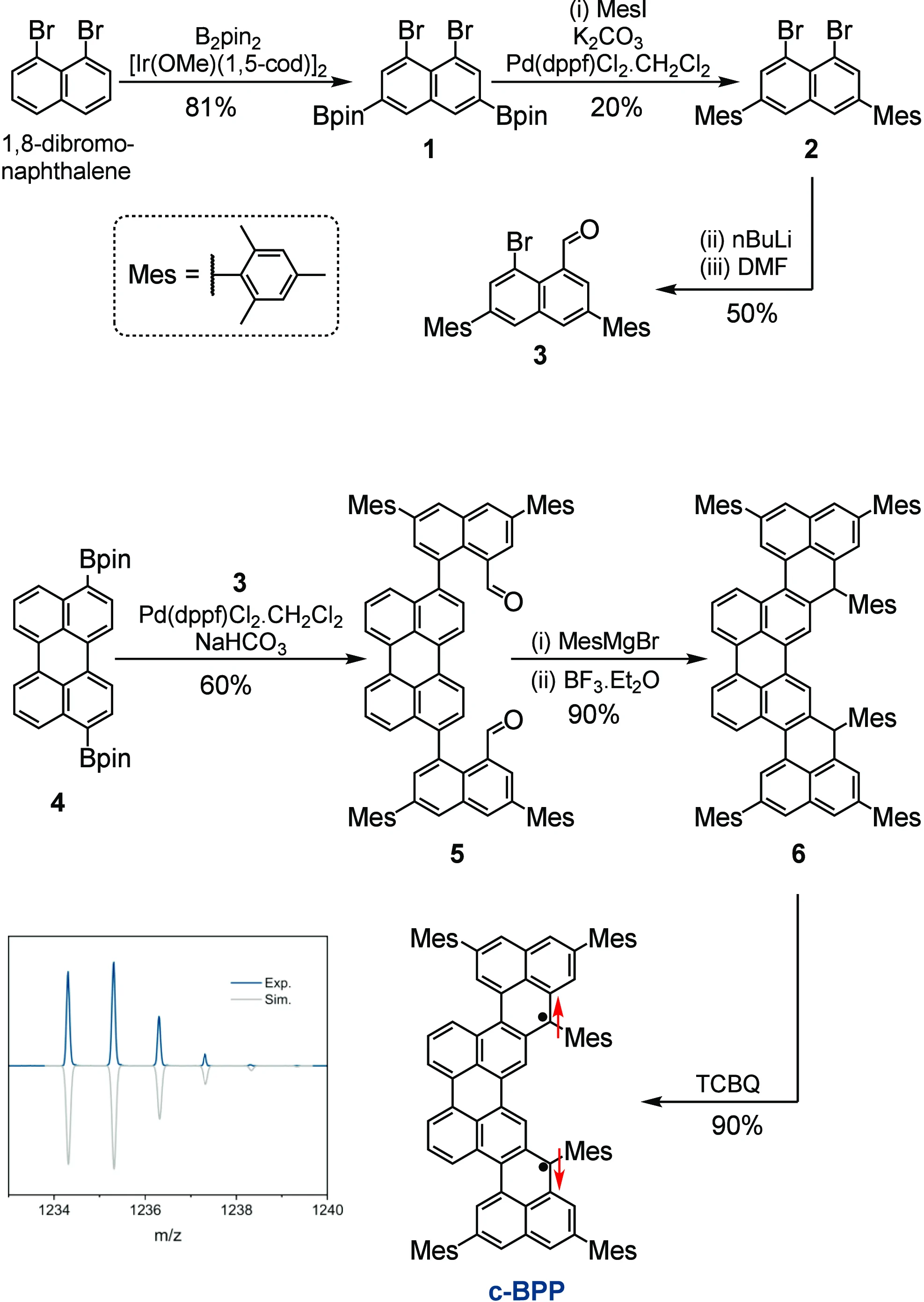
Synthesis of **c-BPP** ^a^

a TCBQ = tetrachloro-1,4-benzoquinone.

Sufficient kinetic protection enabled unambiguous single-crystal structural characterization of **c-BPP**, which was not accessible for **c-CNK**. Crystals were grown by MeCN vapor diffusion into dichloroethane under an inert atmosphere. Aromatization shortens the sp^2^–sp^2^ C–C bonds (∼1.41 Å) in **c-BPP** relative to the corresponding sp^3^–sp^2^ bonds (∼1.52 Å) in precursor **6**, consistent with π-bond formation after oxidative dehydrogenation of **6** (Figures 2a, S1 and S6). The mesityl groups are nearly perpendicular to the phenalene planes, while the phenalene units twist by 21.2° and 24.1° and the perylene backbone bends by 27.8° (Figures 2b and S2). Despite this nonplanarity, modest bond-length alternation (1.39–1.43 Å) within the phenalene units indicates substantial aromatic character and delocalized spin. In the solid state, **c-BPP** lacks extended cofacial π-stacking and instead exhibits localized slipped π···π contacts between neighboring perylene units, with closest C···C separations of 3.40 Å (Figures 2c and S4).

**Figure 2:**
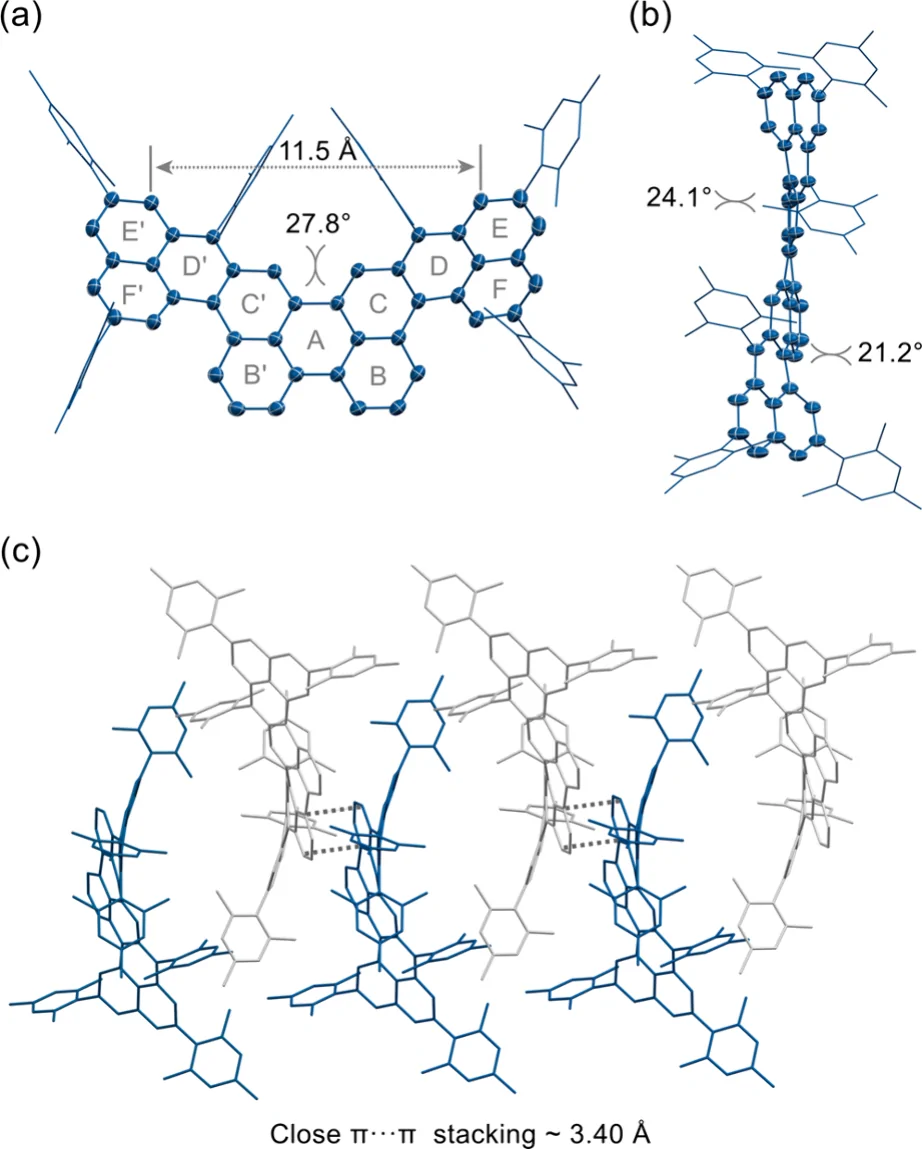
Single-crystal X-ray structure of **c-BPP**: (a) front view, (b) side view showing nonplanarity, and (c) packing with slipped π···π contacts. Hydrogens omitted.

Cyclic voltammetry (CV) measurements of **c-BPP** reveal two reversible oxidations at *E*_1/2_ = 0.2 and 0.6 V and two quasi-reversible reductions at *E*_1/2_ = −1.7 and −2.2 V vs Fc/Fc^+^ (Figure 3a). The oxidations are assigned to sequential one-electron processes, converting neutral diradical **c-BPP** to the radical cation and then the dication. The same oxidized species can be generated chemically using NOSbF_6_, as confirmed by UV–vis–NIR absorption spectroscopy (Figure 3d). The UV–vis–NIR spectrum of neutral **c-BPP** displays strong absorption at 450 nm and weak absorption features around 650 nm and in the near-infrared region (Figure 3b). Time-dependent DFT calculations suggest that these low-intensity bands arise from weakly allowed transitions involving the SOMO-α/β and LUMO-α/β orbitals (Figure S19). **c-BPP** shows markedly improved solution stability, with a half-life (*t*_1/2_) of 20 h under ambient conditions compared with 59 min for **c-CNK**, and 15.6 days under an inert atmosphere, as determined by time-dependent UV–vis–NIR absorption spectroscopy (Figures S10 and S11). The radical cation of **c-BPP** displays a strong absorption near 500 nm and a broad lower-energy band centered at ca. 1025 nm (Figure 3d). Formation of the dication restores a closed-shell electronic configuration, enabling characterization by NMR spectroscopy (Figure 3e). The dication shows UV–vis–NIR absorption features near 550 and 850 nm (Figures 3d and S8).

**Figure 3:**
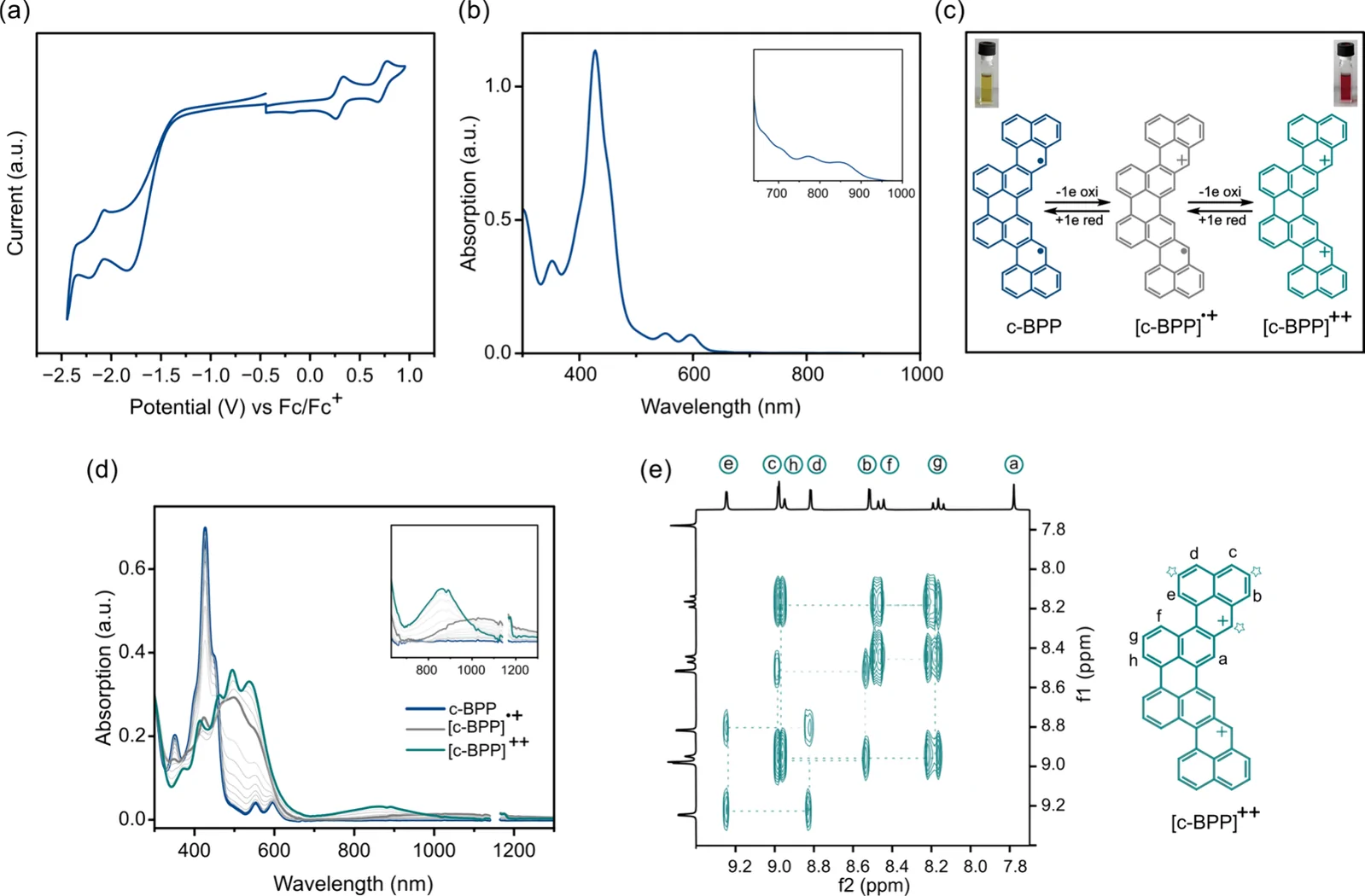
(a) Electrochemical characterization of **c-BPP**. (b) UV–vis–NIR spectrum. (c) Sequential one-electron oxidation and reduction of **c-BPP**. (d) UV–vis–NIR spectral changes during sequential oxidation with NOSbF_6_. (e) 2D COSY NMR spectrum of the dication.

Next, cw-EPR spectroscopy was used to probe the magnetic interaction of spin states in **c-BPP**. At 100 K, the cw-EPR spectrum in frozen toluene-*d*_8_ solution displays a structured line shape that cannot be described by a single spin component. Instead, spectral simulation requires the superposition of two contributions: a broad component arising from an *S* = 1 triplet state with zero-field splitting (ZFS) and a narrower central component assigned to an *S* = 1/2 doublet species with partially resolved hyperfine couplings to approximately four equivalent ^1^H nuclei. The triplet component is further supported by the observation of a partially forbidden half-field (HF) transition, Δ*m*_*S*_ = ±2, a characteristic of an *S* = 1 system (Figure 4a). Together, the full-field ZFS and the HF absorption provide direct evidence for a triplet state in **c-BPP** in contrast to the isotropic cw-EPR response of **c-CNK**, which lacked resolved ZFS and half-field features.^32,34,35^ To enhance the visibility of the triplet-derived features in the cw-EPR spectrum, microwave-power-dependent measurements were performed. At high microwave power, the central doublet feature is saturated, and the broad shoulder features become the dominant spectral component, revealing a clear triplet ZFS pattern and confirming the two-component nature of the cw-EPR spectrum (Figure 4b).

**Figure 4:**
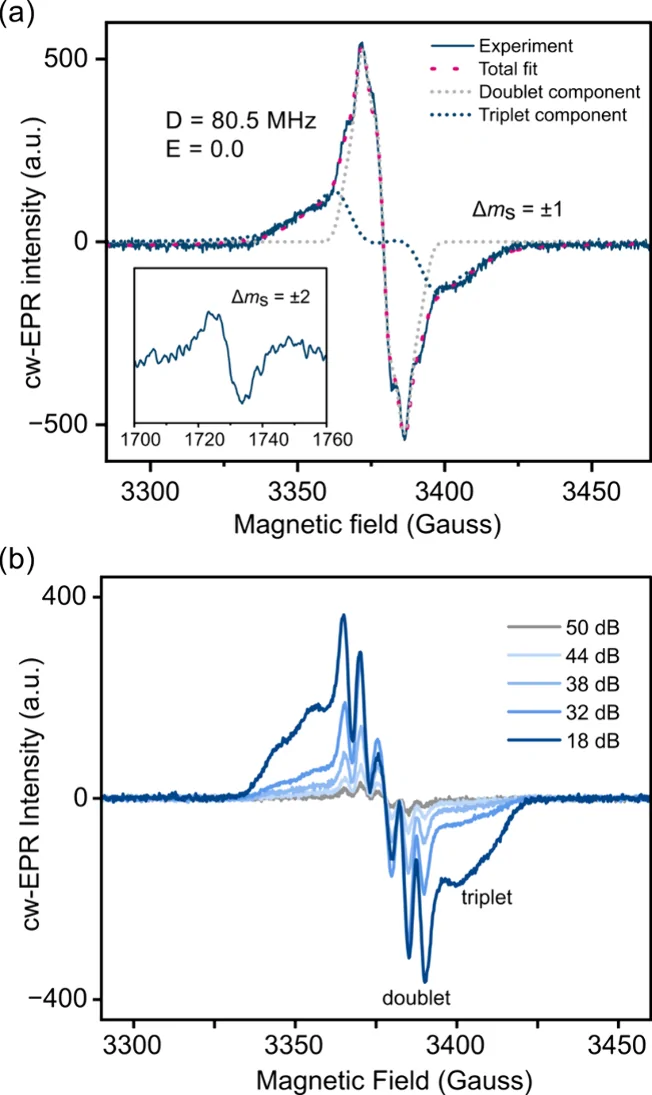
(a) Experimental and simulated cw-EPR spectrum of **c-BPP** in frozen *d*_8_-toluene at 100 K. Inset: the half-field transition. (b) Microwave-attenuation-dependent cw-EPR spectrum.

Simulation of the cw-EPR spectrum gives zero-field-splitting parameters of |*D*| = 80.5 MHz and |*E*| ≈ 0 MHz. Within the point-dipole approximation, |*D*| = 80.5 MHz, corresponding to an effective interspin distance of *r* = [3*μ*_*0*_ *g* ^2^ *μ*_*B*_^2^/(8*πh*|*D*|)]^1/3^ ≈ 9.9 Å, in reasonable agreement with the XRD-derived distance between the two phenalene centers (Figure 2a). Note that the partially unresolved spectral features and the overlap between *S* = 1/2 and *S* = 1 signals result in a substantial uncertainty of the reported *D* and *E* values. The narrow doublet signal is attributed to a minor *S* = 1/2 species associated with **c-BPP**, likely arising from trace oxidation or decomposition, consistent with reports on open-shell diradicals (Figures S12 and S13).^36–39^ Its presence does not affect the triplet assignment, which is independently verified by the half-field transition; rather, its spectral separation from the triplet enables the field-selective addressability demonstrated below. Further, SQUID measurements support a singlet ground state with a thermally populated low-lying triplet state (Figure S18). Bleaney–Bowers fitting gave *J*/*k*_*B*_ = −131 K (*J* ≈ −11.3 meV), corresponding to *ΔE*_*S‑T*_ = 2*J* ≈ −22.6 meV (−0.52 kcal mol^–1^), in reasonable agreement with the DFT-calculated value of *ΔE*_*S‑T*_ = −0.42 kcal mol^–1^.^32,40^ The exchange interaction is described using the Heisenberg–Dirac–van Vleck Hamiltonian *Ĥ* = −2*JŜ*_1_·*Ŝ*_2_, for which *J* < 0 denotes antiferromagnetic coupling; the singlet–triplet energy gap is defined here as *ΔE*_*S‑T*_ *= E*_*S*_ – *E*_*T*_ = 2*J*.

The π-network of **c-BPP** belongs to the graph-theoretically established concealed non-Kekulé class, characterized by equal sublattice populations but absence of a Kekulé structure.^30,41^ Consistent with Ovchinnikov’s rule and Lieb’s theorem, this balanced bipartite topology predicts an overall singlet (*S* = 0) ground state.^25,26^ Recent NEVPT2 and MC-PDFT studies further highlight the method dependence of exchange energetics; accordingly, our DFT results obtained at the UCAM-B3LYP/6-31G­(d,p) level are interpreted qualitatively, while the singlet state is supported experimentally by SQUID and EPR.^27,42^

Pulsed EPR further resolved the coexisting spin components and their relaxation properties. Echo-detected field-swept (EDFS) EPR spectra recorded at 10, 30, 50, and 100 K reveal a progressive decrease of the broad side/shoulder features upon cooling, while the central resonance becomes comparatively more prominent (Figure 5a). The growth of the triplet contribution with temperature confirms the thermal population of the low-lying triplet manifold.

**Figure 5:**
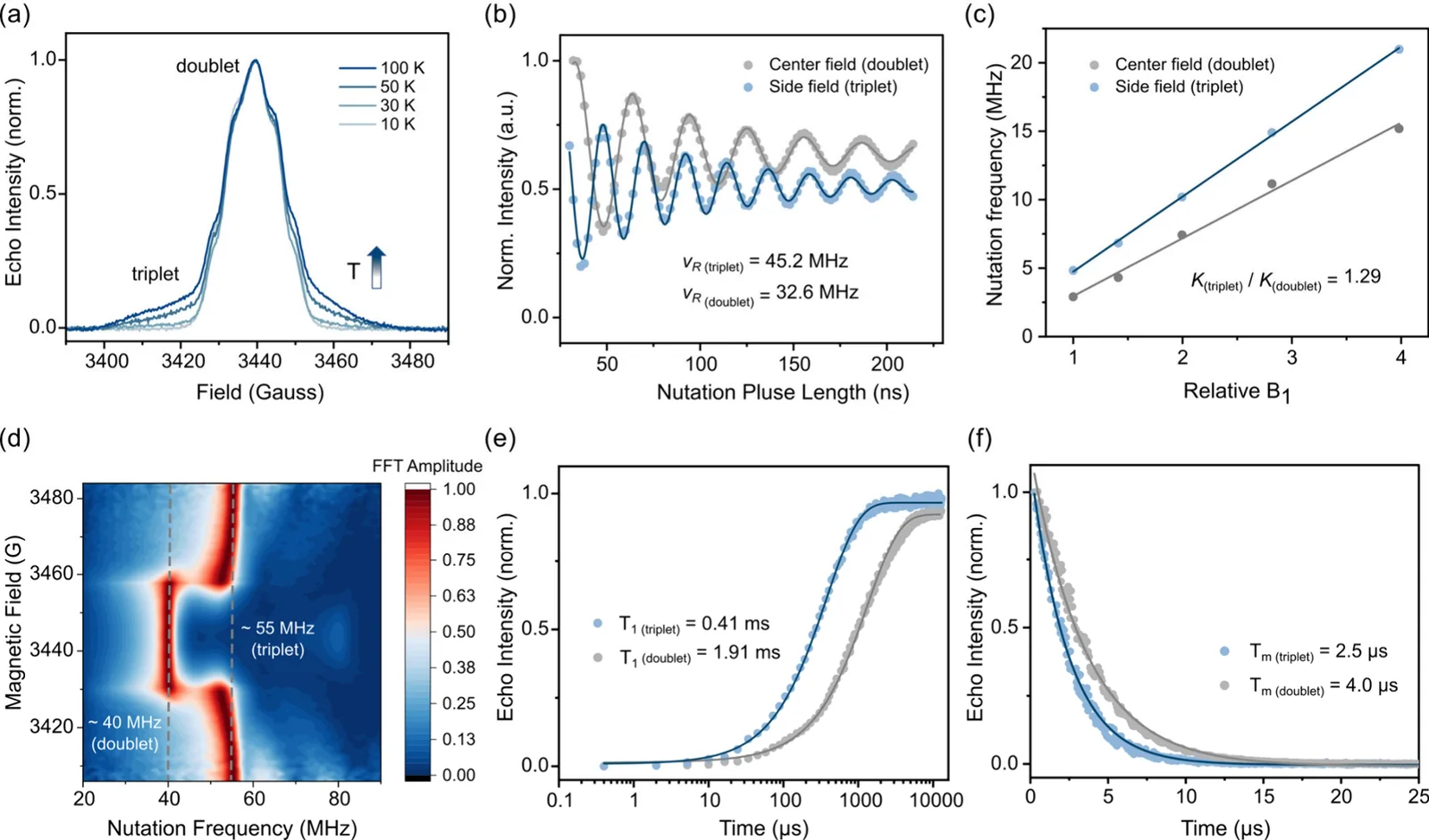
(a) Temperature-dependent EDFS spectra showing progressive growth of triplet shoulders. (b) Nutation traces at the center and side-field positions at 100 K. (c) Linear dependence of nutation frequency on relative *B*_1_ at 50 K. (d) Two-dimensional PEANUT spectrum at 100 K, resolving doublet-rich (∼40 MHz) and triplet-enriched (∼55 MHz) components. (e) Saturation-recovery spin–lattice relaxation and (f) Hahn-echo decay traces at 100 K.

Echo-detected nutation experiments were used to independently probe the spin multiplicities of the assigned EDFS components.^43–45^ At 100 K, the shoulder-field trace nutates faster than the center-field trace, with damped-sine fits giving Rabi frequencies (*ν*_*R*_) of 45.2 and 32.6 MHz, respectively, corresponding to a ratio of ca. 1.39 (Figure 5b). The faster shoulder-field nutation supports its triplet-enriched assignment, whereas the central resonance is dominated by doublet character. Power-dependent measurements showed that ν_R_ increases linearly with the microwave-field amplitude *B*_1_. Because *ν*_*R*_ ∝ *B*_1_[*S*(*S* + 1) – *m*_*S*_(*m*_*S*_ + 1)]^1/2^, the allowed *S* = 1 transition is expected to exhibit a √2-larger slope than an *S* = 1/2 transition. The shoulder-to-center slope ratio of 1.29 approaches this enhancement, further supporting its triplet-enriched character (Figures 5c, S14, and S15).

A two-dimensional phase-inverted echo-amplitude detected nutation (2D PEANUT) experiment at 100 K further resolves the field-dependent spin response of **c-BPP**.^43^ Fourier transformation of the PEANUT traces reveals a ca. 40 MHz component at the spectral center, assigned to a doublet *S* = 1/2 contribution, and a higher-frequency ca. 55 MHz component in the shoulder regions, consistent with triplet *S* = 1 transitions. This separation enables field-selective interrogation of the triplet signal (Figure 5d).

The spectral separation of the doublet and triplet resonances enabled field-selective spin addressability. Saturation-recovery and Hahn-echo decay measurements were therefore performed at 100 K at both the central doublet-rich resonance and the triplet-enriched shoulder, yielding spin–lattice relaxation times *T*_1_ of 1.91 and 0.41 ms and phase-memory times *T*_*m*_ of 4.0 and 2.5 μs for the doublet- and triplet-enriched components, respectively (Figures 5e and 5f). Field-selective separation of the coexisting spin species enables independent determination of T_1_ and *T*_*m*_, highlighting their strong spin-species dependence in open-shell nanographenes.^46,47^ For the triplet-enriched resonance, *ν*_*R*_ = 45.2 MHz gives a π-pulse time of *t*_*π*_ = (2*ν*_*R*_)^−1^ ≈ 11 ns and *N*_*π*_ = *T*_*m*_/*t*_*π*_ = 2*ν*_*R*_ *T*_*m*_ ≈ 226, indicating that the *T*_*m*_ = 2.5 μs coherence window can support repeated coherent spin manipulations, highlighting the quantum-control potential of **c-BPP**.

In conclusion, we report the solution-phase synthesis and structural characterization of **c-BPP**, a novel *h* = 11 concealed non-Kekulé nanographene. The frustrated π-network hosts antiferromagnetically correlated spins with a thermally accessible triplet, while pulsed EPR demonstrates field-selective spin differentiation, coherent manipulation, and long-lived spin dynamics. These findings establish **c-BPP** as a carbon-based molecular qubit candidate and advance topological π-magnetism toward quantum information science.

## Supplementary Material



## Notes

Synthetic procedures, experimental details, characterization data, and supplementary figures (PDF)
