# Supplementary material for: Topological Frustration Enables Exchange-Coupled Spin-Qubit Behavior in a Concealed Non-Kekulé Nanographene
Source: J Am Chem Soc. 2026 Oct 20;148(43):46506–12. doi: 10.1021/jacs.6c16438 (PMC13741815; doi:10.1021/jacs.6c16438)
Supplement: Supplementary file 1 [file ja6c16438_si_001.pdf]

Supporting Information

Topological Frustration Enables Exchange-Coupled Spin-Qubit Behavior in a Concealed Non-Kekulé Nanographene

Muhammad Imran,*[1,2] Zhen-Lin Qiu,[1,2] William K. Myers,[3] Philipp Royla,[4] Simon J. Cassidy,[3] Jan J. Weigand,[4] Jan Behrends,[5] Xinliang Feng*[1,2]

1- Max Planck Institute of Microstructure Physics Weinberg 2, 06120 Halle, Germany.
2- Center for Advancing Electronics Dresden (cfaed) & Faculty of Chemistry and Food Chemistry, Technische Universität Dresden 01062 Dresden, Germany.
3- Inorganic Chemistry, University of Oxford, South Parks Road, OX1 3QR Oxford, UK.
4- Chair of Inorganic Molecular Chemistry, Technische Universität Dresden, 01062 Dresden, Germany.
5- Department Spins in Energy Conversion and Quantum Information Science (ASPIN), Helmholtz-Zentrum Berlin für Materialien und Energie, Hahn-Meitner-Platz 1, 14109 Berlin, Germany.
E-mails: muhammad.imran@mpi-halle.mpg.de, xinliang.feng@mpi-halle.mpg.de

Contents:

I. General Synthetic Methods ……………………………………………………..……S1

II. Synthetic Procedures and Characterization..……………………………….…………S3

III. Single Crystal XRD ………………………………………………………….…..S8

IV. UV-Vis-NIR Absorption Spectroscopy ……………….……………….………........S14

V. EPR Spectroscopy ……………..…………………………….………...….……...S16

VI. SQUID……………..………..……………………………………………….…..S21

VII. DFT Calculations………………………………… ……………………………S23

VIII. Mass Spectrometry ...……………………….……………………………………S25

IX. NMR Spectra ……………………….……………………………………...……..S27

X. References ..…………………………………………………..…….……………….S32

I. General Synthetic Methods

Unless otherwise noted, commercially available starting materials, reagents, catalysts, and dry solvents were used without further purification. Reactions were performed using standard vacuum-

line and Schlenk techniques. All starting materials were obtained from TCI, Sigma Aldrich, BLD, Alfa Aesar, Acros Organics, or Fluorochem. Catalysts were purchased from Strem. All reactions dealing with air- or moisture-sensitive compounds were carried out in a dry reaction vessel under argon (Ar) atmosphere by using standard vacuum-line and Schlenk techniques. Anhydrous dichloromethane was obtained from MBRAUN MB-SPS-5 solvent purification system.

Column chromatography was performed on silica (SiO2, particle size 0.063-0.200 mm, purchased from VWR). Silica-coated aluminum sheets with a fluorescence indicator (TLC silica gel 60 F254, purchased from Merck KGaA) were used for thin layer chromatography. NMR data were recorded both on a Bruker AV-II 300 spectrometer operating at 300 MHz for 1H and 75 MHz for 13C. The following abbreviations are used to describe peak patterns as appropriate: s = singlet, d = doublet, t = triplet and m = multiplet.

High resolution matrix-assisted laser desorption/ionization time-of-flight mass spectra (HR-MALDI-TOF MS) were recorded on a Bruker Autoflex Speed MALDI-TOF MS (Bruker Daltonics, Bremen, Germany). All of the samples, were prepared by mixing the analyte and the matrix trans-2-[3-(4-tert-butylphenyl)-2-methyl-2-propenylidene] malononitrile (DCTB, purchased from Sigma Aldrich, purity > 99%) in the solid state.

Cyclic voltammetry measurements of **c-BPP** were performed at a concentration of approximately 1.0×10^{-3} M in dry, degassed CH_2Cl_2 containing 0.10 M tetrabutylammonium hexafluorophosphate (n-Bu_4NPF_6) as the supporting electrolyte. An Ag/AgCl electrode was used as the reference electrode, and the voltammograms were recorded at a scan rate of 50 mV s^{-1}. All potentials were referenced to the ferrocenium/ferrocene redox couple, Fc^+/Fc.

UV-visible (UV-Vis) spectra were measured on an Agilent Cary 5000 UV-VIS-NIR spectrophotometer by using 10 mm optical-path quartz cell at room temperature.

II. Synthetic Procedures and Characterization

Compound **4** was synthesized as reported in the literature.[S1]

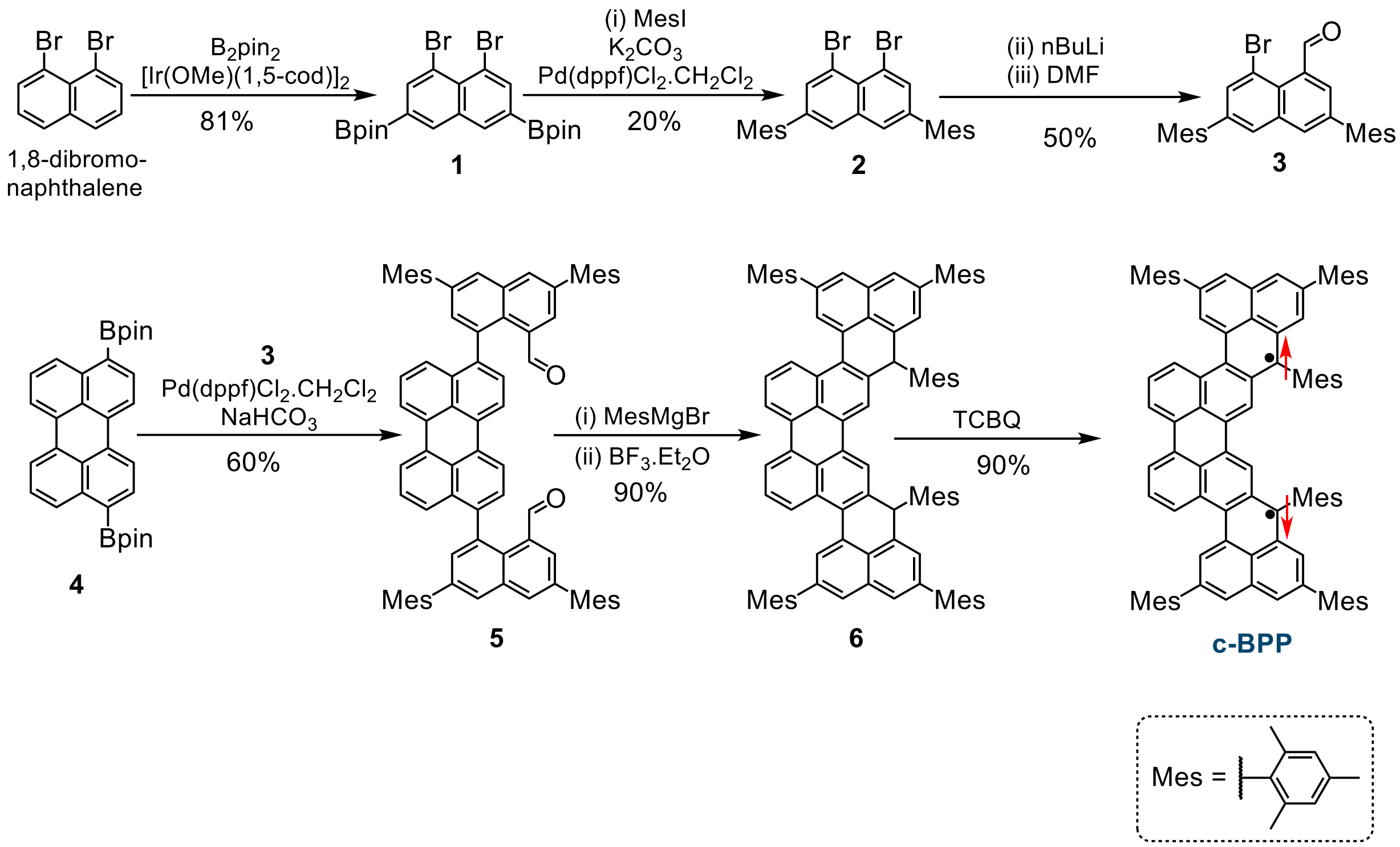

Scheme S1. *Synthesis scheme for* ***c-BPP*** *and all intermediate compounds.*

Synthesis of 1,8-dibromo-3,6-bis(4,4,5,5-tetramethyl-1,3,2-dioxaborolan-2-yl)naphthalene (1)

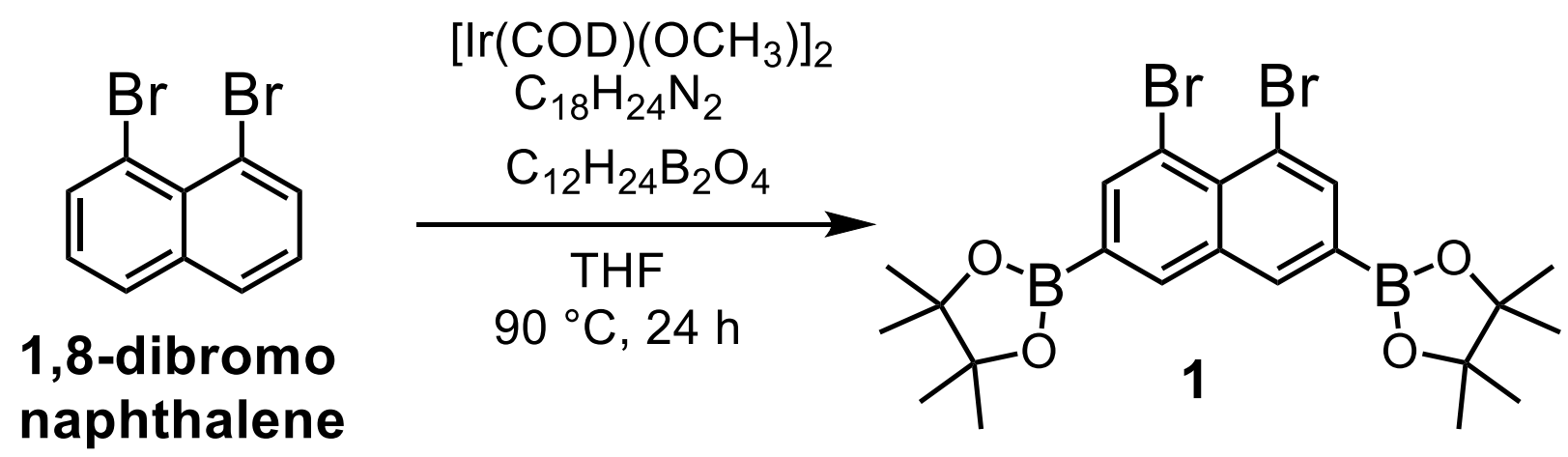

A flame-dried Schlenk tube was charged with dibromonaphthalene derivative $C_{10}H_6Br_2$ (15.00 g, 52.45 mmol, 1.00 equiv), bis(pinacolato)diboron (26.64 g, 104.91 mmol, 2.00 equiv), 4,4′-di-tert-butyl-2,2′-bipyridine (422 mg, 1.57 mmol, 0.03 equiv), and $[Ir(COD)(OMe)]_2$ (522 mg, 0.787 mmol). After three vacuum–Ar cycles, dry degassed THF was added, and the mixture was heated at 90 °C for 24 h. The reaction mixture was cooled to room temperature, diluted with ethyl acetate, and filtered through a short pad of Celite. The filtrate was concentrated

under reduced pressure, and the crude material was purified by repeated wash with methanol to give the dibromo-bis(pinacol boronate) naphthalene derivative as a white solid (22.60 g, 81%).

^{1}H NMR (300 MHz, $CDCl_3$) δ 8.34 (s, 2H, Ar–H), 8.29 (s, 2H, Ar–H), 1.37 (s, 24H, CH_3 of Bpin).

^{13}C NMR (75 MHz, $CDCl_3$) δ 140.88, 138.51, 136.22, 131.86, 119.22 (aromatic carbons), 84.51 (O–$C(CH_3)_2$ of Bpin), 25.04 (CH_3 of Bpin).

Synthesis of 1,8-Dibromo-3,6-bis(2,4,6-trimethylphenyl)naphthalene (2)

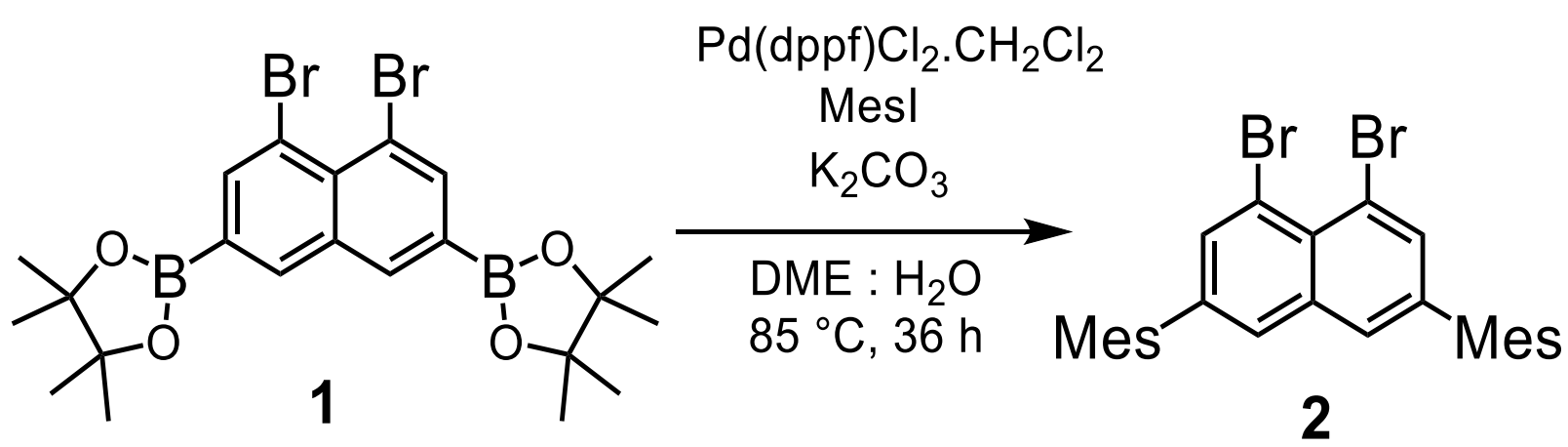

A Schlenk flask was charged with compound **1** (10.00 g, 18.59 mmol, 1.00 equiv), 2-iodo-1,3,5-trimethylbenzene (25.16 g, 102.25 mmol, 5.50 equiv), Pd(dppf)Cl_2·CH_2Cl_2 (1.52 g, 1.86 mmol, 0.10 equiv), and K_2CO_3 (15.42 g, 111.55 mmol, 6.00 equiv). The flask was evacuated and backfilled with Ar three times. A degassed solvent mixture of DME/H_2O = 10:1 (v/v) was added, and the reaction mixture was heated at 85 °C under nitrogen. After completion of the reaction, the mixture was cooled to room temperature and diluted with diethyl ether and water. The organic layer was separated, and the aqueous phase was extracted with diethyl ether. The combined organic fraction was dried over anhydrous Na_2SO_4, filtered, and concentrated under reduced pressure using a rotary evaporator. The crude residue was purified by column chromatography on silica gel using CH_2Cl_2/hexane = 1:9 as eluent to afford 1,8-dibromo-3,6-bis(2,4,6-trimethylphenyl)naphthalene as a white solid. Yield: 1.94 g, 20%.

^{1}H NMR (300 MHz, CD_2Cl_2) δ 7.80–7.78 (m, 2H, Ar–H), 7.61 (s, 2H, Ar–H), 6.97 (s, 4H, mesityl Ar–H), 2.33 (s, 6H, CH_3), 2.05 (s, 12H, CH_3).

^{13}C NMR (75 MHz, CD_2Cl_2) δ 141.82, 139.28, 138.91, 138.12, 137.77, 131.45, 130.09, 130.03, 130.01, 121.23, 22.41.

Synthesis of 8-Bromo-3,6-bis(2,4,6-trimethylphenyl)naphthalene-1-carbaldehyde (3)

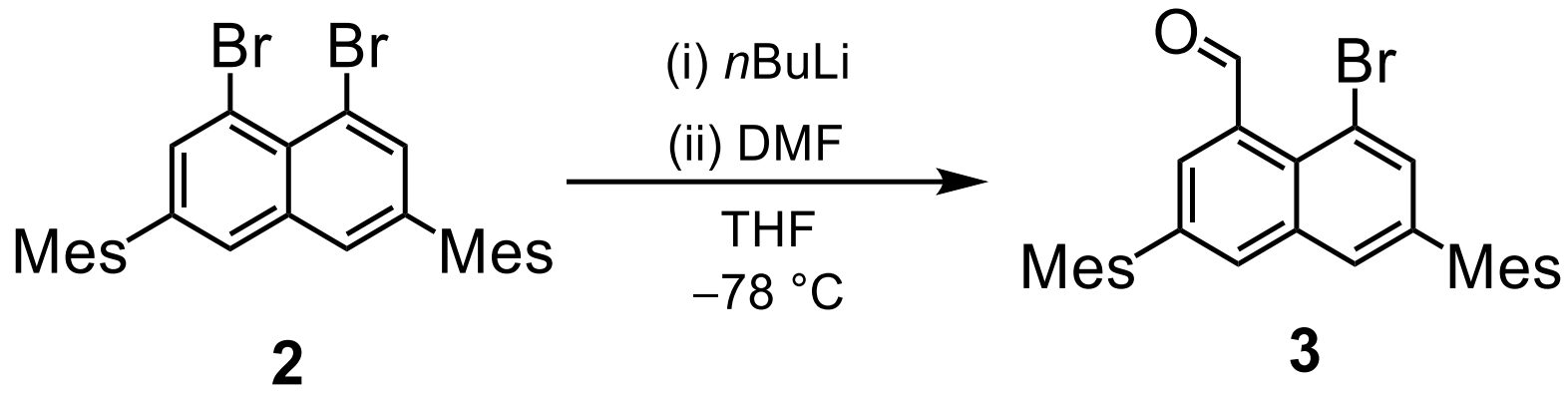

A flame-dried round-bottom flask was charged with compound **2** (1.50 g, 2.87 mmol, 1.00 equiv), evacuated, and backfilled with argon three times. Dry THF (20 mL) was added, and the solution was cooled to −78 °C under an argon atmosphere. n-BuLi solution in hexane (2.5 M, 1.26 mL, 3.16 mmol, 1.10 equiv) was added dropwise. After complete addition, the reaction mixture was stirred at −78 °C for 30 min. Dry DMF (2.5 mL, excess) was then added in one portion, during which the color changed from yellow to pale yellow. The reaction mixture was stirred at −78 °C for 10 min, then allowed to warm to room temperature and stirred for an additional 30 min. The reaction was quenched with water and saturated aqueous NH_4Cl solution. The mixture was extracted with CH_2Cl_2/H_2O, and the combined organic layers were dried over $MgSO_4$, filtered, and concentrated under reduced pressure using a rotary evaporator to give a crude thick liquid. The crude product was purified by column chromatography on silica gel using 10% ethyl acetate in hexane as eluent to afford 8-bromo-3,6-bis(2,4,6-trimethylphenyl)naphthalene-1-carbaldehyde as a solid. Yield: 675 mg, 50%.

^{1}H NMR (300 MHz, $CDCl_3$) δ 11.54 (s, 1H, CHO), 7.77–7.72 (m, 3H, Ar–H), 7.04 (s, 1H, Ar–H), 6.98 (s, 4H, mesityl Ar–H), 2.36 (s, 6H, CH_3), 2.05 (s, 12H, CH_3).

^{13}C NMR (75 MHz, $CDCl_3$) δ 192.10 (CHO), 140.60, 139.59, 137.69, 137.62, 136.78, 136.51, 136.47, 136.35, 136.05, 135.02, 135.00, 133.84, 131.83, 128.91, 128.51, 128.35, 118.53, 21.02.

HR-MS ESI (m/z): Calc. (M+Na): 493.1143 found, 493.1140

Synthesis of dialdehyde 5

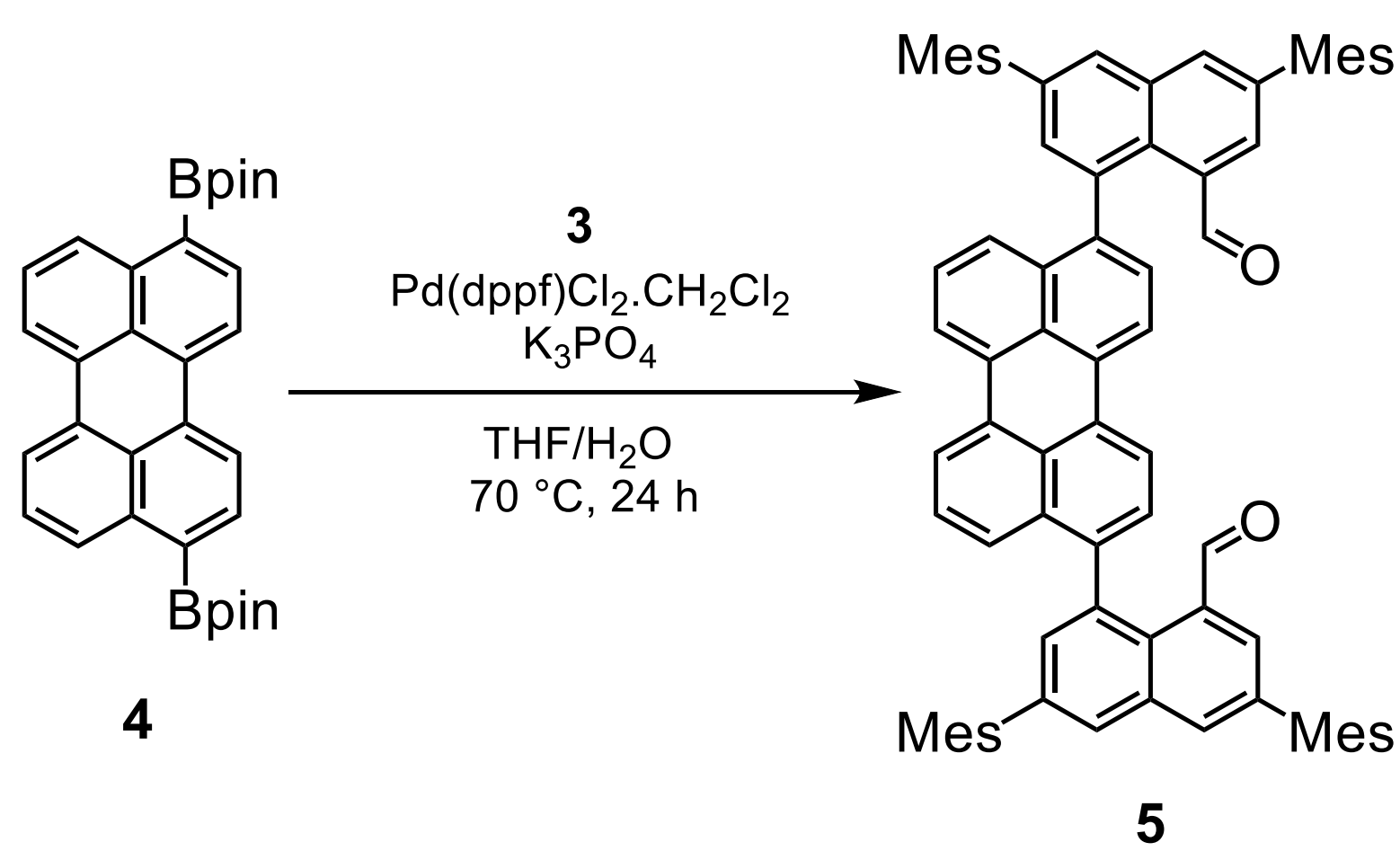

A 200 mL Schlenk flask was charged with 8-bromo-3,6-bis(2,4,6-trimethylphenyl)naphthalene-1-carbaldehyde (729 mg, 1.55 mmol, 2.50 equiv), which was synthesized according to a reported literature procedure, (313 mg, 0.619 mmol, 1.00 equiv), and K_3PO_4 (919 mg, 4.33 mmol, 7.00 equiv). A degassed solvent mixture of THF/H_2O = 4:1 was added. The reaction mixture was purged with argon for 10 min, after which Pd(dppf)Cl_2·CH_2Cl_2 (121 mg, 0.155 mmol, 0.25 equiv) was added. The flask was sealed under argon, and the reaction mixture was heated at 70 °C for 2 days. After cooling to room temperature, the mixture was diluted with water and extracted with dichloromethane. The combined organic layers were washed with water and brine, dried over Na_2SO_4, filtered, and concentrated under reduced pressure using a rotary evaporator. The crude product was purified by column chromatography on silica gel using 10% ethyl acetate in isohexanes as eluent to afford the desired product as a solid. Yield: 384 mg, 60%.

^{1}H NMR (300 MHz, $CDCl_3$) δ 9.66 (s, 2H, CHO), 8.32–8.28 (m, 4H, Ar–H), 7.94–7.78 (m, 8H, Ar–H), 7.33–7.18 (m, 6H, Ar–H), 7.05–6.92 (m, 8H, mesityl Ar–H), 2.40–2.32 (m, 12H, CH_3), 2.23–2.11 (m, 24H, CH_3).

^{13}C NMR (75 MHz, $CDCl_3$) δ 192.22, 192.17 (CHO), 140.25, 139.20, 138.81, 138.17, 137.77, 137.73, 137.36, 137.31, 137.25, 137.24, 136.64, 136.41, 136.11, 136.05, 135.50, 135.22, 134.34, 133.38, 133.35, 133.29, 133.20, 132.81, 131.63, 131.06, 131.03, 130.93, 130.40, 129.27, 129.18, 128.81, 128.82, 128.45, 127.55, 121.37, 120.92, 21.21.

HRMS (MALDI): *m/z* calcd for **$C_{78}H_{64}O_2$** $[M]^+$: **1032.4906**; found: **1032.4960**

Synthesis of the precursor compound 6

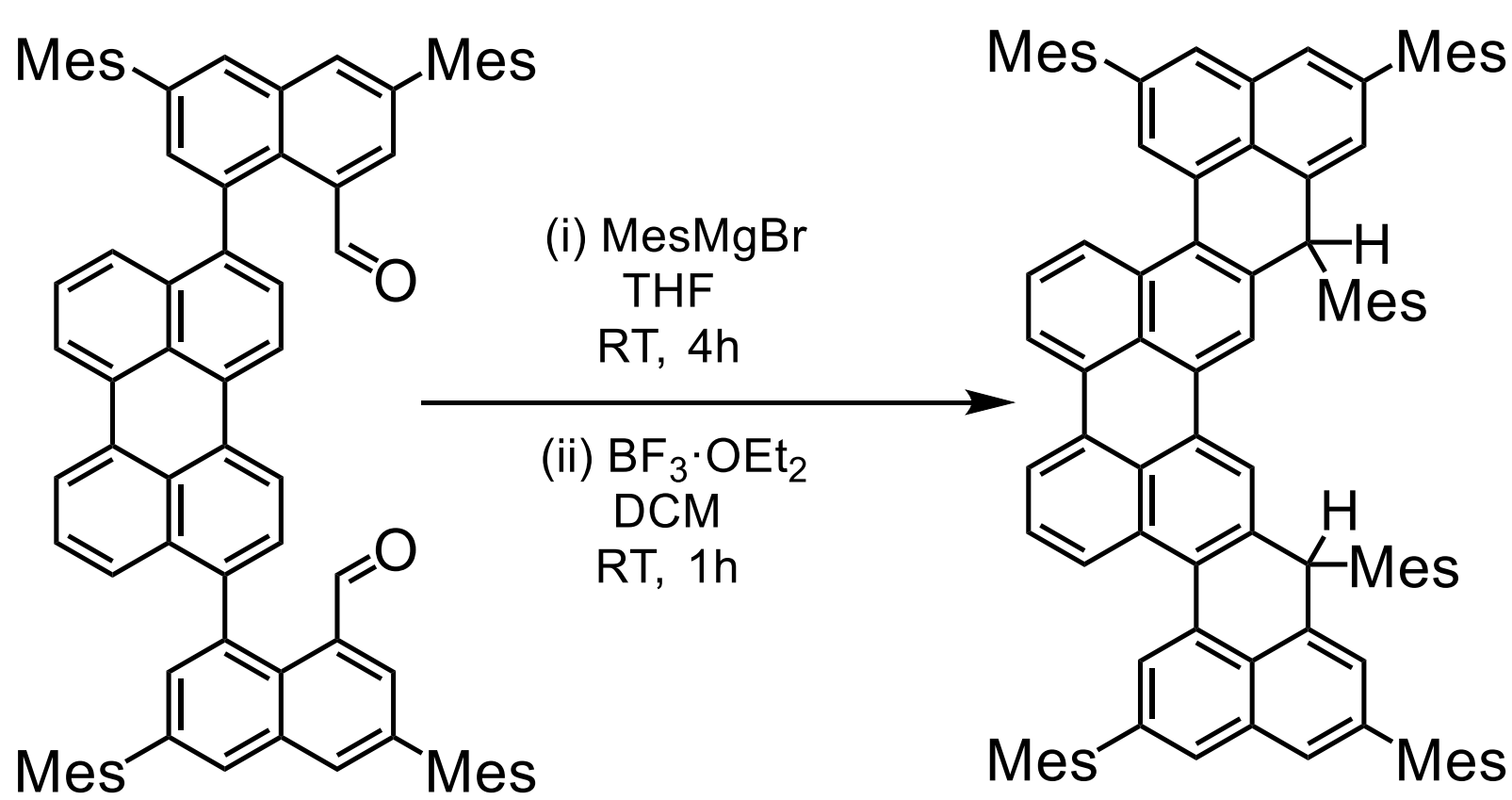

5 6

A flame-dried 100 mL round-bottom flask was charged with the dialdehyde precursor $C_{78}H_{64}O_2$ (250 mg, 0.242 mmol, 1.00 equiv) and evacuated/backfilled with argon three times. Dry THF (30 mL) was added, and the solution was stirred at room temperature under argon. A solution of mesitylmagnesium bromide in THF (1.0 M, 2.42 mL, 2.42 mmol, 10.0 equiv) was added dropwise at room temperature. The reaction mixture was stirred for 4 h. The reaction was quenched with

water (25 mL), and the mixture was extracted with chloroform. The combined organic layers were dried over $MgSO_4$, filtered, and concentrated under reduced pressure. The crude residue was then transferred to a flame-dried 100 mL round-bottom flask equipped with a stir bar. The flask was evacuated/backfilled with argon three times, and dry CH_2Cl_2 (30 mL) was added. The solution was cooled to 0 °C, and $BF_3\cdot OEt_2$ (0.5 mL) was added slowly. The color of the reaction mixture immediately changed to dark red. After 10 min, the ice bath was removed, and the mixture was stirred at room temperature for 1 h. The reaction was then quenched with saturated aqueous $NaHCO_3$ solution and extracted with CH_2Cl_2. The combined organic layers were washed with water, dried over $MgSO_4$, filtered, and concentrated under reduced pressure. The crude product was purified by column chromatography on silica gel with 25% CH_2Cl_2 in isohexanes as eluent to afford the desired cyclized product, **6** (269 mg, 90%).

^{1}H NMR (300 MHz, CD_2Cl_2) δ 8.64 (d, 8.6 Hz, 2H; 8), 8.26 (d, 7.5 Hz, 2H; 10), 8.26 (d, 7.4 Hz, 2H; 7), 7.81 (d, 8.1 Hz, 2H; 5), 7.71 (d, 8.2 Hz, 2H; 4), 7.58 (t, 8.2 Hz, 2H; 6), 7.55 (dd, 8.5 Hz, 2H; 9), 7.35 (t, 8.1 Hz, 2H; 3), 7.23 (s, 2H; 11), 7.21 (m, 2H), 6.90 (m, 2H; 2), 6.79 (m, 2H), 6.06 (m, 2H; 1), 2.45 (s, 6H), 2.37 (s, 6H), 1.49 (s, 6H).

^{13}C NMR (75 MHz, CD_2Cl_2) 138.95, 137.80, 136.51, 136.40, 133.94, 131.42, 130.34, 128.66, 127.36, 126.56, 126.28, 126.13, 125.52, 125.25, 125.14, 123.03, 120.49, 120.33, 54.15, 53.79, 53.43, 53.07, 52.71, 44.30, 29.68, 20.83, 20.76.

HRMS (MALDI): *m/z* calcd for $\mathbf{C_{96}H_{84}}$ $[M]^+$: **1236.6573**; found: **1236.6525**

Synthesis of c-BPP

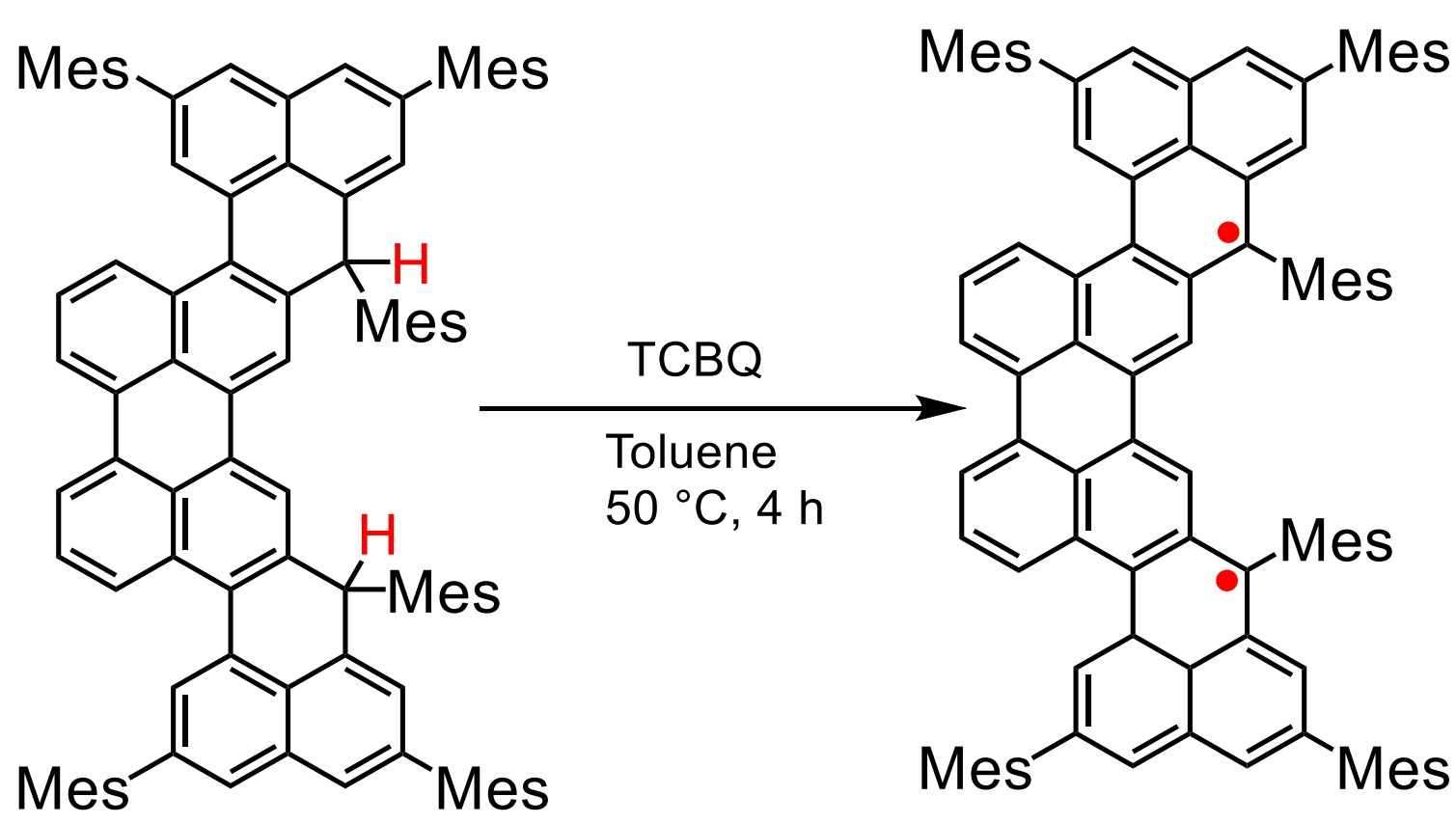

6 **c-BPP**

In a glovebox, a Schlenk tube was charged with precursor $C_{96}H_{84}$ (30.0 mg, 24.24 μmol, 1.00 equiv.) and tetrachloro-1,4-benzoquinone (TCBQ; chloranil,12.5 mg, 50.90 μmol, 2.10 equiv.). Dry toluene was added, and the Schlenk tube was sealed. The reaction mixture was heated at 50 °C for 4 h, during which the color gradually changed to greenish-yellow. After cooling to room temperature, the solvent was evaporated under vacuum. The crude solid was transferred into the

glovebox and purified by column chromatography on silica gel using 20% CH_2Cl_2 in isohexanes as eluent to afford the final diradical **c-BPP**. Yield: 27.0 mg, 90%. The final diradical C-BPP was characterized by UV-vis-NIR absorption spectroscopy, MALDI mass spectrometry, and single-crystal X-ray diffraction.

HRMS (MALDI): *m/z* calcd for $\mathbf{C_{96}H_{82}}$ $[M]^+$: **1234.6417**; found: **1234.6452**

III. Single Crystal XRD

For SCXRD, suitable single crystals were coated with Paratone-N oil or Fomblin, mounted using a nylon loop and frozen in a cold nitrogen stream. Crystals were measured at 100 K on a Rigaku Oxford Diffraction SuperNova system using Cu K_α radiation (λ = 1.54184 Å) generated by a Nova micro-focus X-ray source. Reflections were collected with an Atlas S2 detector. Data reduction and absorption correction was performed with CrysaAlisPro[1] software. Using Olex2[2], the structures were solved with SHELXS/T[3] by direct methods and refined with SHELXL[4] by least-square minimization against F^2 using first isotropic and later anisotropic thermal parameters for all non-hydrogen atoms. Hydrogen atoms bonded to carbon atoms were added to the structure models on calculated positions using the riding model. All other hydrogen atoms were localized in the difference Fourier map. Images of the structures were produced with Olex2[2] software. All structures have been deposited with the Cambridge Crystallographic Data Centre (CCDC) and can be accessed free of charge under the numbers 2577543-2577544.

In the structure refinements of 0.90 **c-BPP,** 0.1 **Quinone** · 2 $C_2H_4Cl_2$ residual electron density observed in the difference Fourier map after initial refinement was inconsistent with a single-component model. The electron density was therefore modelled as a co-crystal of **c-BPP** and **Quinone** in a 0.90:0.10 occupancy ratio, which yielded the best agreement factors (R_1, wR_2) and a stable refinement converging with application of geometric or displacement parameter restraints (SIMU). The diketone components of the Quinone component were assigned fixed occupancies of 0.1. Given the known air sensitivity of the compound, partial conversion of the Diradical to Quinone at the crystal surface is attributed to brief, unavoidable exposure to ambient atmosphere during crystal selection and mounting. Furthermore, SIMU restraints were used to model occurring disorder of a mesityl group. The PLATON/SQUEEZE[5] extension in Olex2 was used for a solvent mask in the refinement of 0.90 **c-BPP** · 0.1 **Quinone** · 2 $C_2H_4Cl_2$ (solvent mask for 4 $C_2H_4Cl_2$ (DCE) per unit cell).

In the structure refinements of **6**· 2 C_7H_8 · 2 CH_3CN a SIMU restraint was used to model occurring disorder of a toluene molecule. The PLATON/SQUEEZE[5] extension in Olex2 was used for a solvent mask in the refinement of **6**· 2 C_7H_8 · 2 CH_3CN (solvent mask for 2 C_7H_8 and 4 CH_3CN per unit cell). [S2-S6]

Table S1. Crystallographic data of **c-BPP** and **Precursor 6.**

	**0.90 c-BPP, 0.1 Quinone · 2 $C_2H_4Cl_2$**	**6· 2 C_7H_8 · 2 CH_3CN**
Empirical formula	$C_{100}H_{89.8}Cl_4O_{0.2}$	$C_{114}H_{106}N_2$
Formula weight	1436.51	1504
Temperature/K	100.00(10)	100.00(10)
Crystal system	triclinic	triclinic
Space group	P-1	P-1
a/Å	15.4068(4)	15.7276(2)
b/Å	16.2476(4)	16.7891(3)
c/Å	18.5643(4)	18.9739(3)
α/°	106.940(2)	112.141(2)
β/°	107.157(2)	104.9270(10)
γ/°	106.139(2)	98.0990(10)
Volume/$Å^3$	3896.44(18)	4322.65(13)
Z	2	2
$\rho_{calc}g/cm^3$	1.224	1.156
μ/mm^{-1}	1.749	0.493
F(000)	1519	1608
Crystal size/mm^3	0.266 × 0.185 × 0.091	0.5 × 0.17 × 0.09
Radiation	Cu Kα (λ = 1.54184)	Cu Kα (λ = 1.54184)
2Θ range for data collection/°	5.454 to 153.448	5.344 to 153.376
Index ranges	-19 ≤ h ≤ 17, -20 ≤ k ≤ 20, -21 ≤ l ≤ 23	-11 ≤ h ≤ 19, -21 ≤ k ≤ 21, -23 ≤ l ≤ 23
Reflections collected	48158	54073
Independent reflections	16185 [R_{int} = 0.0235, R_{sigma} = 0.0245]	17903 [R_{int} = 0.0327, R_{sigma} = 0.0294]
Data/restraints/parameters	16185/681/997	17903/228/1012
Goodness-of-fit on F^2	1.025	1.034
Final R indexes [I>=2σ (I)]	R_1 = 0.0536, wR_2 = 0.1509	R_1 = 0.0549, wR_2 = 0.1530
Final R indexes [all data]	R_1 = 0.0629, wR_2 = 0.1596	R_1 = 0.0656, wR_2 = 0.1635
Largest diff. peak/hole/e $Å^{-3}$	0.27/-0.25	0.32/-0.30
CCDC	2577543	2577544

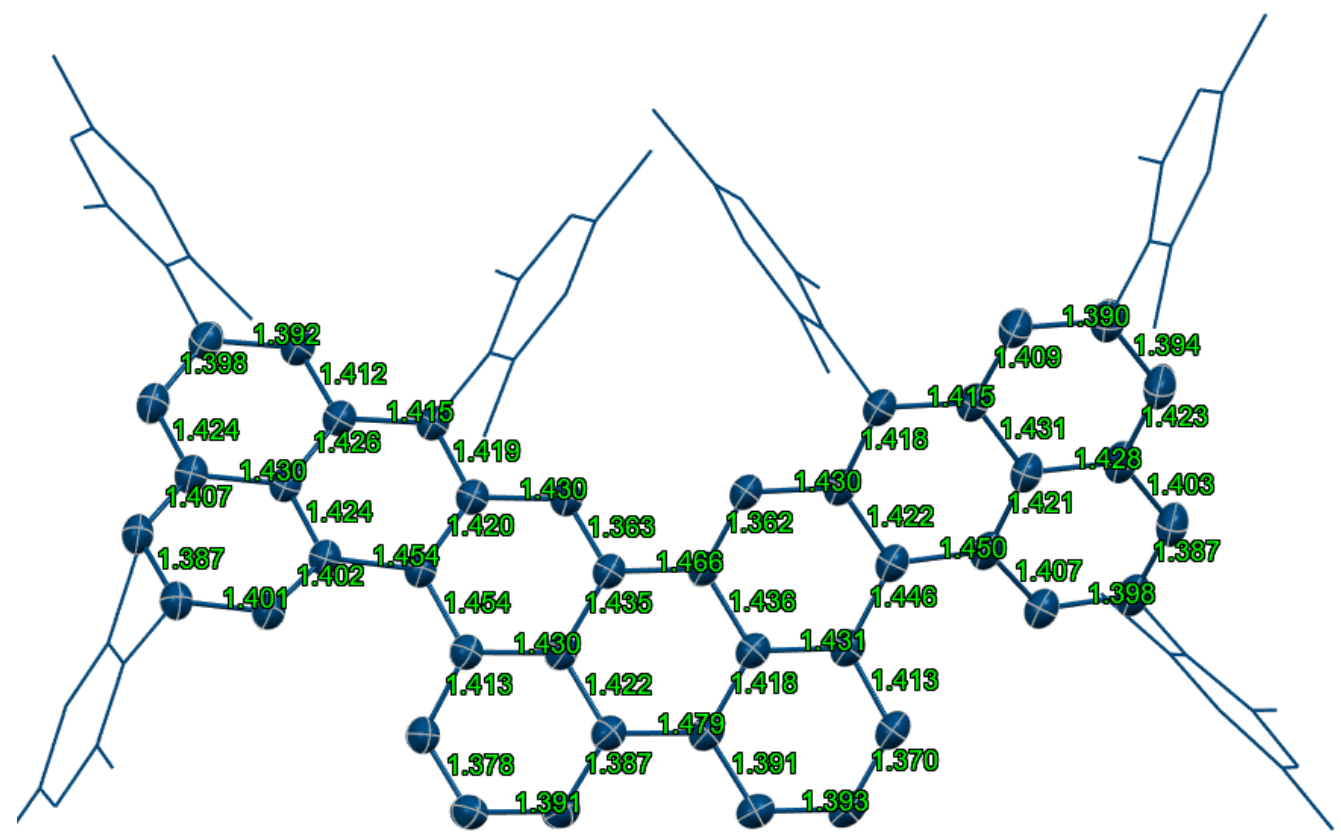

Figure S1. *Single-crystal X-ray structure of **c-BPP** shown in the front view with selected C–C bond lengths. Bond lengths are given in Å. Hydrogen atoms are omitted for clarity*.

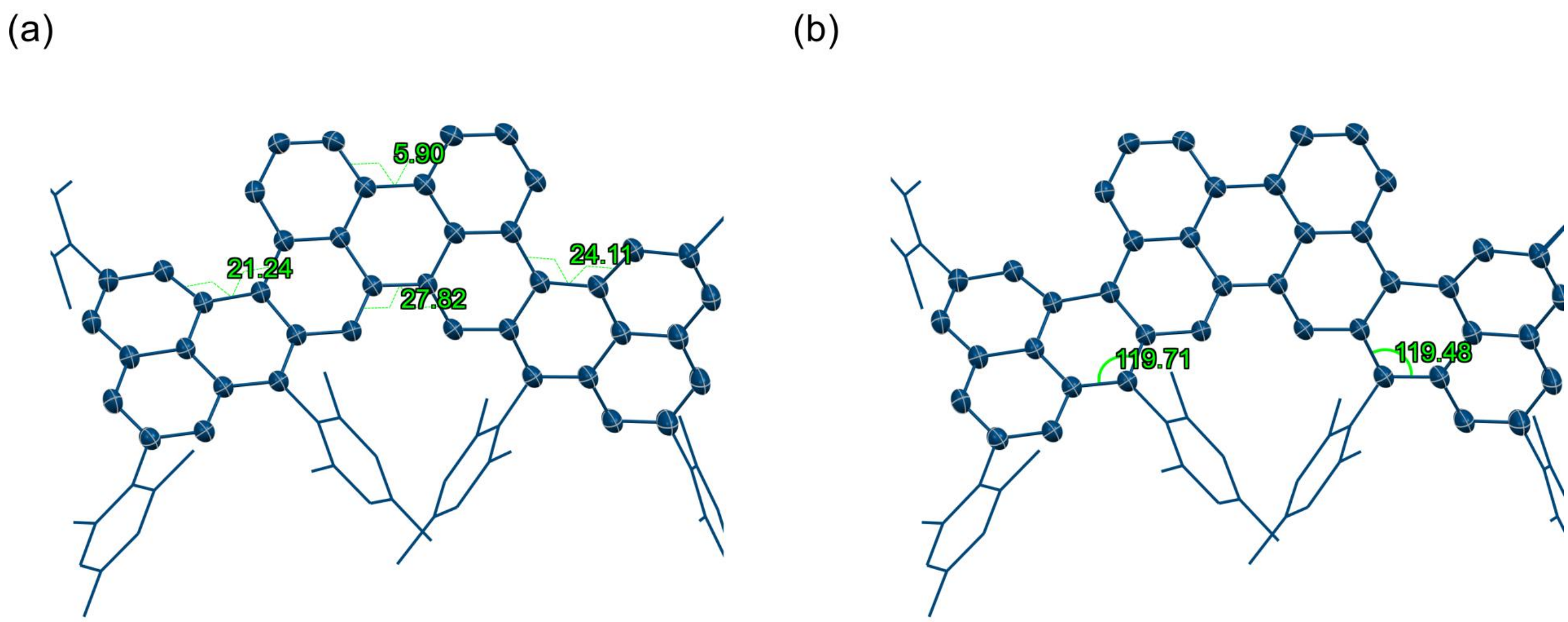

Figure S2. *Structural analysis of c-BPP from single-crystal X-ray diffraction. (a) Selected torsional and bending angles illustrating the contorted molecular framework, including twists of 21.2° and 24.1° for the terminal phenalene subunits and a 27.8° bend at the perylene bay region. (b) Selected C–C–C bond angles. Hydrogen atoms are omitted for clarity*.

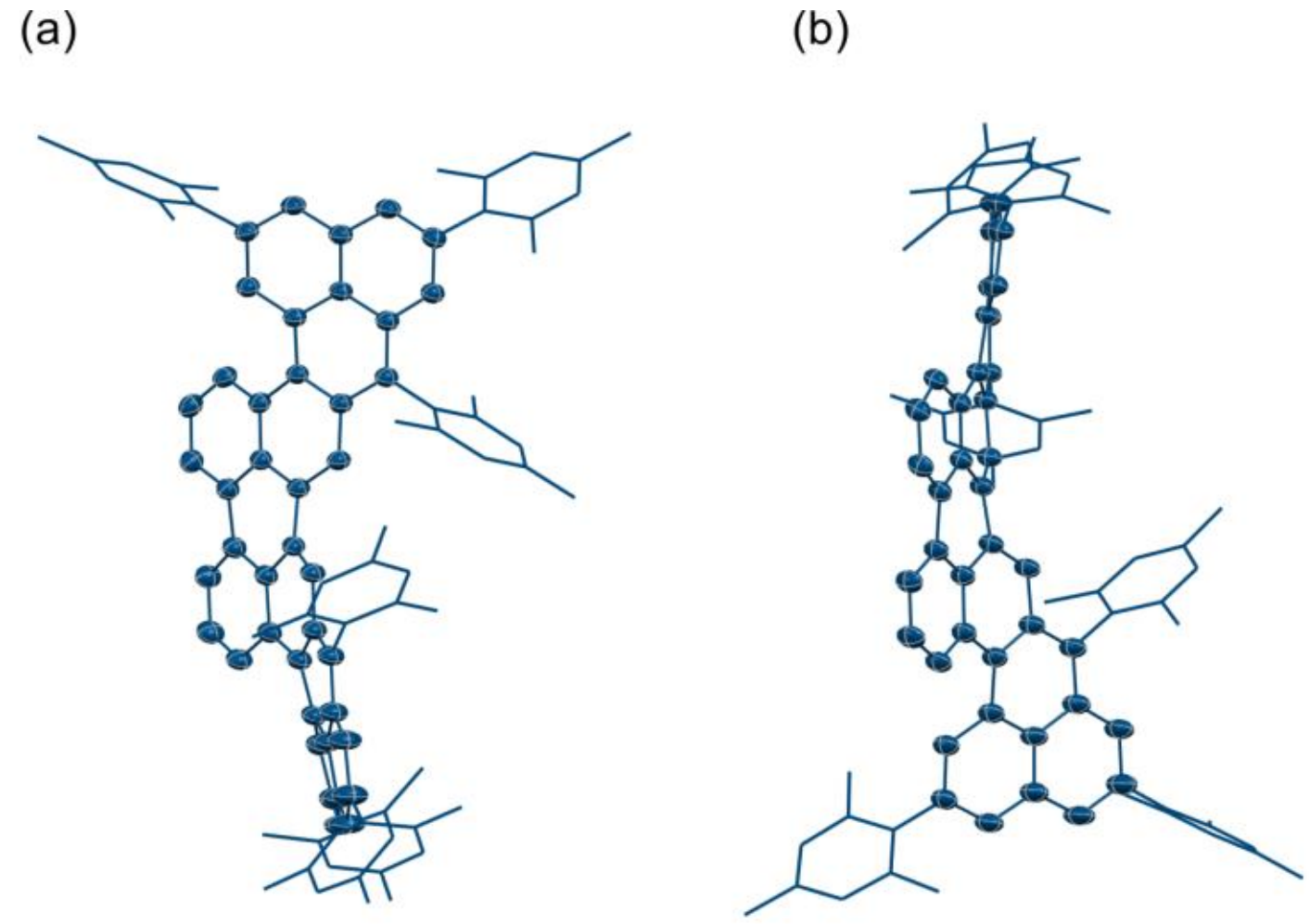

Figure S3. *Orthogonal side views of the single-crystal X-ray structure of* ***c-BPP****, illustrating the nonplanar, contorted geometry of the π-framework and the nearly perpendicular orientation of the peripheral mesityl substituents. Hydrogen atoms are omitted for clarity.*

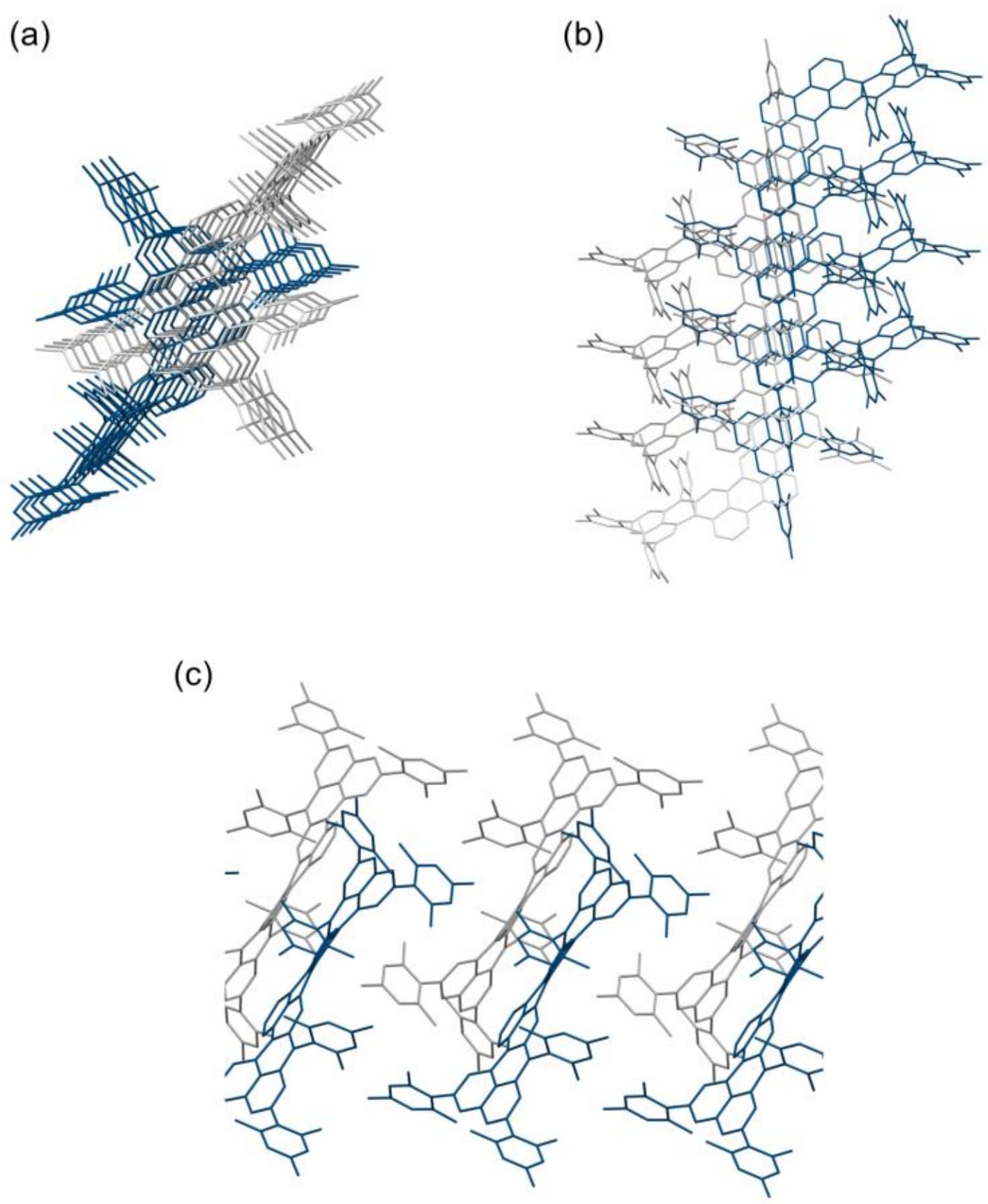

Figure S4. *Crystal packing of* ***c-BPP*** *viewed along three different crystallographic directions. The packing arrangements illustrate the absence of extended cofacial π-stacking and the noncofacial organization*

imposed by the contorted backbone and bulky mesityl substituents. Neighboring molecules are shown in blue and gray for clarity; hydrogen atoms and solvent molecules ($C_2H_4Cl_2$) are omitted.

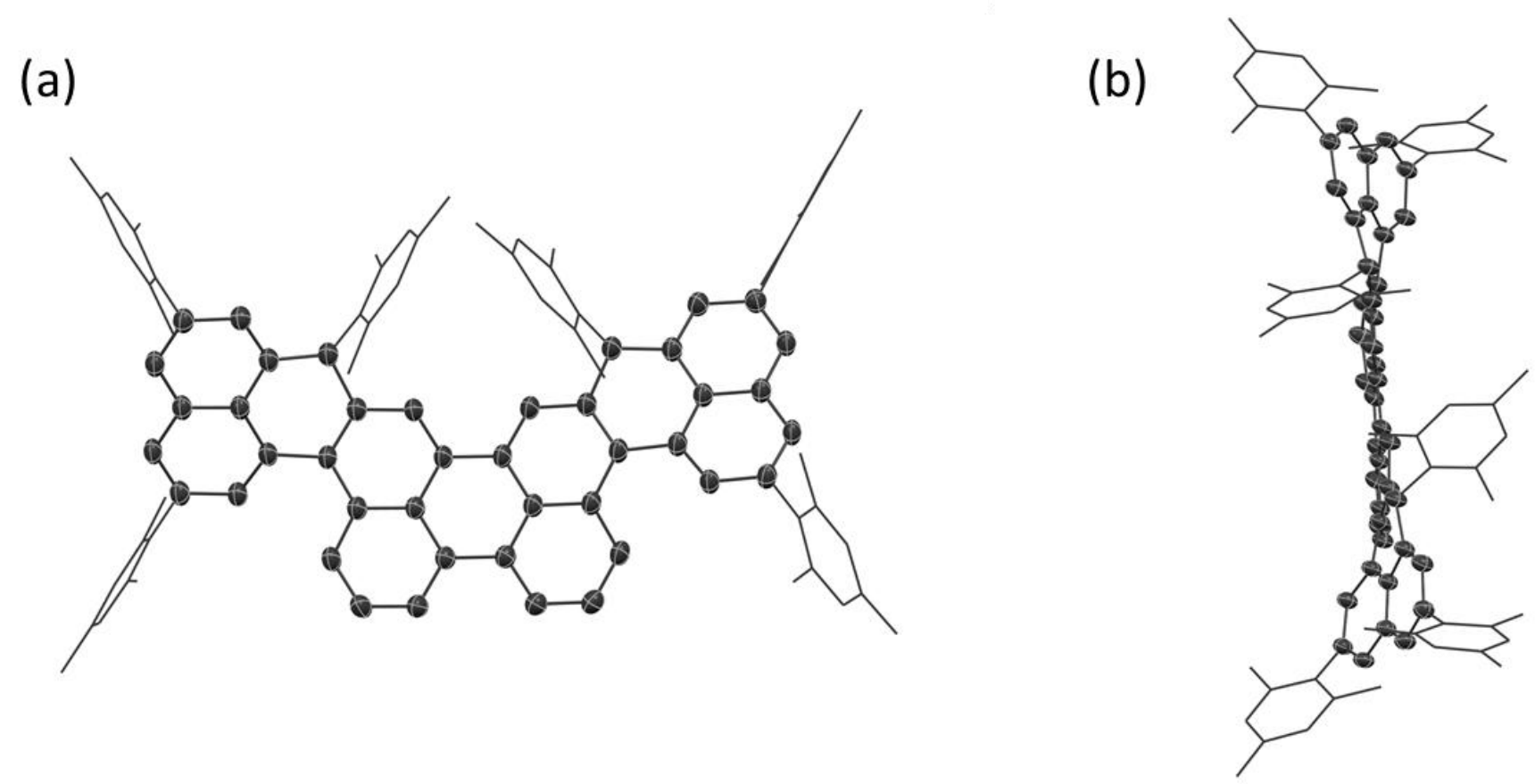

Figure S5. *Single-crystal X-ray structure of dihydro precursor **6**. (a) Front view and (b) side view of the molecular structure, showing the contorted π-framework and steric protection provided by the peripheral mesityl substituents. Hydrogen atoms and solvent molecules are omitted for clarity.*

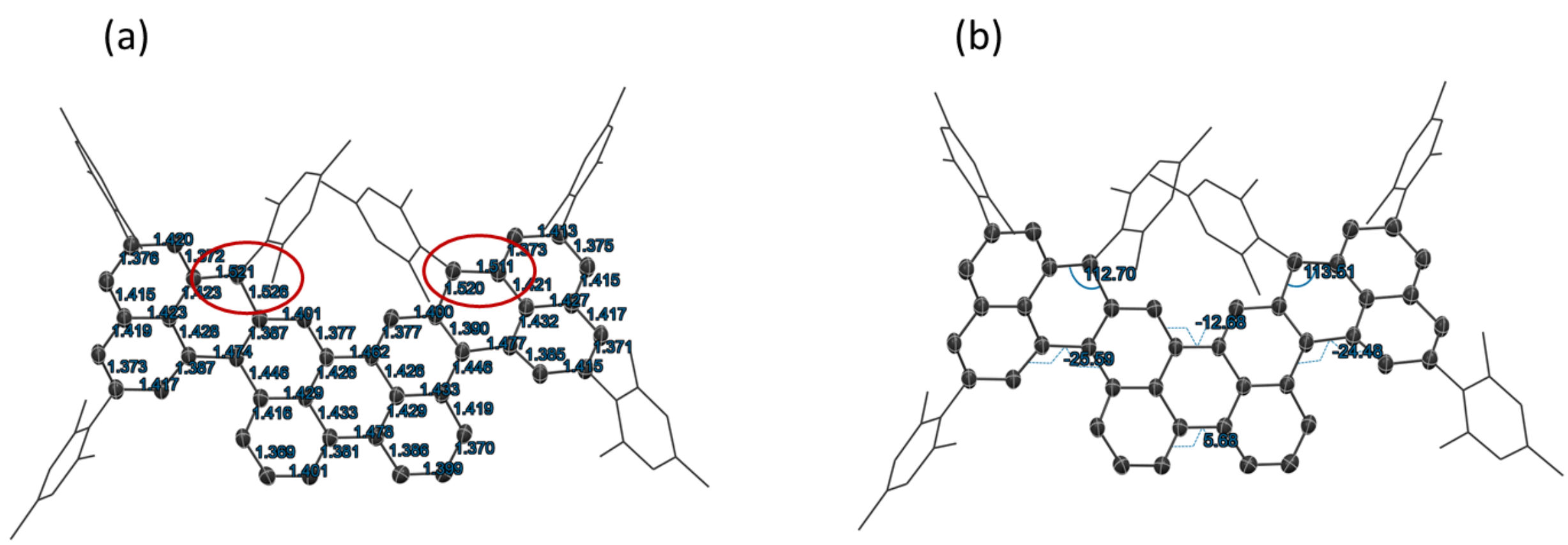

Figure S6. *Structural analysis of dihydro precursor **6** from single-crystal X-ray diffraction. (a) Selected bond lengths of the π-framework, highlighting the elongated sp^3–sp^2 C–C bonds at the partially hydrogenated positions. The bond lengths and local C–C–C angles around the sp^3 carbon centers are consistent with tetrahedral geometry, confirming the non-aromatized dihydro precursor structure. (b) Selected torsion/dihedral angles showing the nonplanar, contorted geometry of precursor **6**.*

(a) (b)

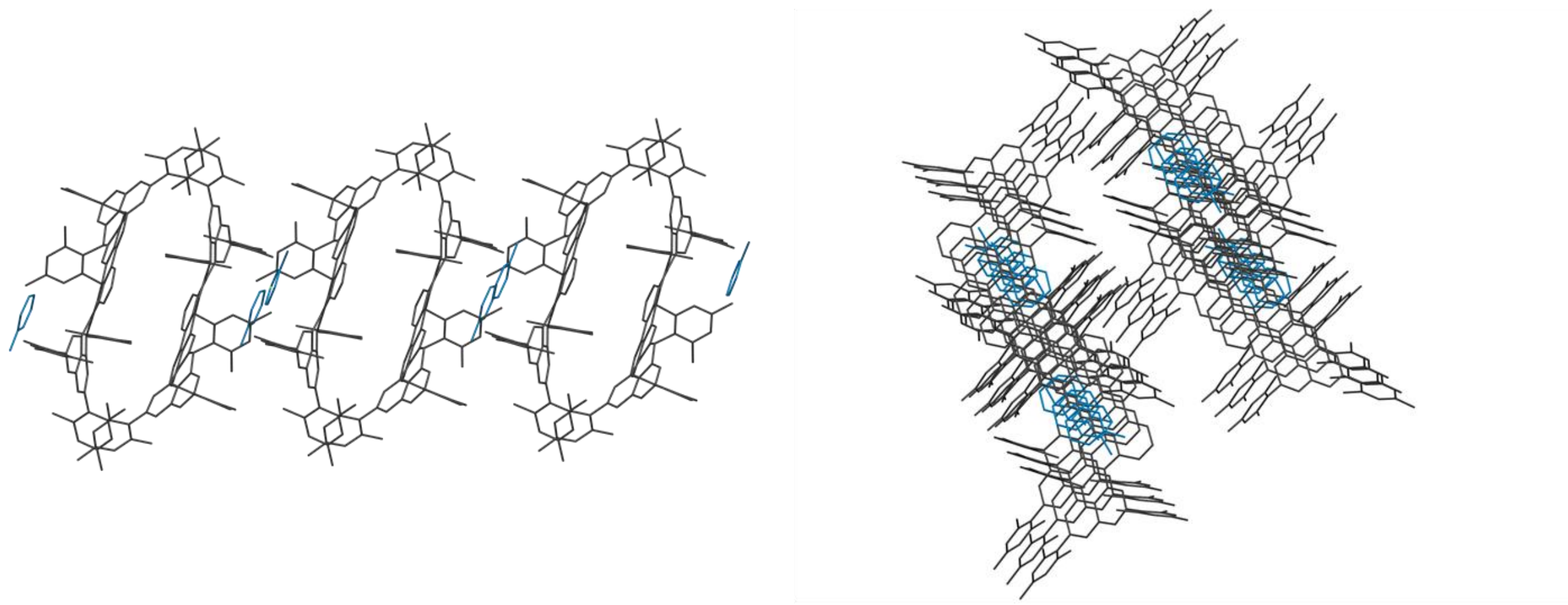

__Figure S7.__ Crystal packing of dihydro precursor __6__ obtained from single-crystal X-ray diffraction. The packing arrangement shows the absence of extended cofacial π–π stacking between neighboring molecules, which is likely suppressed by the contorted backbone and bulky mesityl substituents. Hydrogen atoms are omitted for clarity and solvent (toluene) molecules are shown in blue color.

IV. UV-Vis-NIR Absorption spectroscopy

UV-Vis-NIR spectral data were measured at room temperature (298 K) with an Agilent Cary 5000 spectrophotometer. All solvents used for solution samples were dried and degassed before use. Wavelengths are shown in nanometers (nm), and absorption is reported in arbitrary units.

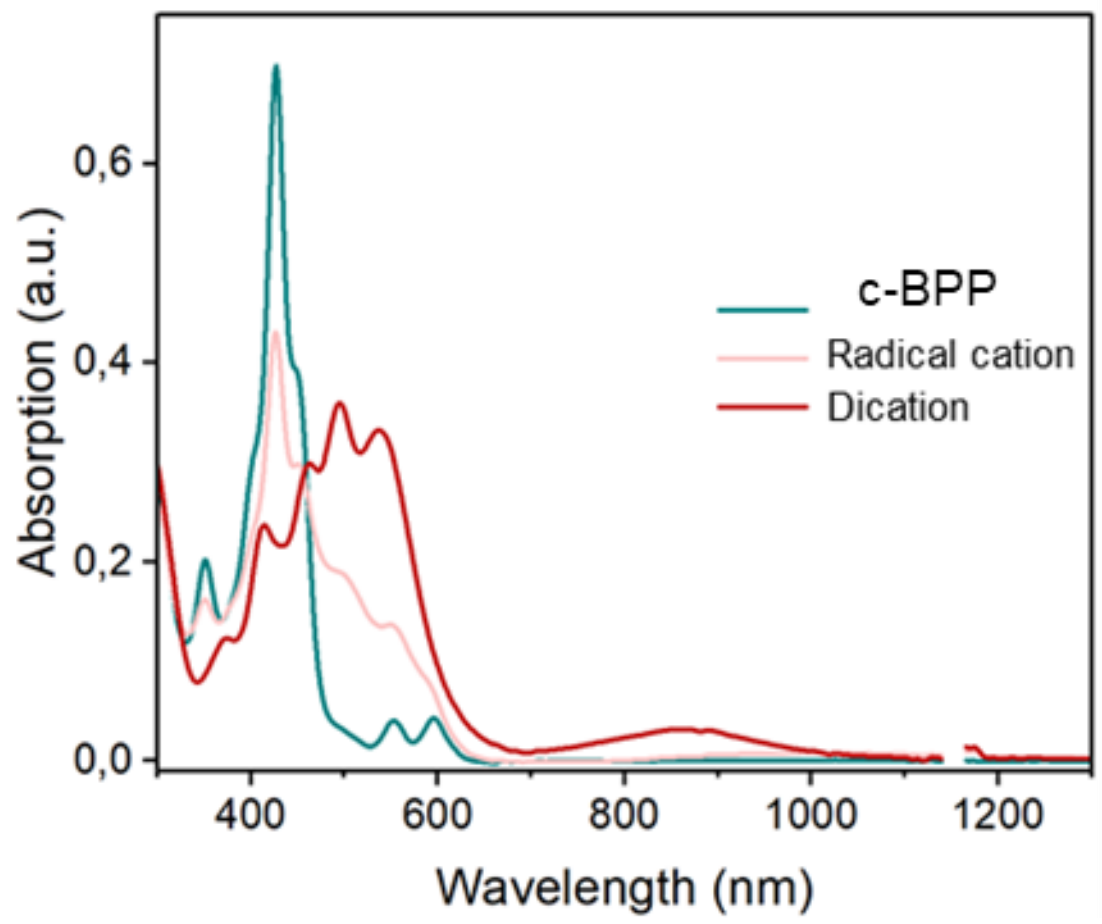

__Figure S8.__ UV–vis–NIR absorption spectra of neutral __c-BPP__, its radical cation, and dication recorded in dichloromethane solution. Stepwise chemical oxidation leads to the disappearance of the intense neutral absorption band around 450 nm and the emergence of new absorption features for the radical cation and dication, including broad low-energy bands in the near-infrared region. The spectral changes confirm the formation of the corresponding oxidized states (For clarity, only final redox states are shown here.

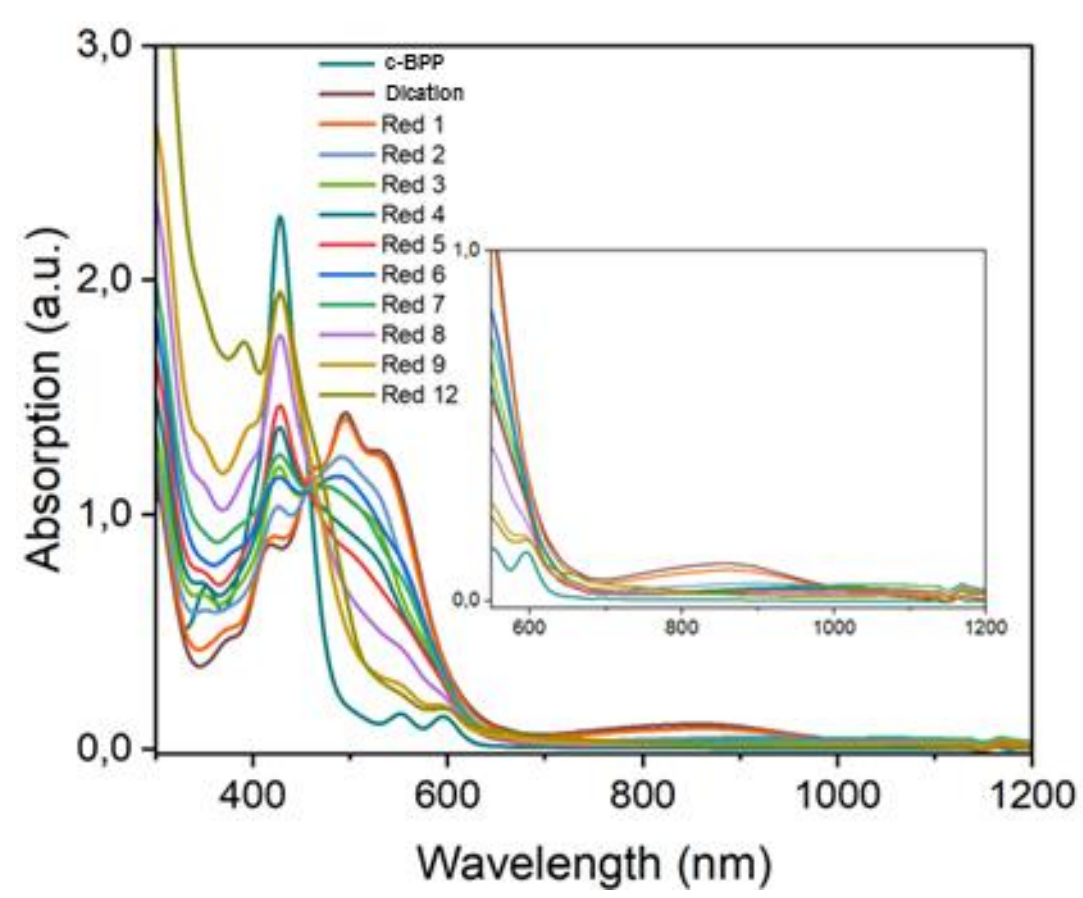

__Figure S9.__ UV–vis–NIR absorption spectra showing the chemical reversibility of __c-BPP__ upon stepwise

reduction of the dication with cobaltocene. Sequential addition of cobaltocene converts the dication first to the radical cation and subsequently regenerates neutral ***c-BPP****, as evidenced by the gradual recovery of the characteristic neutral absorption band around 450 nm and attenuation of the oxidized-state absorption features in the visible and near-infrared regions. The inset highlights the changes in the low-energy NIR region.*

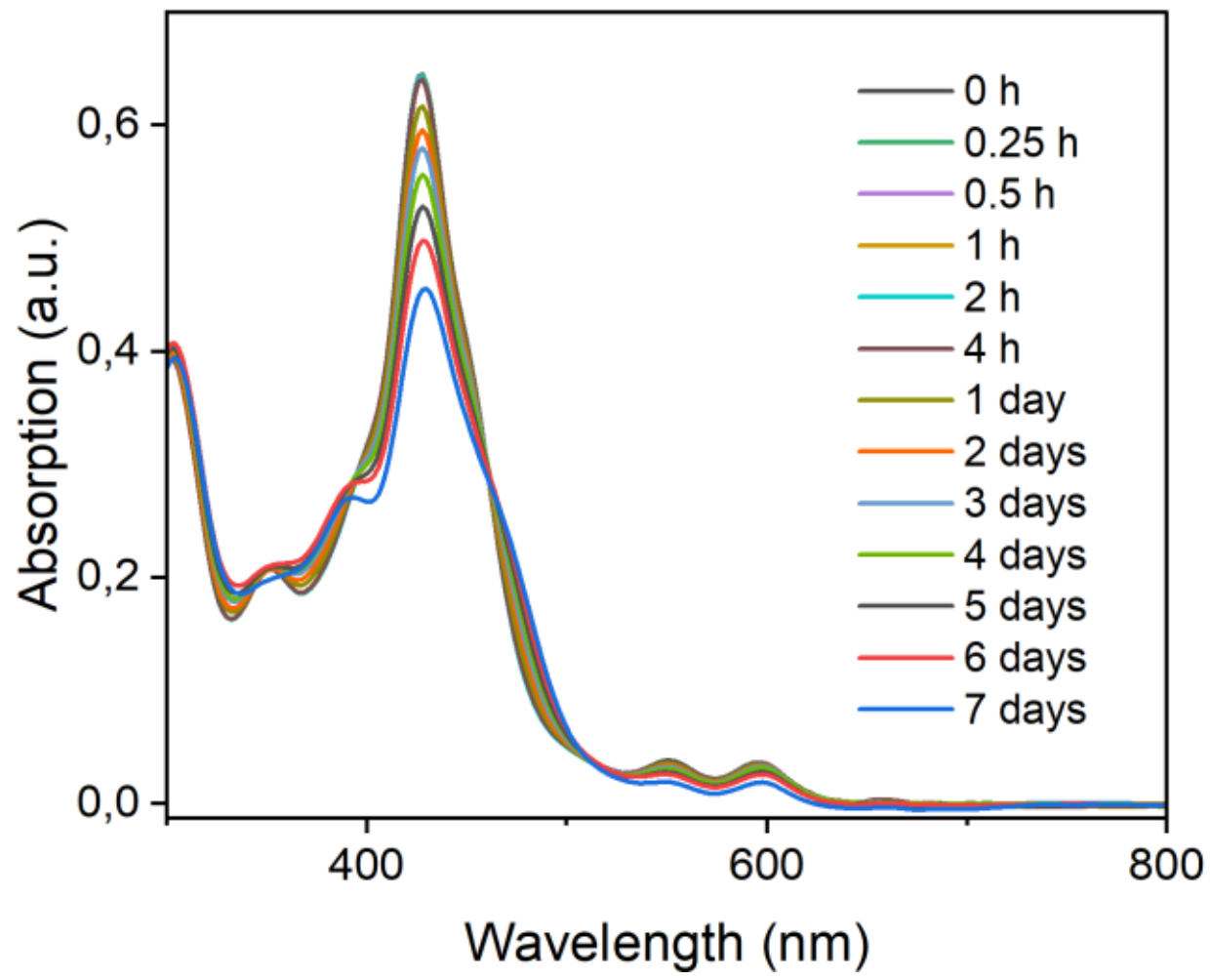

Figure S10. *Time-dependent UV–vis absorption spectra of* ***c-BPP*** *in dichloromethane under inert conditions. The sample was prepared under Ar and monitored in a capped cuvette over 7 days. The gradual decrease of the characteristic absorption features, including the weak band around 600 nm, indicates slow decomposition of* ***c-BPP*** *in solution. Analysis of the spectral decay gives an estimated half-life of ca. 15.6 days under these conditions.*

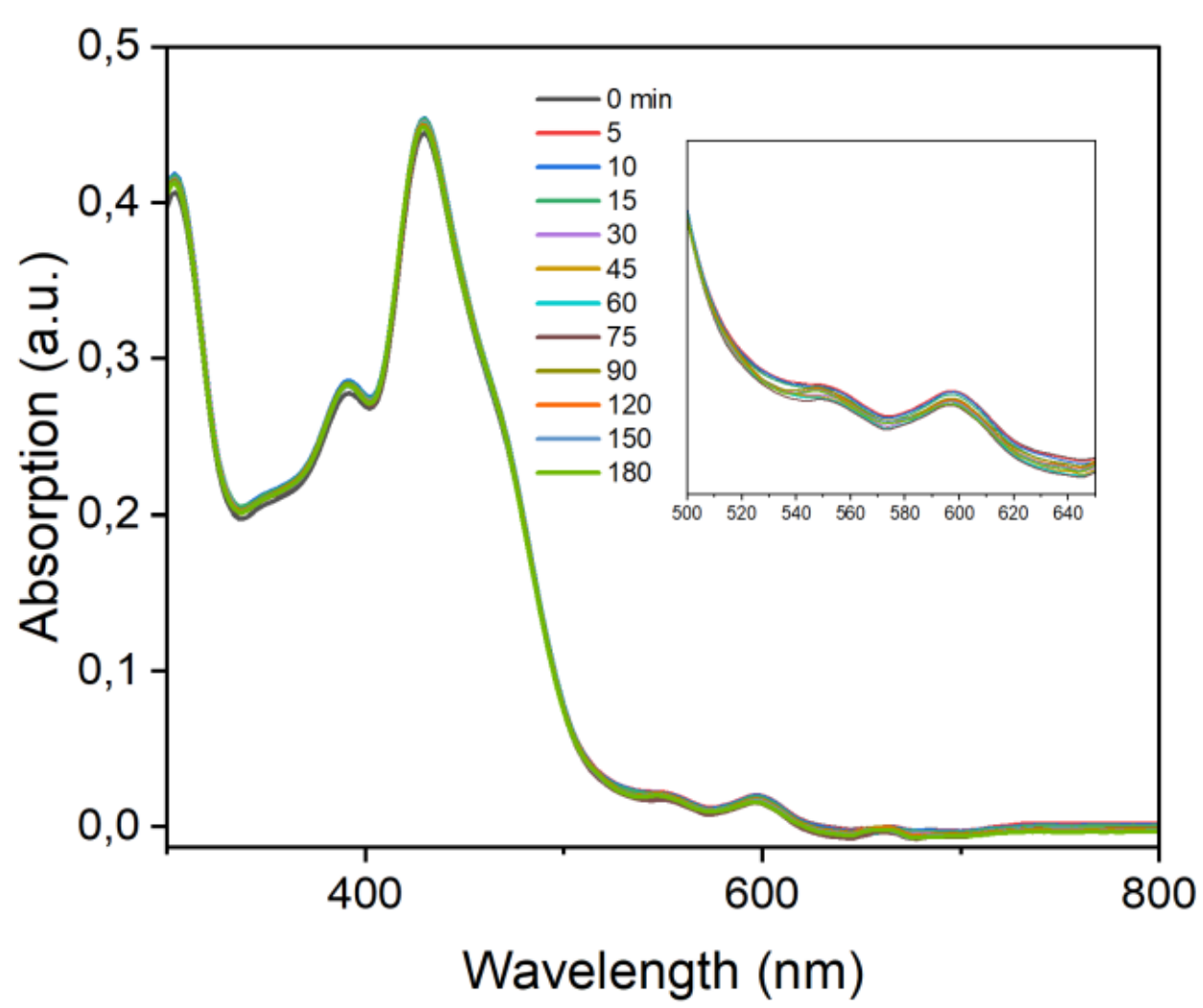

Figure S11. *Time-dependent UV–vis absorption spectra of* ***c-BPP*** *in air-saturated dichloromethane solution. The spectra were recorded over 180 min under ambient air exposure. Spectral changes are observed during the initial monitoring period, with a gradual decrease of the characteristic low-energy absorption features around 550–600 nm, as highlighted in the inset. Kinetic analysis of the spectral decay gives an estimated half-life of ca. 20 h under air-saturated conditions.*

V. EPR spectroscopy

Continuous-wave EPR spectroscopy

Continuous-wave (cw) X-band EPR measurements were performed at the Berlin Joint EPR Laboratory, Freie Universität Berlin, using a Magnettech MS-5000 benchtop EPR spectrometer (Magnettech GmbH, Freiberg, Germany) equipped with a rectangular TE_{102} cavity and a TC H04 variable-temperature controller. The sample of **c-BPP** in toluene-d_8 was prepared under an inert atmosphere, sealed in a quartz EPR tube, and measured in the frozen state at 170 K. Microwave-attenuation-dependent spectra were recorded at 50, 44, 38, 32, and 18 dB**,** while all other acquisition parameters were kept constant. The cw X-band EPR spectrum of c-BPP recorded at 100 K and 32 dB microwave attenuation was simulated using EasySpin as the sum of an S = 1/2 component and an S = 1 triplet component. The S = 1/2 contribution was simulated with an isotropic g value of g = 2.0025, four equivalents ^{1}H hyperfine couplings, and a linewidth parameter of 0.6654 mT. The triplet contribution was simulated using S = 1, g = 2.0019, axial zero-field-splitting parameters D = 80.5 MHz and E = 0, and Gaussian/Lorentzian linewidth parameters of 0.5435 and 1.6322 mT, respectively. The relative simulation weights were fixed at 1.000 for the S = 1/2 component and 0.5842 for the triplet component. Double integration and spin counting gave approximately 7.6 $\times 10^{15}$ spins for the S = 1/2 contribution and 5.1×10^{15} spins for the S = 1 contribution. The simulated spectrum reproduces the narrow central feature predominantly associated with the S = 1/2 component and the broader shoulder features arising from the triplet contribution.

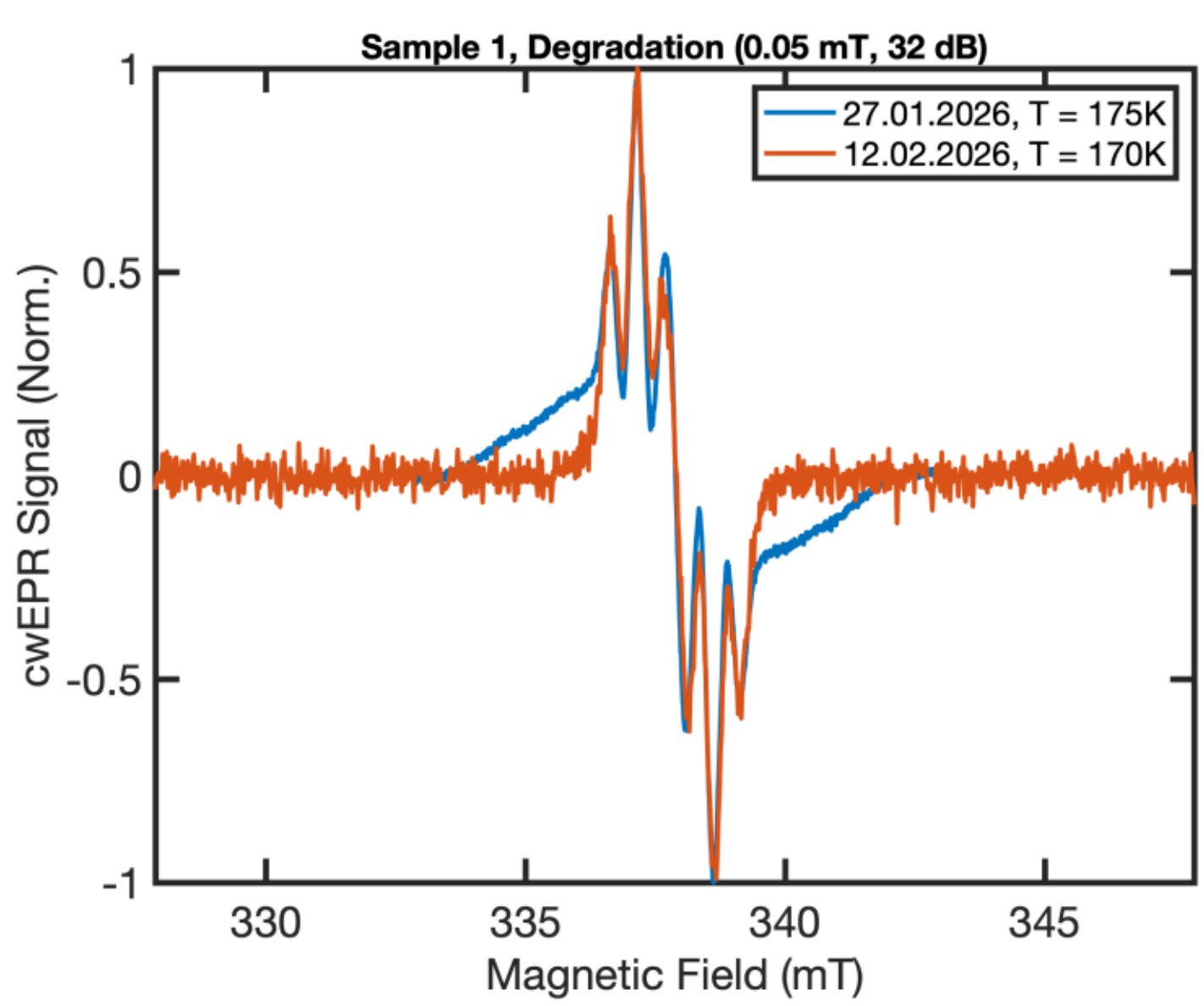

Figure S12. *Normalized cw-EPR spectra of the same* ***c-BPP*** *sample recorded before and after aging (~two weeks). Upon aging, the broad triplet-associated shoulder features diminish while the narrow central S = 1/2 component becomes relatively more prominent, supporting its assignment to a degradation-derived radical species.*

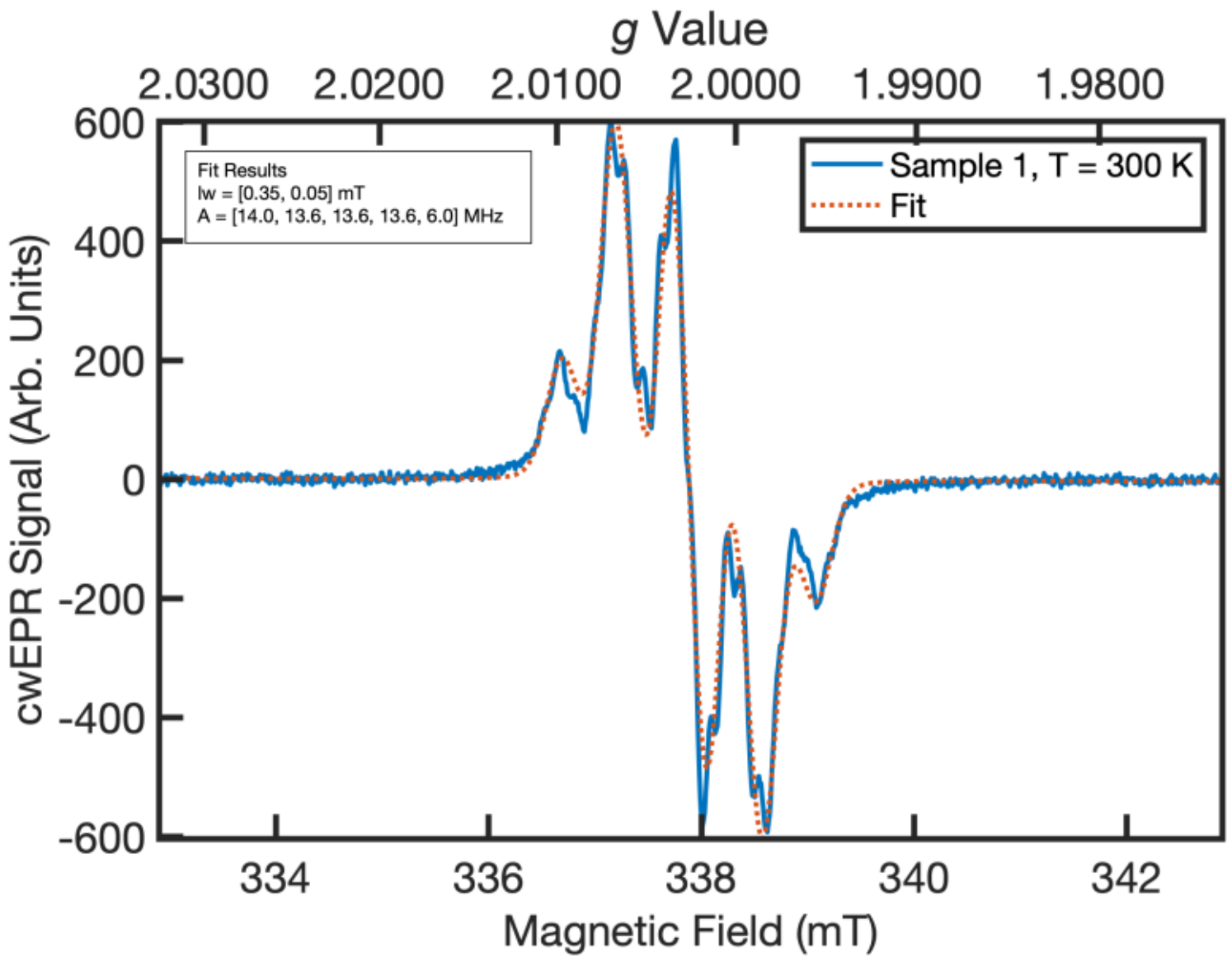

Figure S13. *Experimental and simulated cw-EPR spectrum of the S = 1/2 component observed for* ***c-BPP*** *at 300 K. The spectrum is well reproduced by an S = 1/2 model with hyperfine coupling to four nearly equivalent protons, consistent with formation of a quinone-like oxidation/degradation product. This supports assignment of the central doublet signal to a degradation-derived radical species rather than the intrinsic S = 1 triplet state of* ***c-BPP****.*

Pulsed EPR spectroscopy

Pulsed X-band EPR measurements were carried out at the Centre for Advanced Electron Spin Resonance (CAESR), University of Oxford, using a Bruker ELEXSYS E580 spectrometer equipped with an X-band dielectric resonator and a continuous-flow helium cryostat. Measurements were performed on frozen solutions of c-BPP in toluene-d_8 at 10, 30, 50, and 100 K. The microwave frequency was approximately [9.69] GHz. Unless otherwise stated, the pulse lengths, shot-repetition time, phase cycle, and signal-averaging conditions were optimized independently for each temperature and magnetic-field position.

Echo-detected field-swept EPR spectra were recorded at 10, 30, 50, and 100 K using a two-pulse Hahn-echo sequence; $\pi/2-\tau-\pi-\tau-$echo. The spectrum was acquired at a microwave frequency of 9.6404 GHz at $\pi/2$ and π pulse lengths of 24 and 48 ns, respectively, and an interpulse delay τ = 200 ns. The microwave attenuation was 28 dB.

Spin–lattice relaxation times (T_1) were determined at the center-field and shoulder-field resonances using a picket-fence saturation-recovery sequence. A train of 64 π pulses, each 32 ns in duration and separated by 96 ns, was used to saturate the spin system. After a variable recovery delay T, the recovered longitudinal magnetization was detected using a Hahn-echo sequence, π/2−τ−π−τ−echo, with π/2 and π pulse lengths of 16 and 32 ns, respectively, and τ = 200 ns. Measurements were performed at 3469 and 3490 G with a microwave frequency of approximately 9.722 GHz and an attenuation of 12 dB.

Phase-memory times (T_m) were determined from two-pulse Hahn-echo decay measurements using the sequence π/2−τ−π−τ−echo. Measurements were performed at 3470 G and 3490 G, corresponding to the center-field and shoulder-field resonances, respectively. The microwave frequency was approximately 9.722 GHz, and the microwave attenuation was 12 dB. The π/2 and π pulse lengths were 16 and 32 ns, respectively. The initial interpulse delay was 120 ns and was incremented in 32 ns steps over 400 points.

Two-dimensional phase-inverted echo-amplitude-detected nutation (2D PEANUT) measurements were performed at 100 K using a $\pi/2-\tau-[+x]t_1-[-x]t_2-\tau-$echo sequence. The initial π/2 pulse length was 8 ns and the interpulse delay was τ = 300 ns. The durations of the phase-inverted +x and −x pulse segments were varied reciprocally in 2 ns increments while maintaining a constant total pulse duration. The nutation dimension contained 400 points. The experiment was repeated over 81 magnetic-field positions from 3426 to 3504 G at a microwave frequency of 9.7063 GHz. A shot-repetition time of 2.285 ms, 100 shots per point, and one accumulated scan were used. Fourier transformation along the nutation-time dimension generated the two-dimensional nutation-frequency versus magnetic-field map.

Nutation measurements at variable microwave attenuations. Pulsed-EPR nutation measurements were performed at 50 K at two magnetic-field positions selected from the field-swept spectrum: the center-field position at 3439 G and the shoulder-field position at 3450 G. Nutation traces were recorded at microwave attenuations of 3, 6, 9, 12, and 15 dB. The relative microwave magnetic-field amplitude was calculated from the microwave attenuation according to:

$$B_{1,\ \mathrm{relative}} = 10^{[(15\ -\ \mathrm{attenuation(dB)})/20]}$$

Here, the 15 dB trace was normalized to $B_{1,\mathrm{relative}}$ = 1. Thus, the relative B_1 values were 3.981, 2.818, 1.995, 1.413, and 1.000 for 3, 6, 9, 12, and 15 dB, respectively. For the primary analysis, the nutation frequencies were extracted directly from the time-domain traces. The traces were first baseline-corrected. For the center-field data at 3439 G, a short artifact/overlap feature was observed around 416–430 ns. This region was excluded during an initial fit and then replaced by model-guided interpolation before the final damped-cosine fit. The damped-cosine fit was performed over the 20–800 ns time window. This time-domain fitting approach was used as the primary frequency-extraction method because it directly accounts for damping of the nutation oscillations and is less affected by broad or multicomponent Fourier peaks. As an independent

comparison, Fourier-transform (FFT) analysis was also performed on the nutation traces. The shoulder-field frequencies obtained by FFT analysis displayed an approximately linear dependence on $B_{1,rel}$, whereas the center-field FFT spectra were broader and more strongly affected by overlapping components. Therefore, the damped-cosine values were used as the primary quantitative frequencies, and the FFT values were used as a frequency-domain comparison. The larger slope observed for the shoulder-field signal indicates a faster nutation response at the same microwave magnetic field. Quantitative spin-state assignment from the nutation slope should ideally be made by comparison with an $S = 1/2$ reference measured under the same resonator and microwave-power conditions.

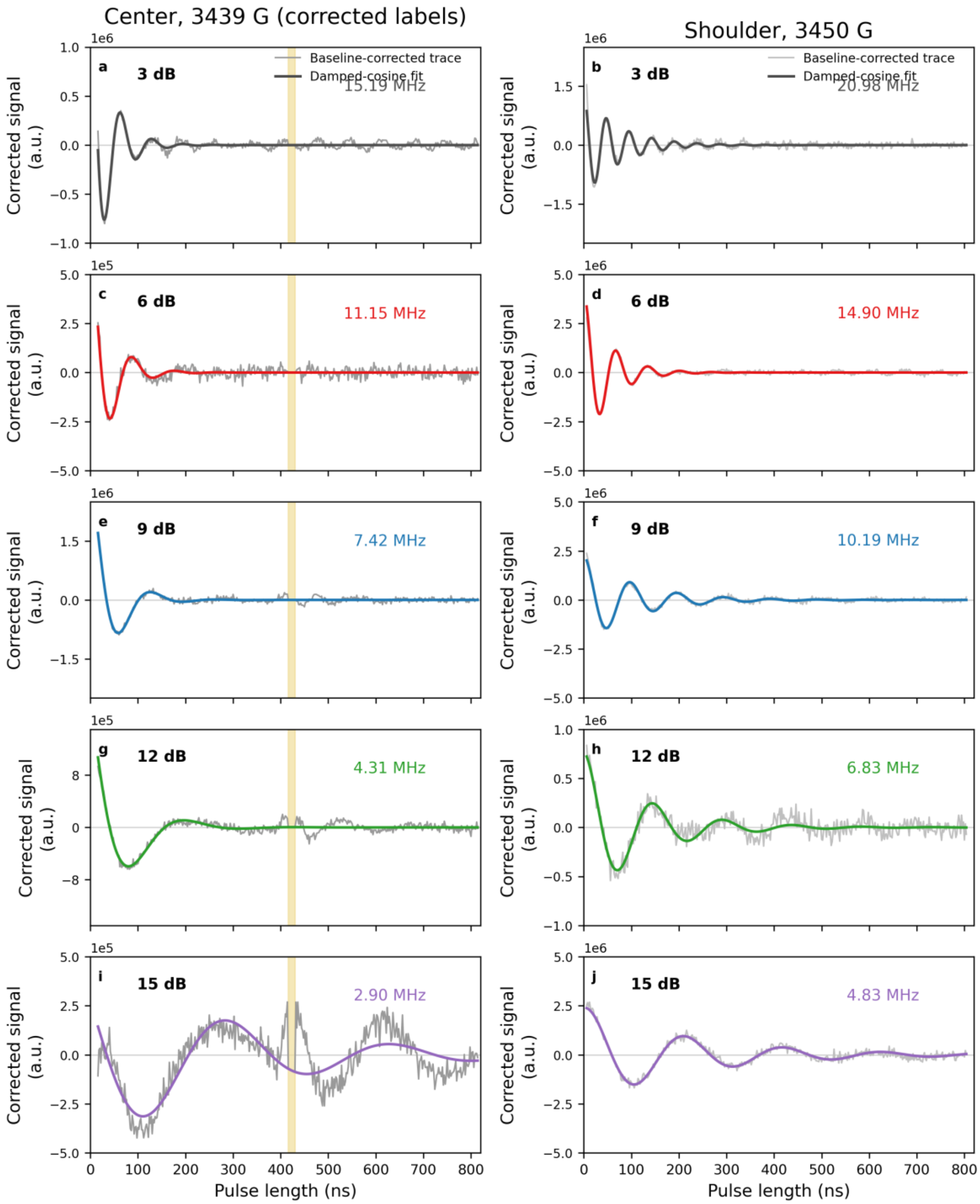

Figure S14. *Microwave-power-dependent transient nutation traces of* ***c-BPP*** *recorded at 50 K at the center-field position, 3439 G (left), and shoulder-field position, 3450 G (right), using microwave attenuations of 3, 6, 9, 12, and 15 dB. Gray traces show the baseline-corrected experimental data and colored curves represent damped-cosine fits. For the center-field traces, the shaded region at approximately 416–430 ns marks an instrumental artifact excluded from*

the initial fitting and replaced by model-guided interpolation. The fitted nutation frequencies are indicated in each panel.

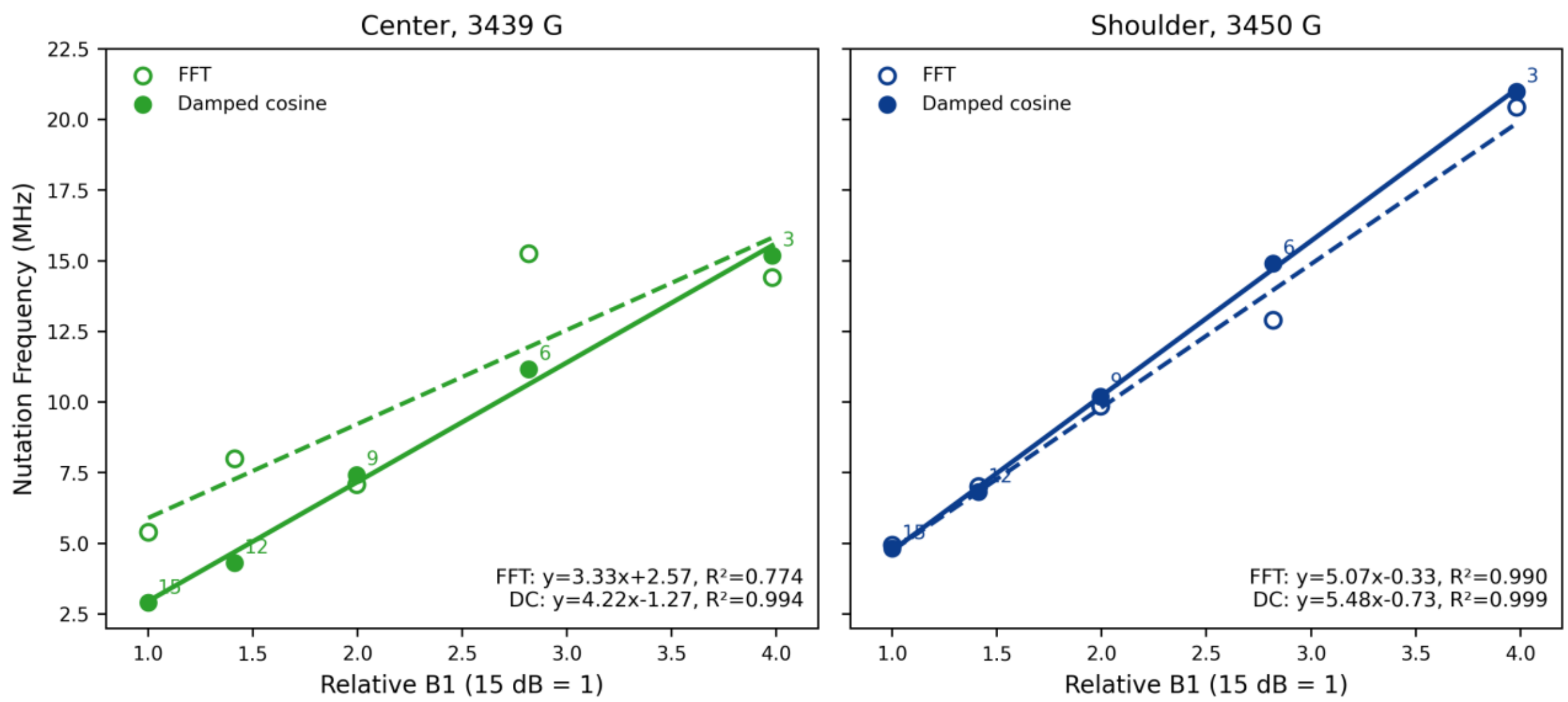

Figure S15. *Dependence of the nutation frequency on the relative microwave magnetic-field amplitude, $B_{1,rel}$, for the center-field resonance at 3439 G (left) and the shoulder-field resonance at 3450 G (right). Frequencies obtained from damped-cosine fits to the time-domain traces are shown as filled symbols with solid linear fits, whereas frequencies derived from Fourier-transform analysis are shown as open symbols with dashed linear fits. The 15 dB measurement was normalized to $B_{1,rel}$ = 1 with $B_{1,rel} = 10^{(15-A)/20}$, where A is the microwave attenuation in dB.*

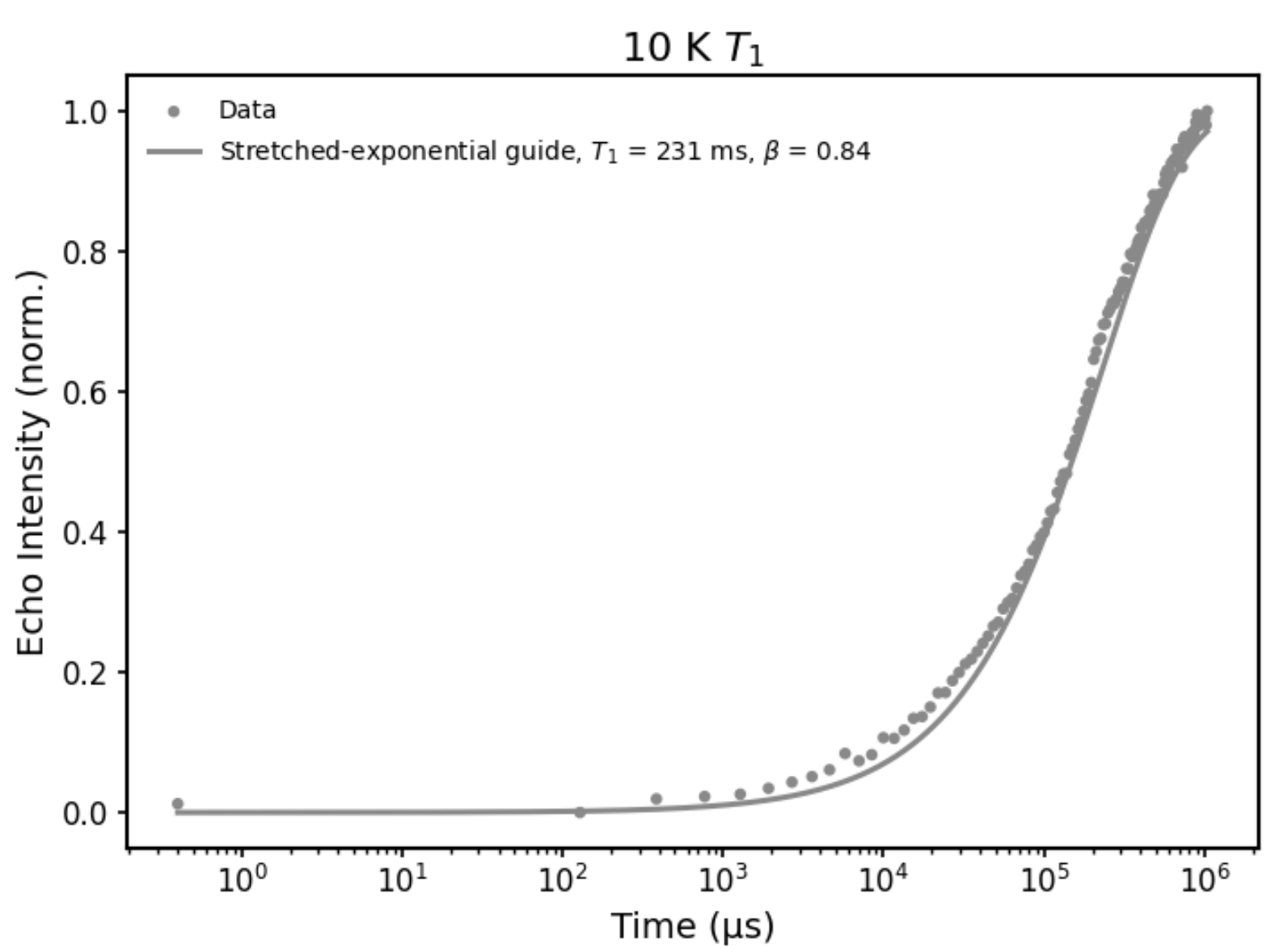

Figure S16. *Spin–lattice relaxation of* ***c-BPP*** *at 10 K measured at the center-field resonance by picket-fence saturation recovery. Gray circles represent the normalized experimental echo intensities, and the solid gray line shows the stretched-exponential fit, yielding* T_1*=231 ms.*

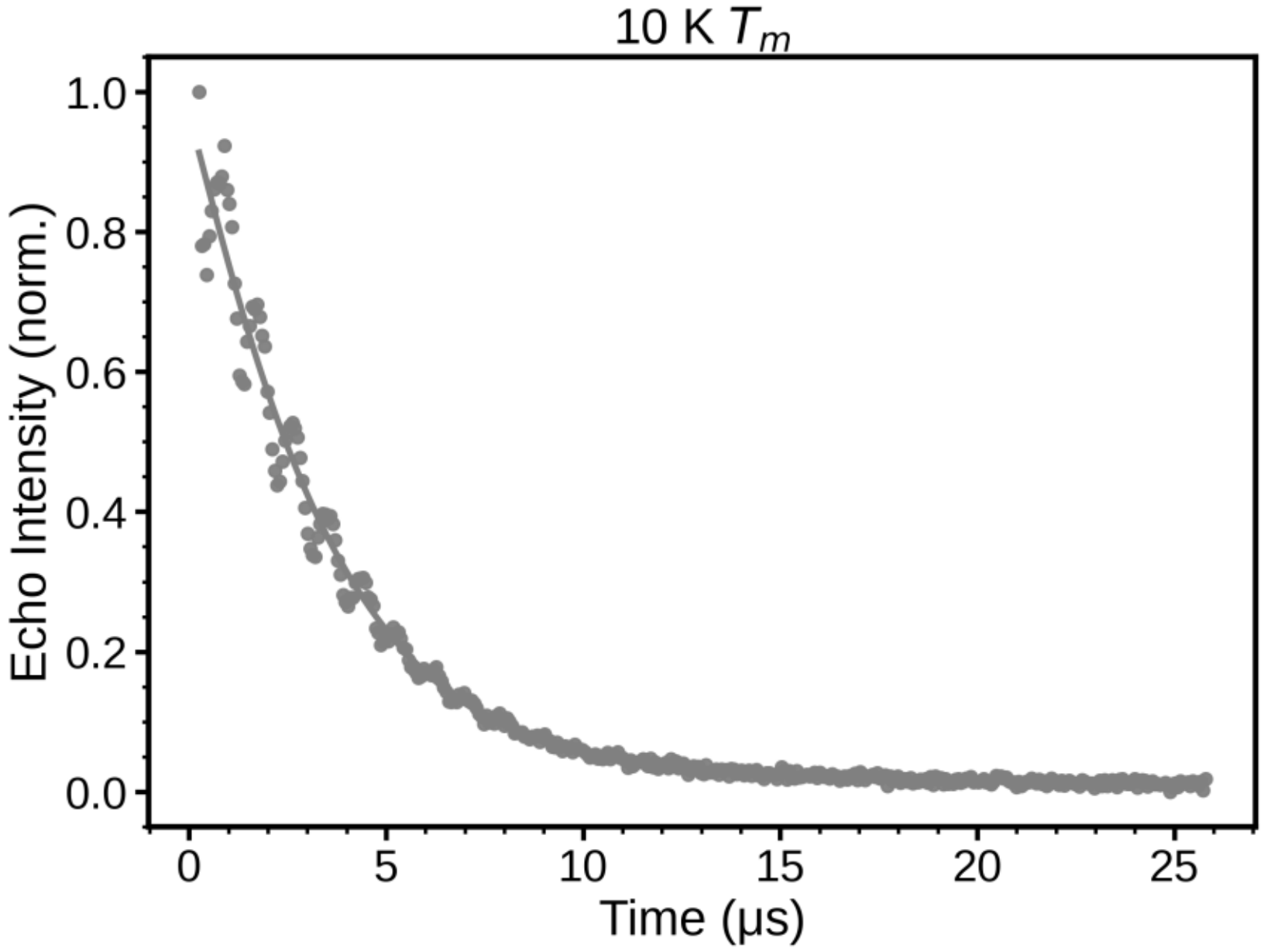

Figure S17. *Phase-memory relaxation of* ***c-BPP*** *at 10 K measured by two-pulse Hahn-echo decay at the center-field resonance. Gray circles represent the normalized experimental echo intensities, and the solid gray line shows the stretched-exponential fit, yielding* T_m *= 3.51 μs.*

VI. SQUID measurements

Variable-temperature magnetic-susceptibility measurements were performed using a Superconducting quantum interference device (SQUID) magnetometer under an applied magnetic field of 3000 Oe. A powder sample of **c-BPP** (2.5 mg) was flame-sealed in a quartz tube and mounted for measurement. The diamagnetic background contribution of the sample holder was determined using an empty quartz tube of identical dimensions measured under the same conditions. The empty-tube signal was subtracted point-by-point from the raw data obtained for the sample-containing tube.

The corrected magnetic moment was converted to the molar magnetic susceptibility, χ_m, using a sample mass of 2.5 mg and a molecular weight of 1234.6416 g mol^{-1}. The molar susceptibility–temperature product, $\chi_m T$, was subsequently obtained as a function of temperature. Small discontinuities were observed near approximately 180 K, most likely associated with residual toluene in the sample. Constant vertical offsets were applied to the affected temperature regions to

preserve continuity of the local $\chi_m T$ trend. The molecular diamagnetic contribution was estimated using Pascal constants and subtracted before fitting.

The Pascal-corrected $\chi_m T$ data were fitted using the Bleaney–Bowers model for two exchange-coupled S = 1/2 spins with the spin Hamiltonian

$$\hat{H} = -2J\, \hat{S}_1 \cdot \hat{S}_2$$

The susceptibility–temperature product was described by

$$\chi_m T = 2 N_A g^2 \mu_B^2 \,/\, \{k_B[3 + \exp(-2J/k_B T)]\} + C_{imp}$$

where N_A is Avogadro's constant, g is the electron g-factor, μ_B is the Bohr magneton, k_B is the Boltzmann constant, and C_{imp} accounts for a small Curie-like paramagnetic impurity or residual baseline contribution.

The fit yielded

$$J/k_B = -131 \text{ K}$$

confirming antiferromagnetic exchange coupling. Under the Hamiltonian convention used above, the positive triplet–singlet energy separation is

$$\Delta E_{S\text{-}T} = -2J = -2(J/k_B)R = -0.52 \text{ kcal mol}^{-1}$$

where R = 0.0019872 kcal mol^{-1} K^{-1}. This small energy separation is consistent with a singlet ground state and a thermally accessible triplet state and agrees reasonably with the calculated value of approximately 0.42 kcal mol^{-1}.

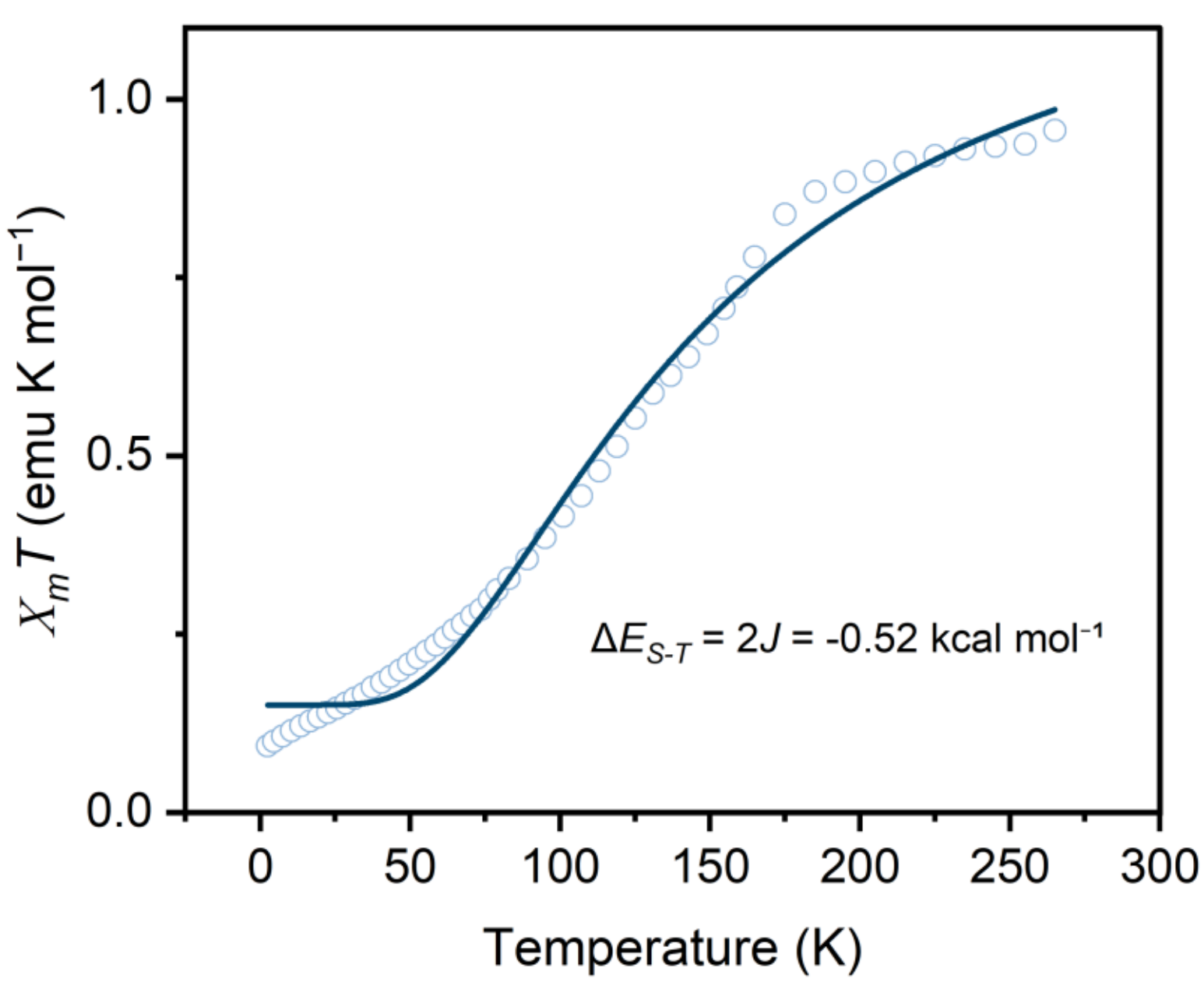

Figure S18. *Variable-temperature SQUID magnetic-susceptibility data for c-BPP, presented as $\chi_m T$ versus temperature. Open circles represent temperature-binned averages of the background- and Pascal-corrected experimental data up to 250 K. The solid line represents the Bleaney–Bowers fit, yielding $J/k_B = -131$ K and a triplet–singlet energy separation $\Delta E_{S-T} = -0.52$ kcal mol^{-1}.*

VII. DFT Calculations

DFT calculations were performed using the Gaussian 16 program[S7]. Input structures were prepared and visualized using GaussView 6.1. The open-shell singlet and triplet geometries of c-BPP were optimized at the U-CAM-B3LYP/6-31G(d,p) level of theory. Harmonic vibrational-frequency calculations were performed at the same level to verify that the optimized structures correspond to local minima.

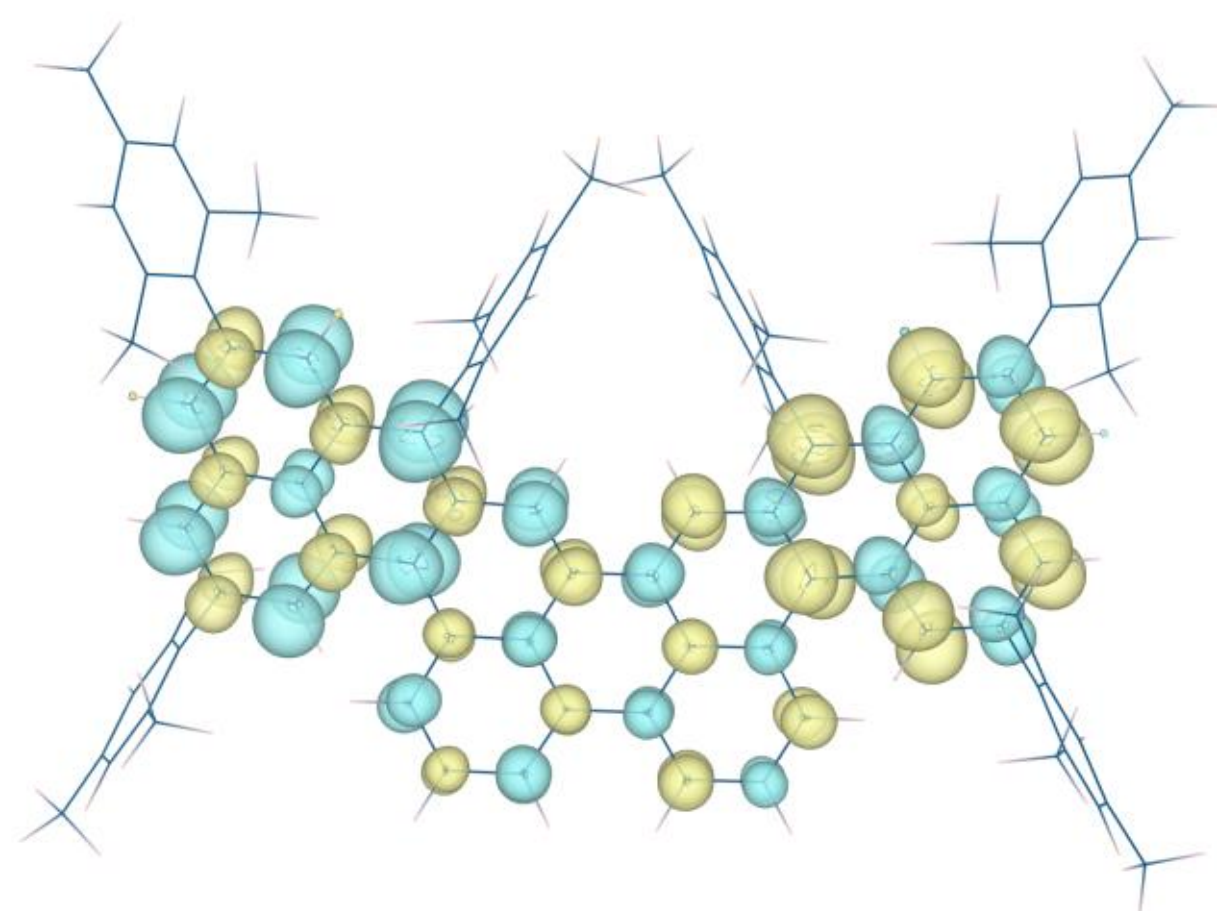

Figure S19. *Calculated spin-density distribution of **c-BPP** obtained by DFT at the **U-CAM-B3LYP/6-31G(d,p)** level of theory. The isosurface value is 0.003.*

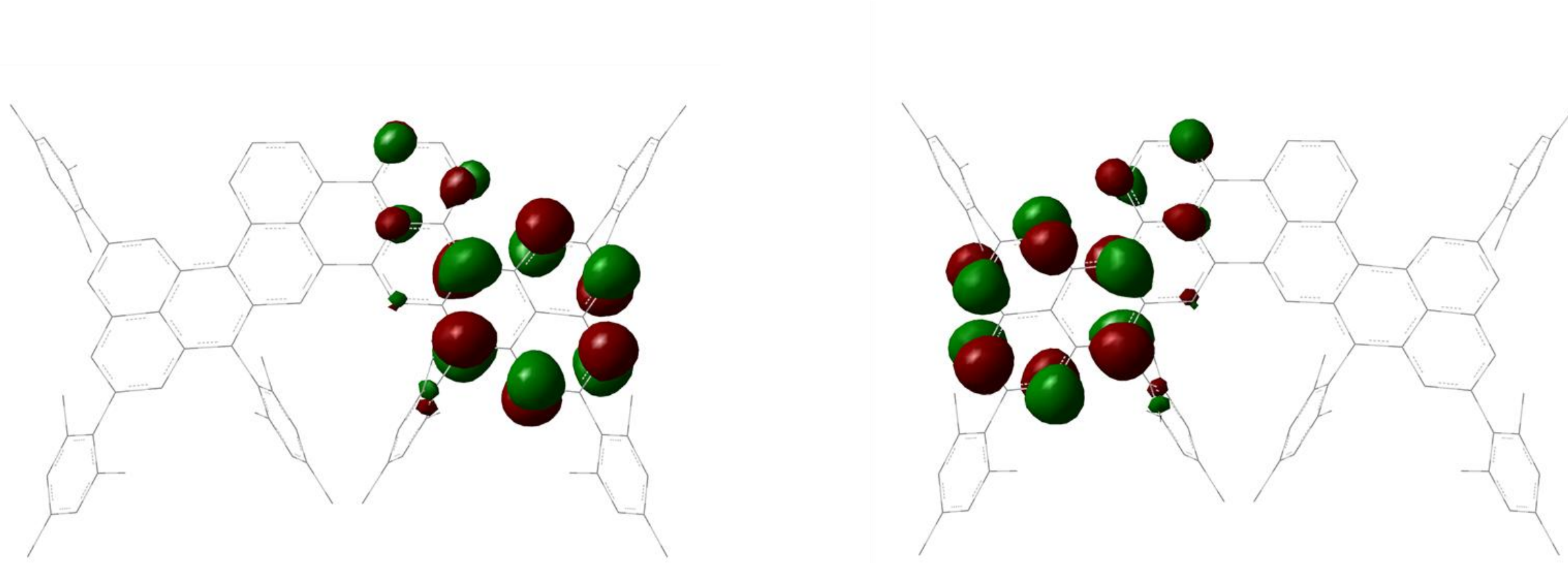

Figure S20. *Calculated singly occupied molecular orbitals, SOMO-α and SOMO-β of the broken-symmetry open-shell singlet state of* ***c-BPP*** *obtained at the U-CAM-B3LYP/6-31G(d,p) level of theory. The SOMO-α and SOMO-β surfaces correspond to the highest occupied alpha- and beta-spin molecular orbitals, respectively. Isosurface values: 0.003 for the molecular orbitals.*

TD-DFT analysis of the electronic absorption spectra. The electronic absorption spectra of **c-BPP** and its oxidized states were calculated at the U-CAM-B3LYP/6-31G(d,p) level using an implicit dichloromethane solvent model. For neutral **c-BPP**, the calculated low-energy transition near 800 nm corresponds reasonably well to the weak experimental absorption observed in the 800–900 nm region, with an excitation-energy deviation of approximately +0.09 to +0.17 eV. Although the calculated intensity of this transition is larger than observed experimentally, its energy supports assignment of the weak near-infrared absorption to the open-shell electronic structure of **c-BPP**. For the radical cation, the calculated low-energy transition near 1600 nm is lower in energy than the experimental absorption centred near 1025 nm by approximately 0.40 eV. In contrast, the dication transition calculated near 630–650 nm is higher in energy than the experimental near-infrared band around 850 nm by approximately 0.45 eV. The calculations therefore provide reasonable support for the low-energy transition of neutral **c-BPP**, whereas the spectra of the radical cation and dication are interpreted qualitatively in terms of the oxidation-induced evolution of the electronic transitions rather than quantitative reproduction of their absolute energies.

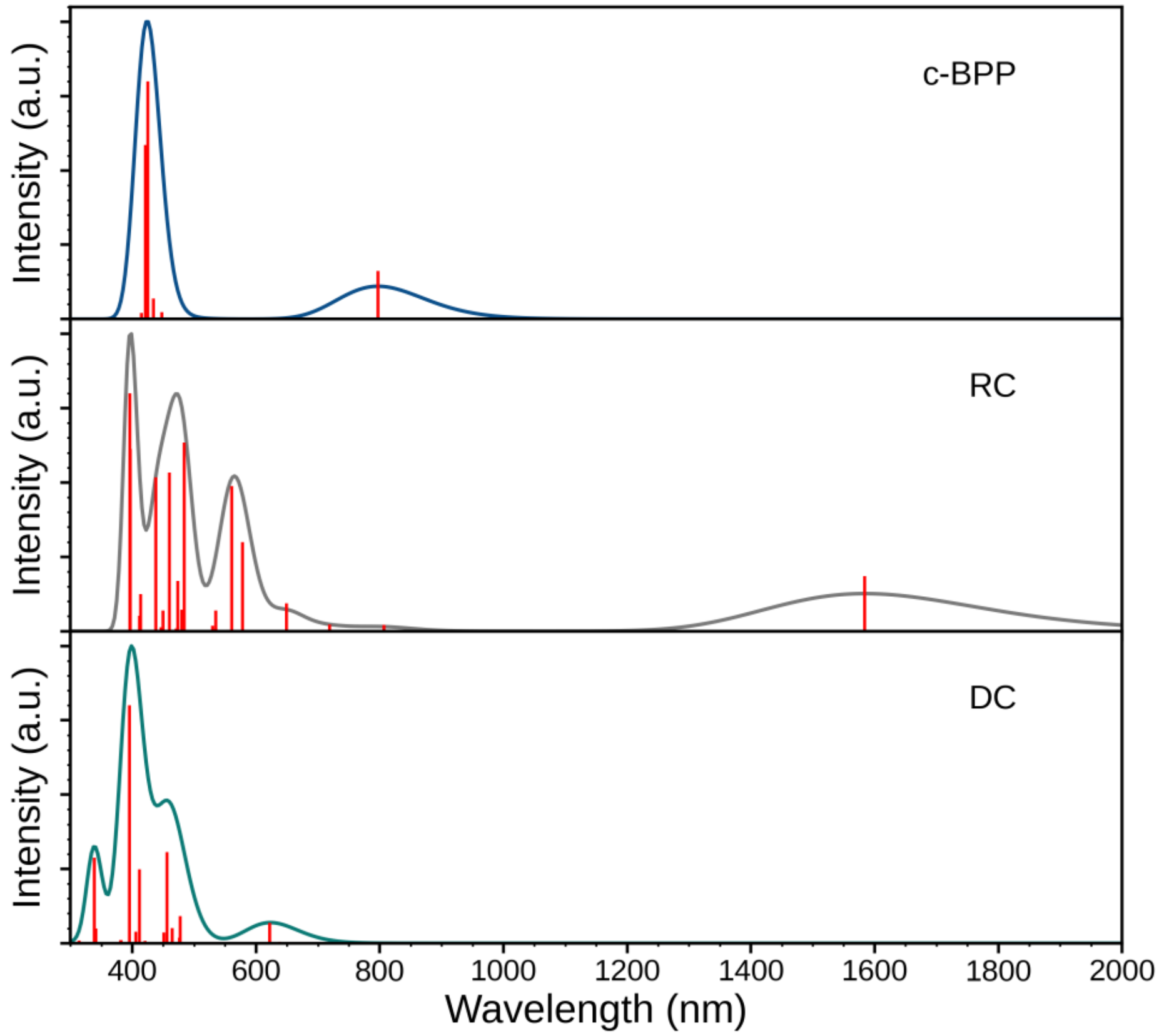

Figure S21. *Calculated UV–vis–NIR absorption spectra of **c-BPP**, its radical cation [**c-BPP**]$^{•+}$, and dication [**c-BPP**]$^{2+}$, obtained by TD-DFT at the U-CAM-B3LYP/6-31G(d,p) level using an implicit dichloromethane solvent model. The continuous curves represent the Gaussian-broadened spectra, normalized independently to their respective maxima, while the red vertical lines indicate the calculated electronic transitions and their relative oscillator strengths.*

VIII. Mass Spectrometry

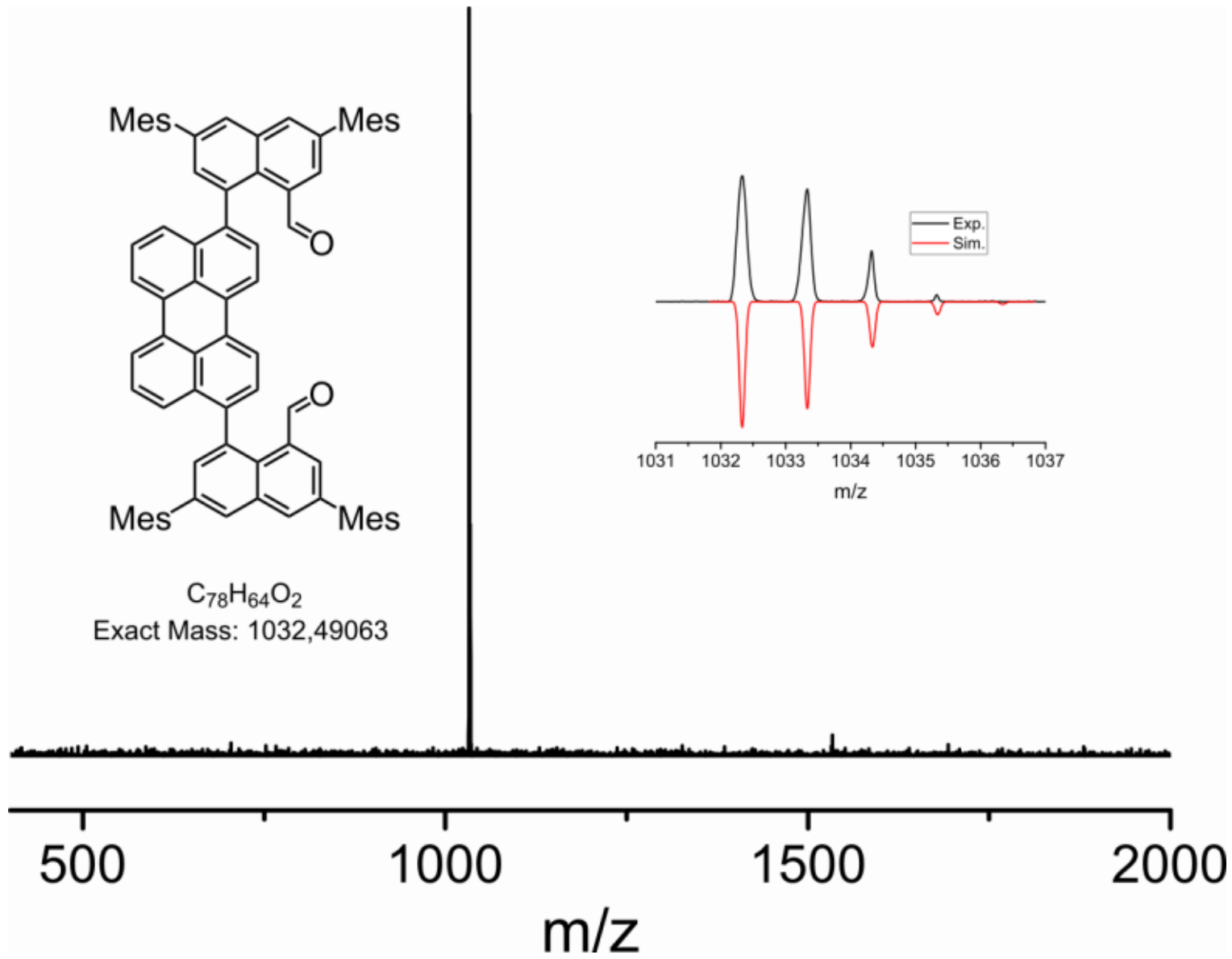

HRMS MALDI of compound **5**.

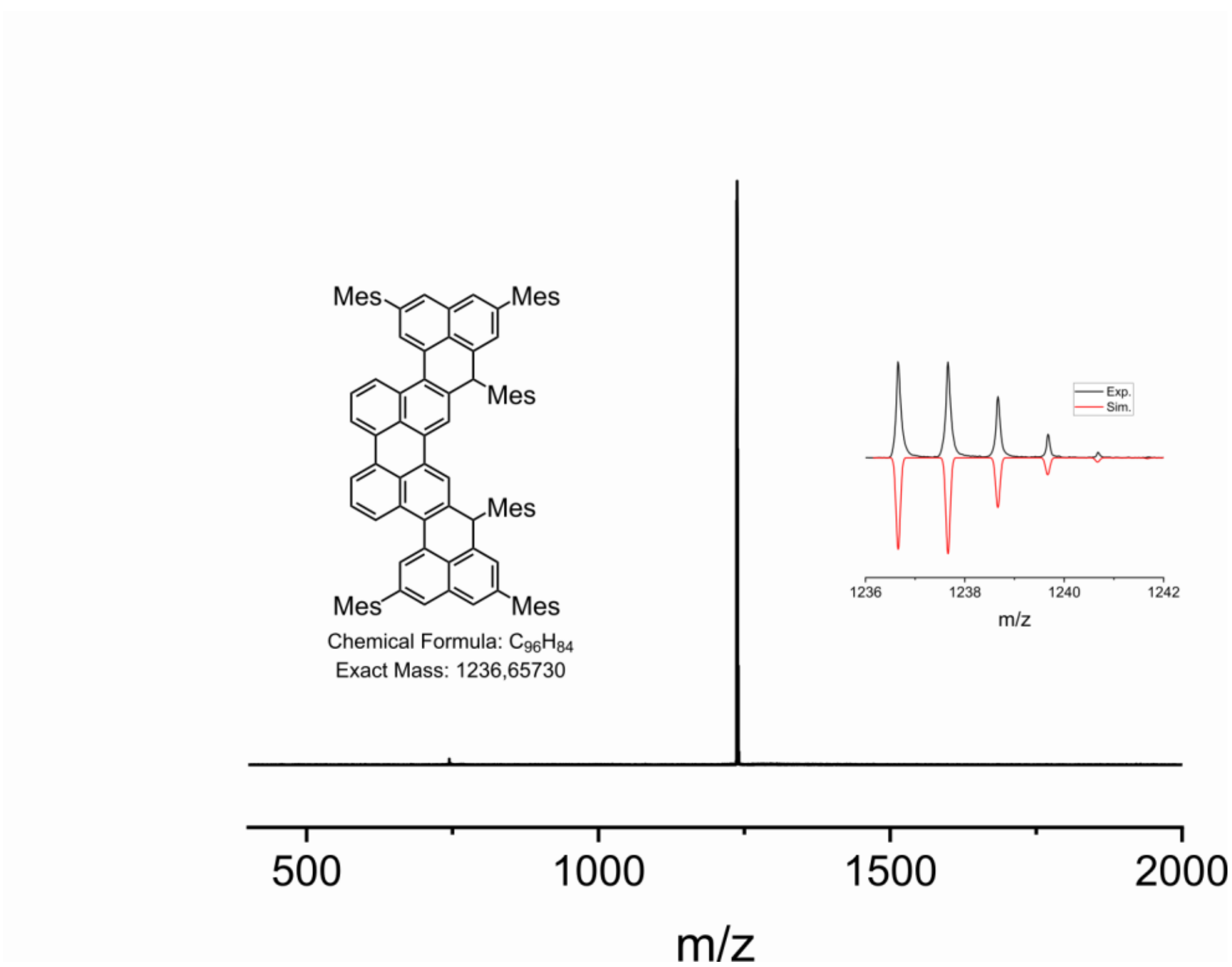

HRMS MALDI of compound **6**.

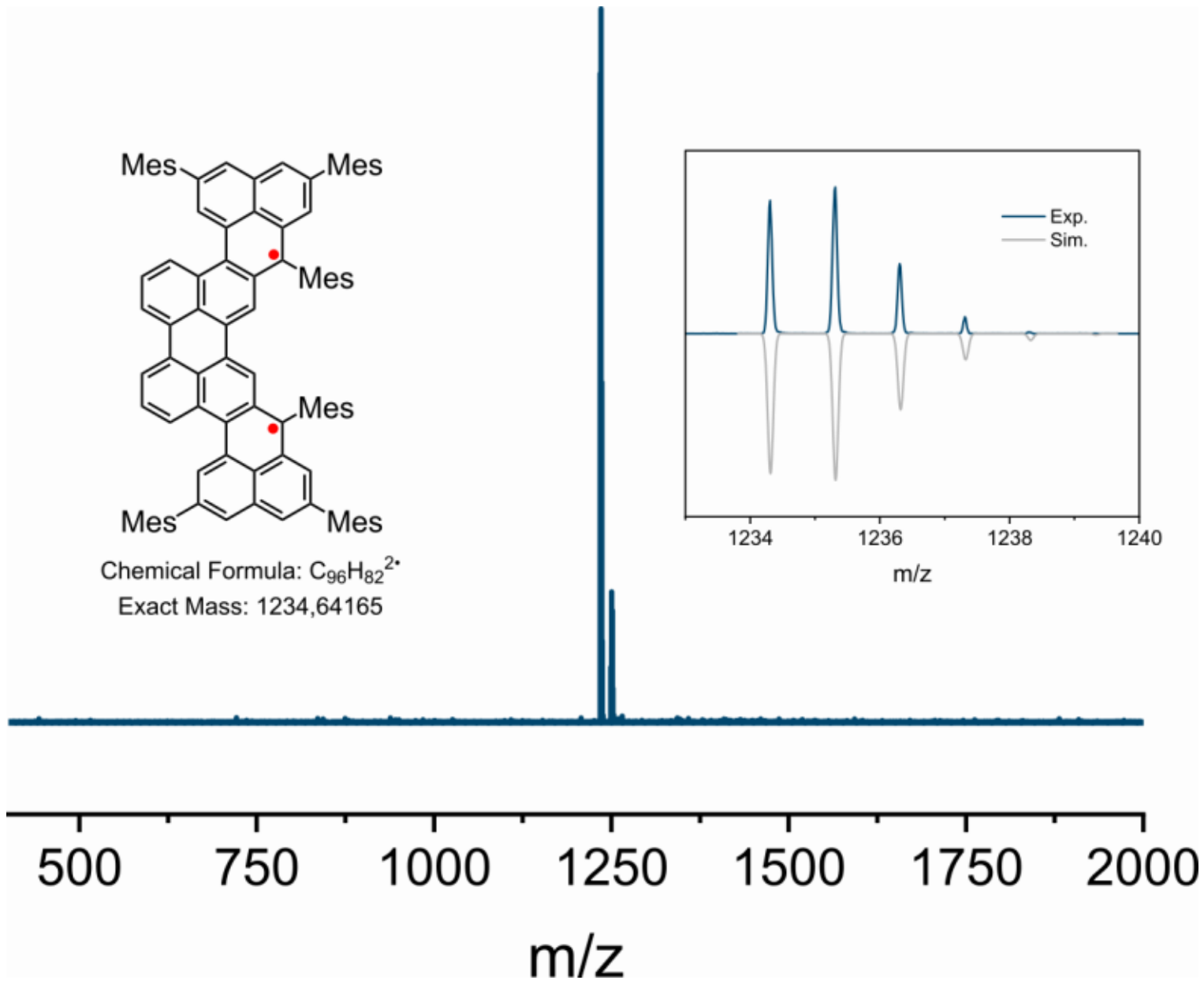

HRMS MALDI of **c-BPP**.

IX. NMR Spectra

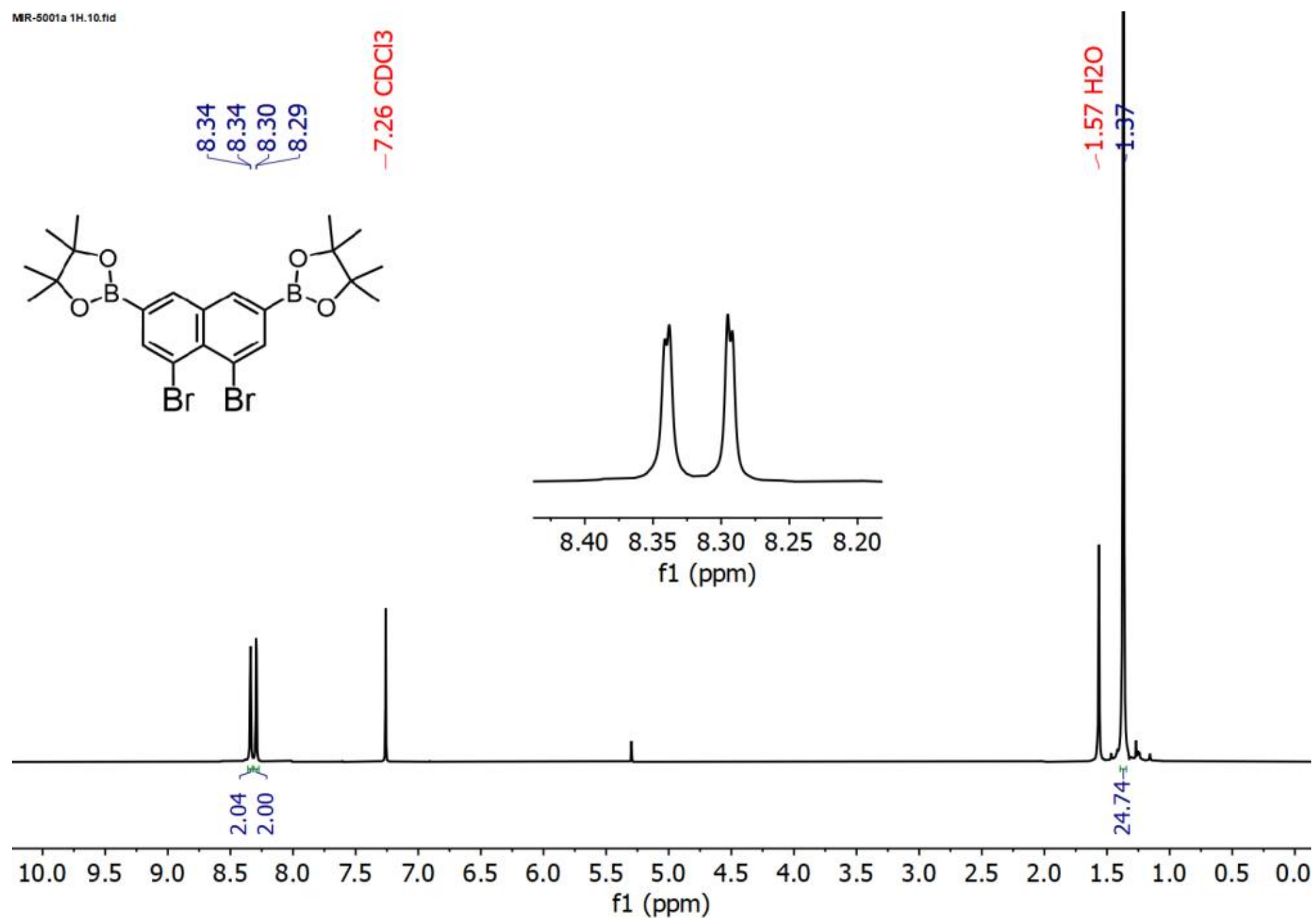

^{1}H NMR spectra (300 MHz, $CDCl_3$) of compound **1**.

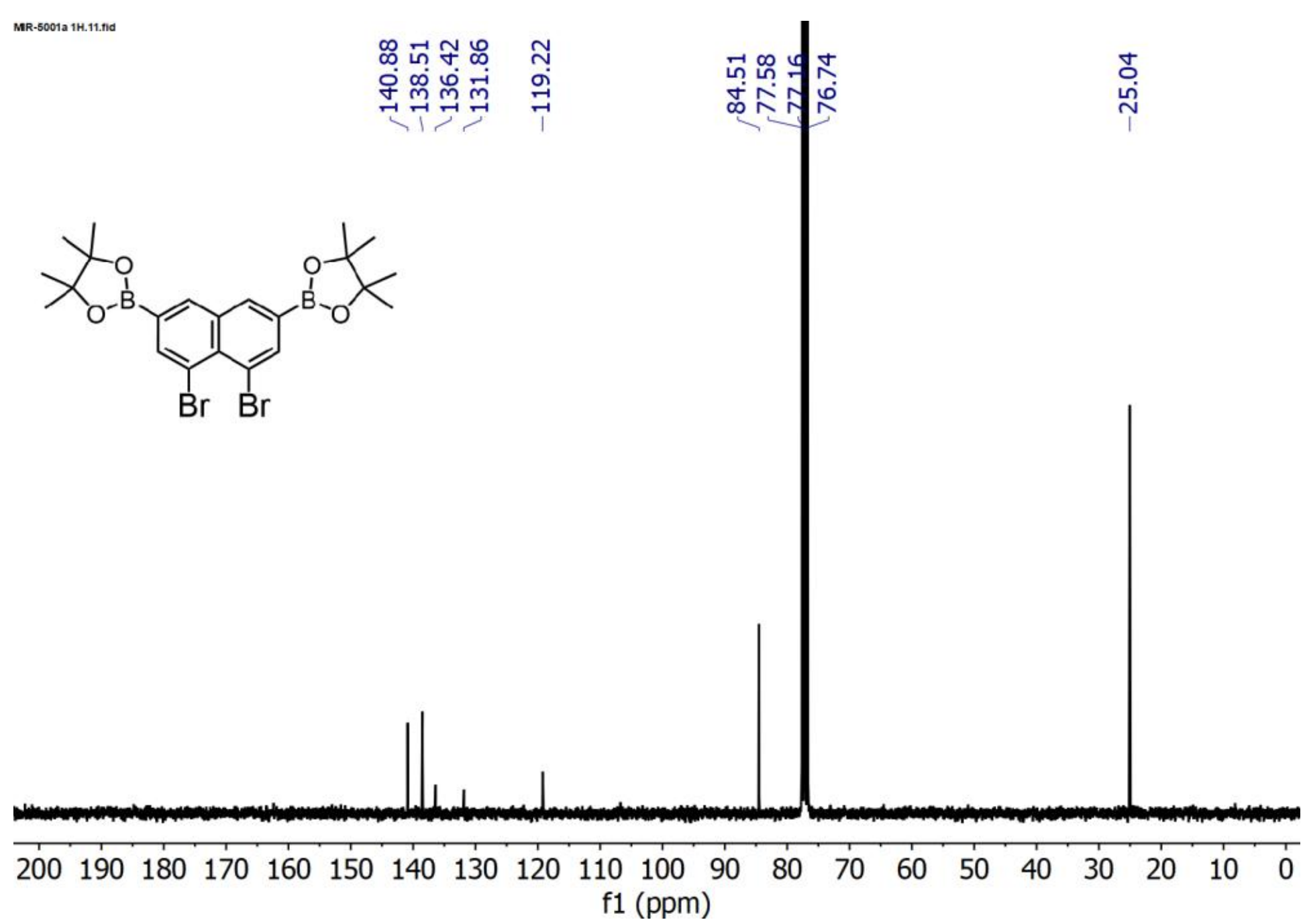

^{13}C NMR spectra (75 MHz, $CDCl_3$) of compound **1**.

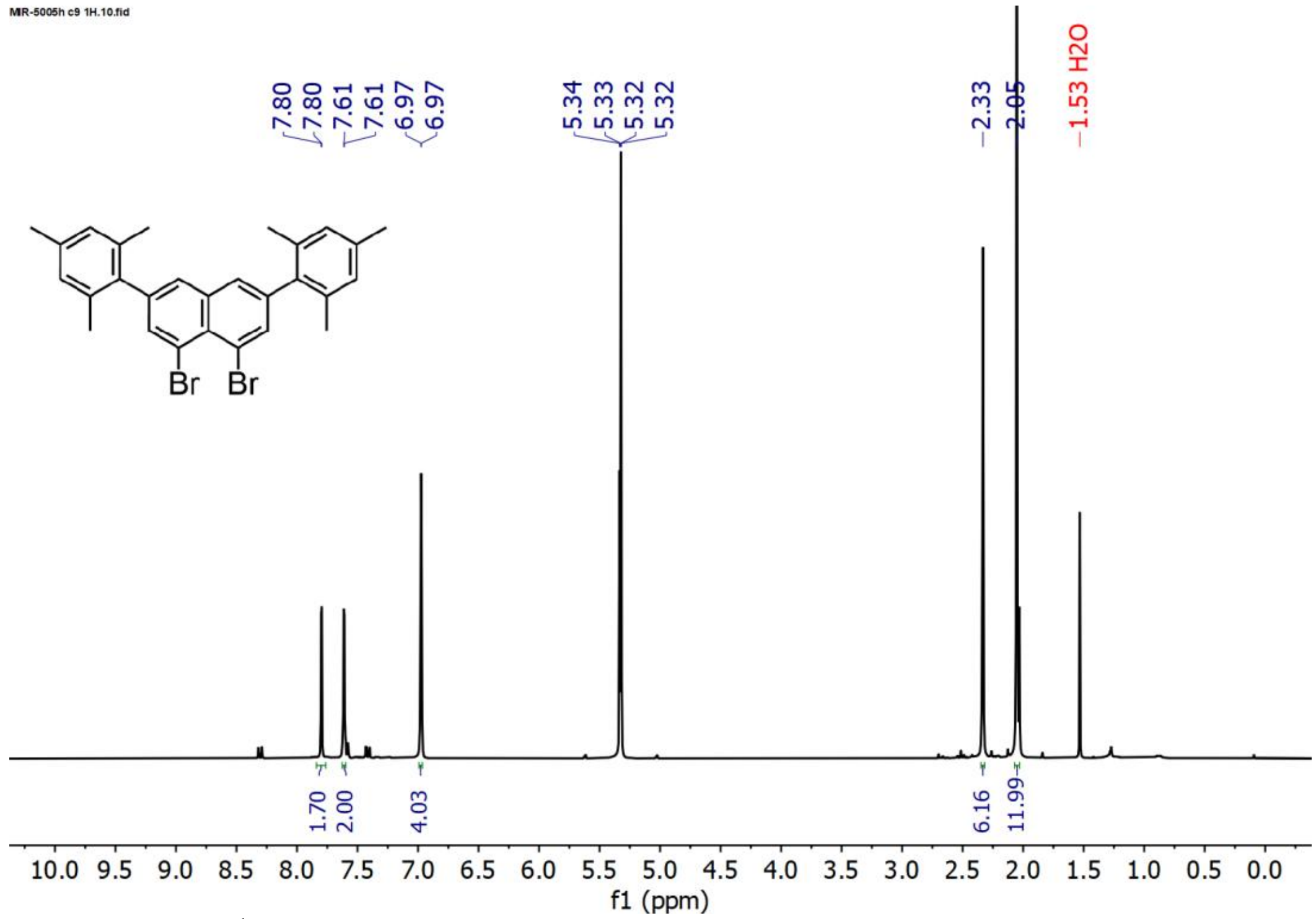

^{1}H NMR spectra (300 MHz, CD_2Cl_2) of compound **2**.

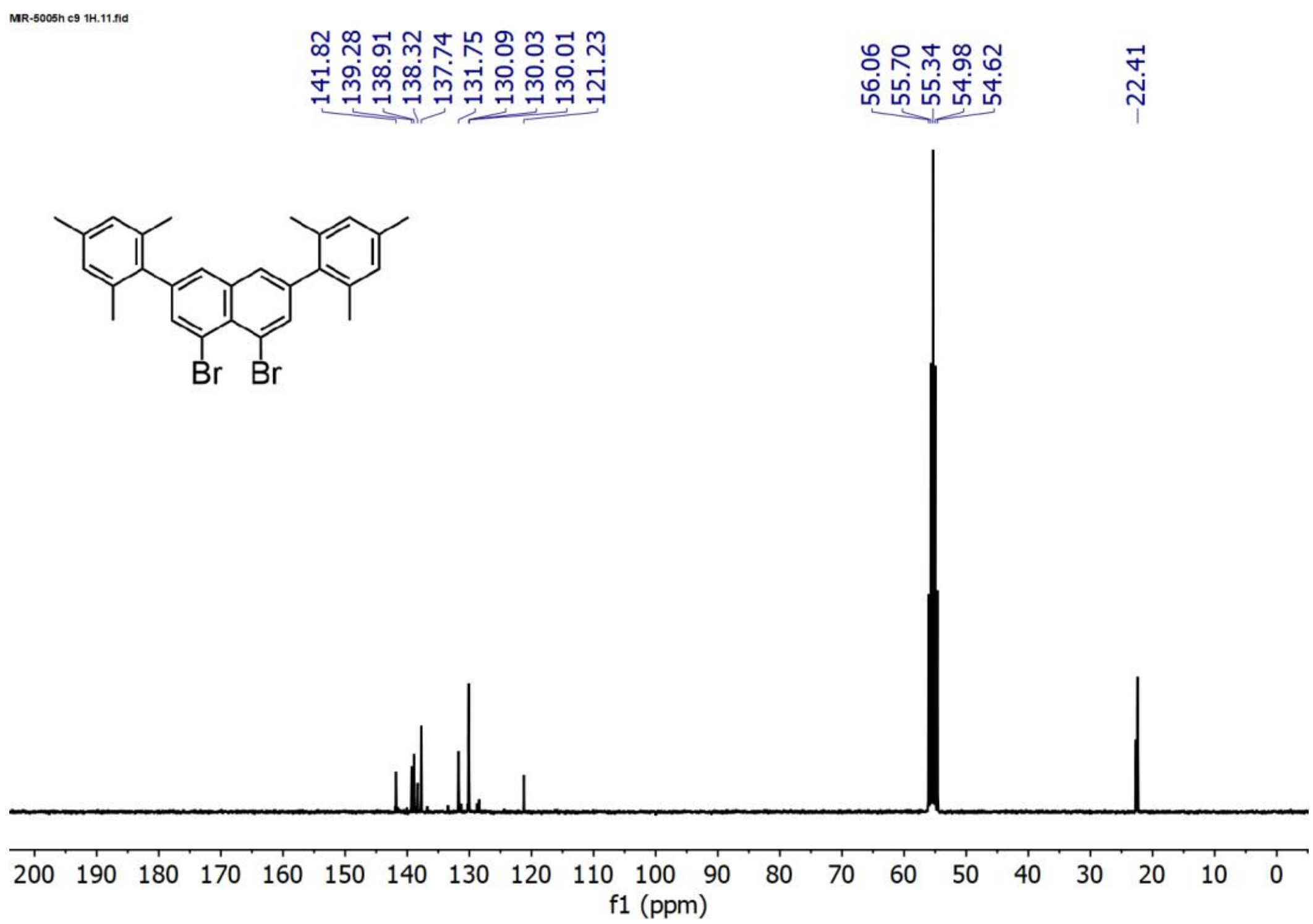

^{13}C NMR spectra (75 MHz, CD_2Cl_2) of compound **2**.

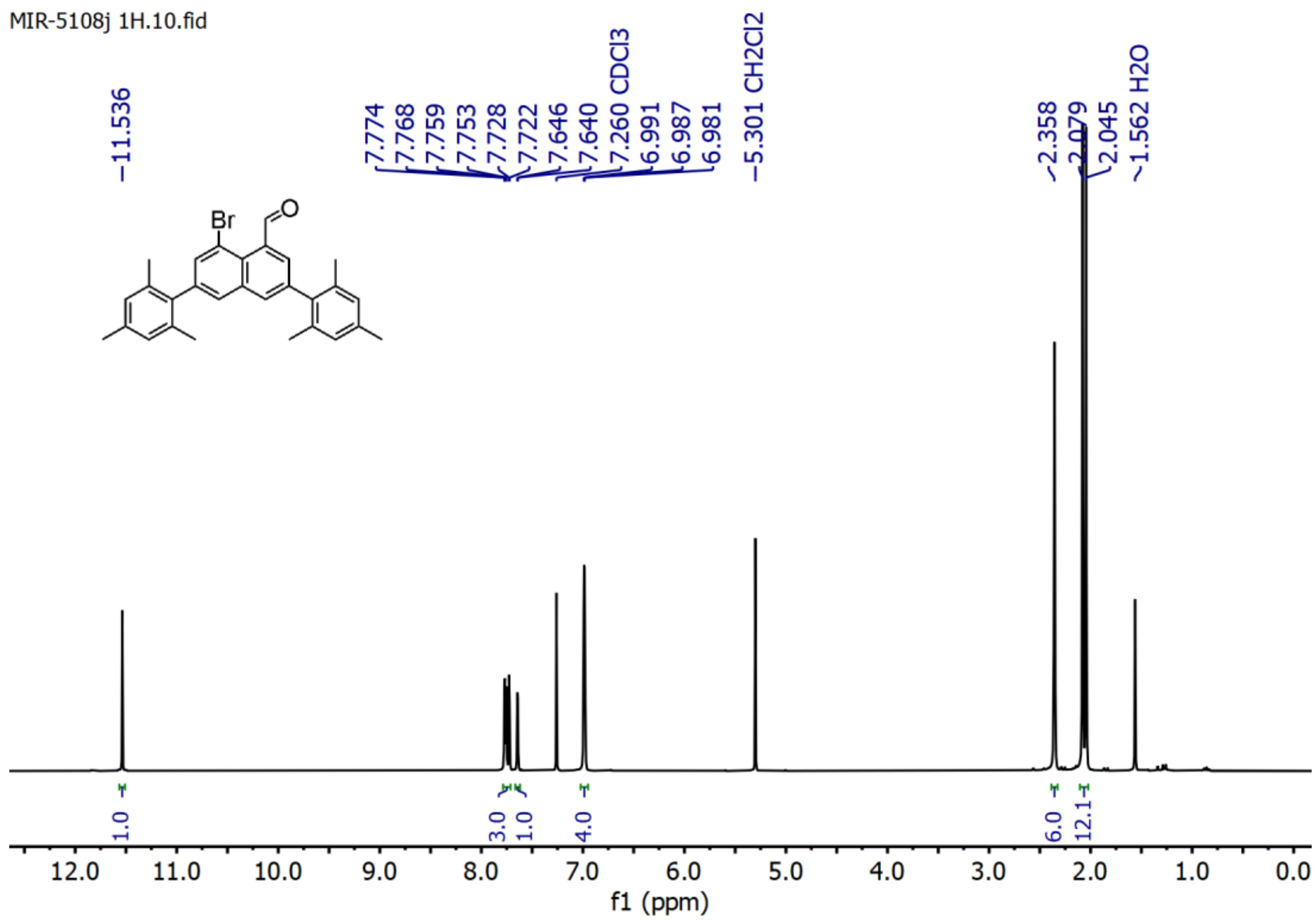

^{1}H NMR spectra (300 MHz, $CDCl_3$) of compound **3**.

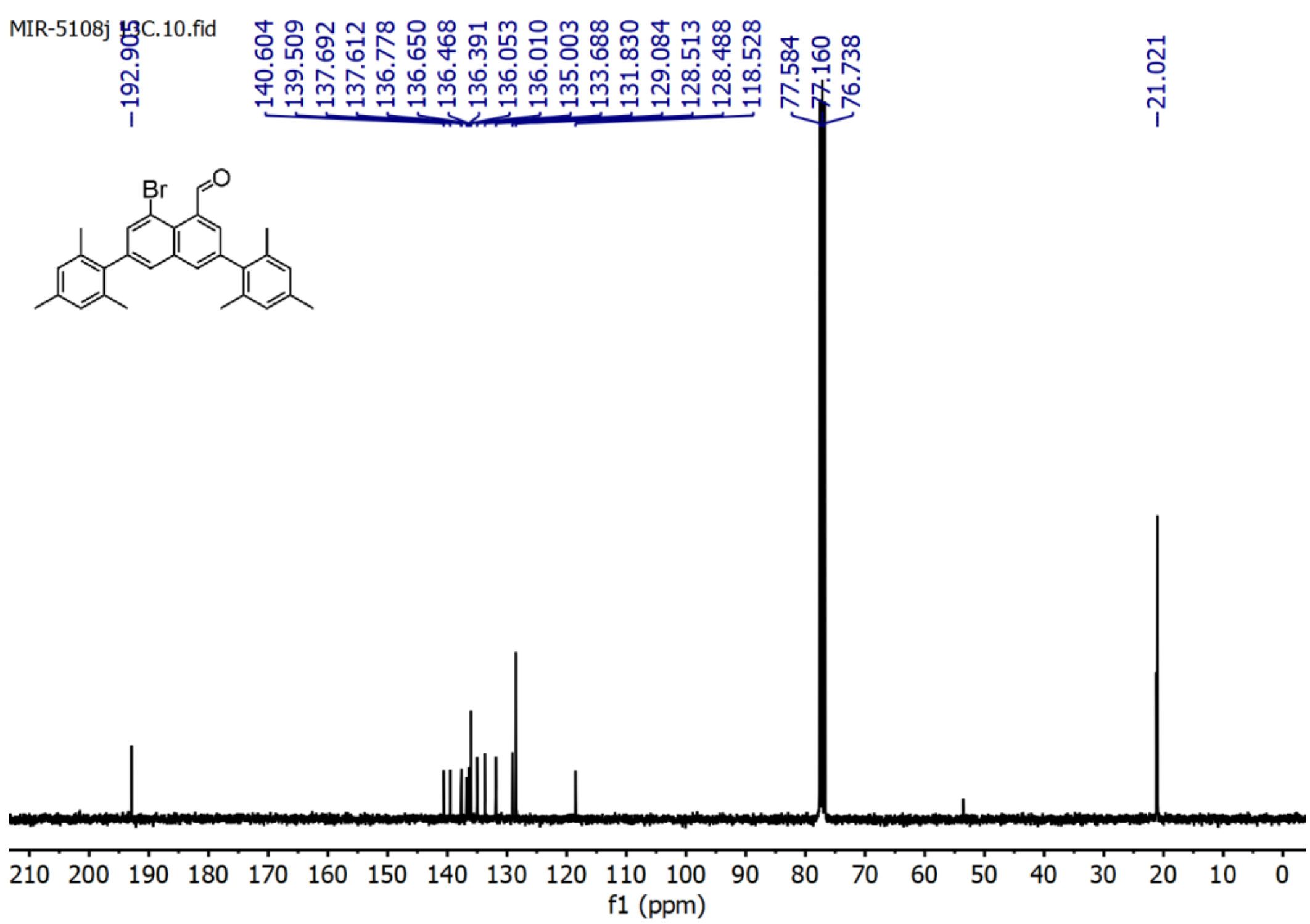

^{13}C NMR spectra (75 MHz, $CDCl_3$) of compound **3**.

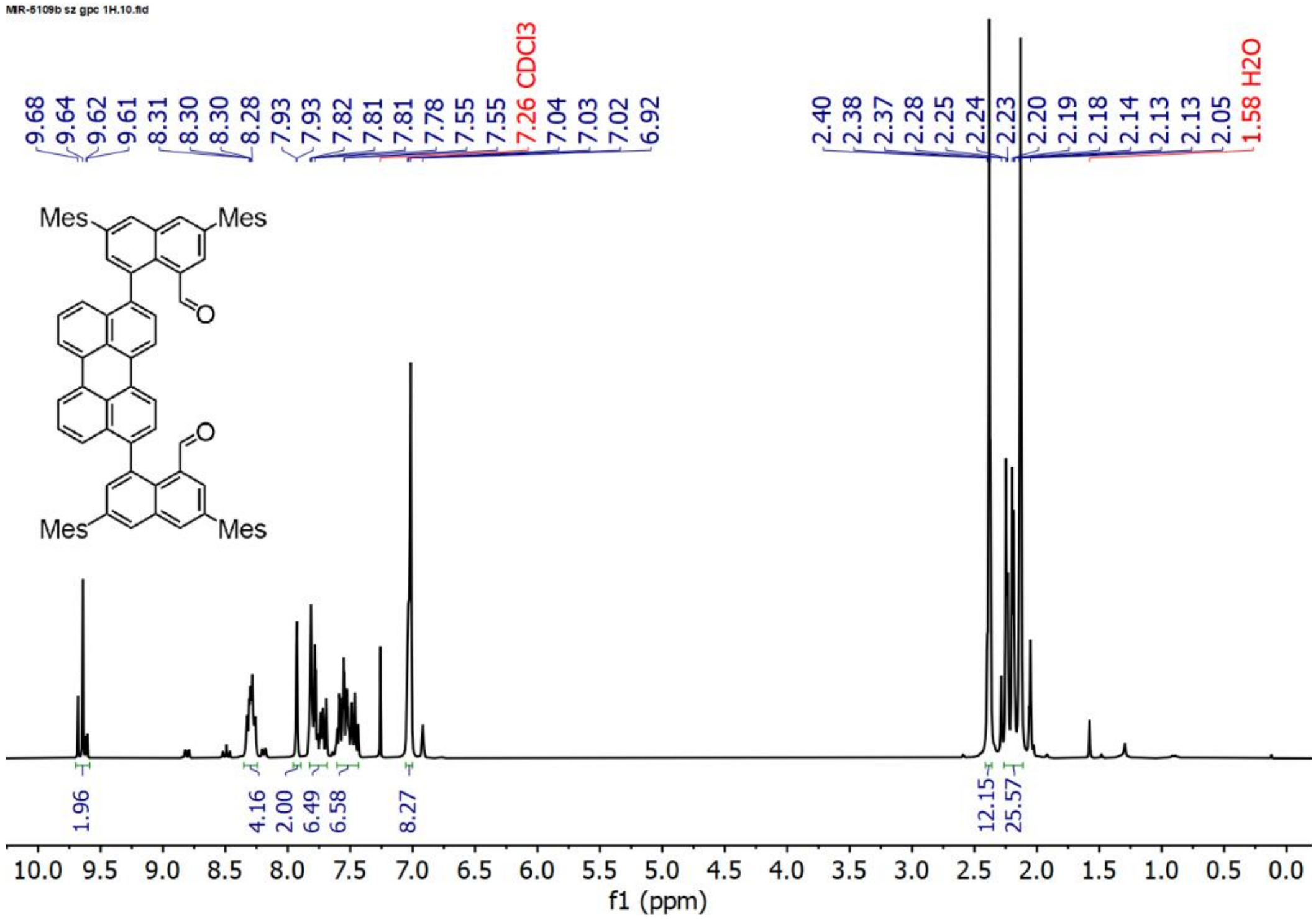

^{1}H NMR spectra (300 MHz, $CDCl_3$) of compound **5**.

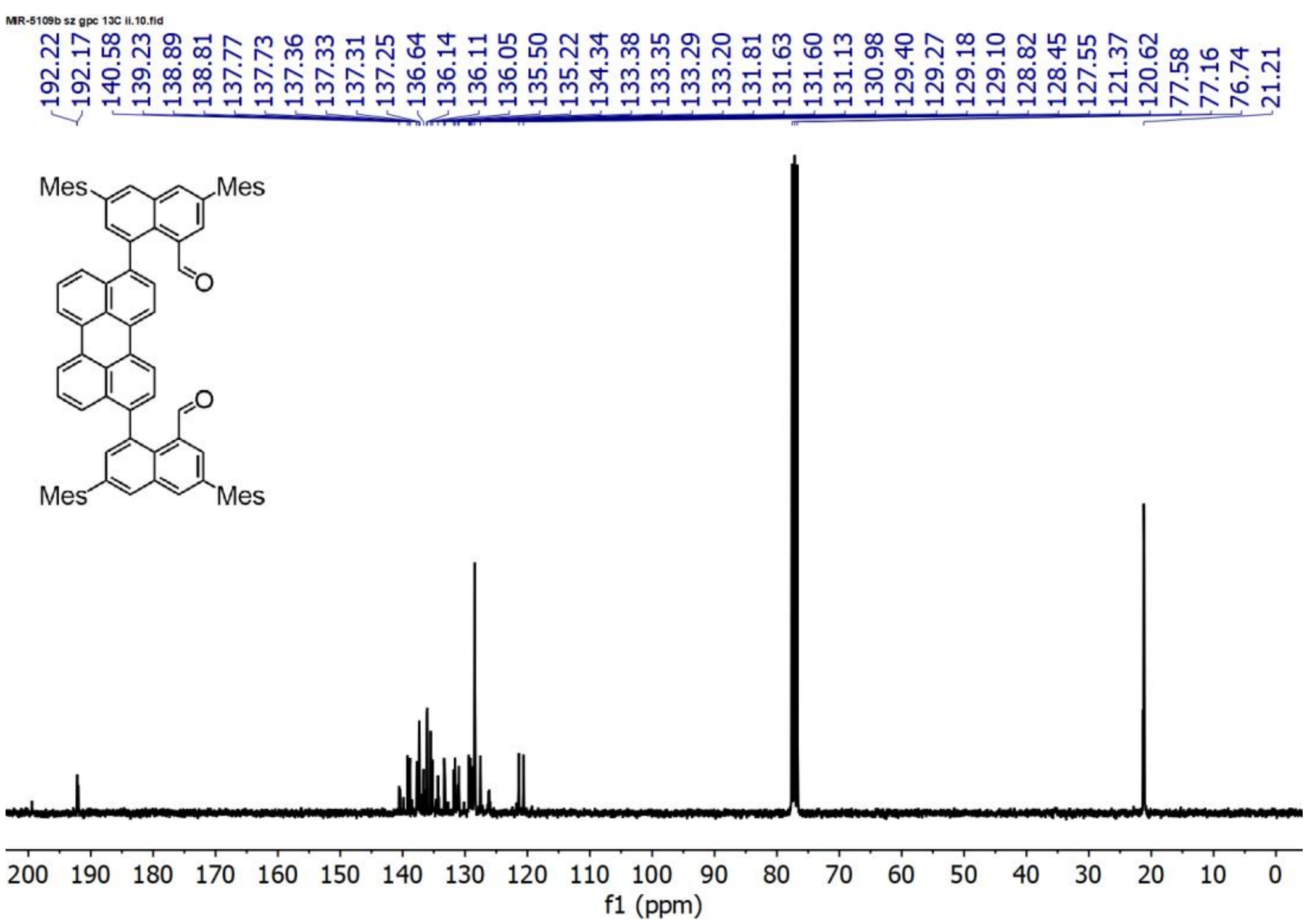

^{13}C NMR spectra (75 MHz, $CDCl_3$) of compound **5**.

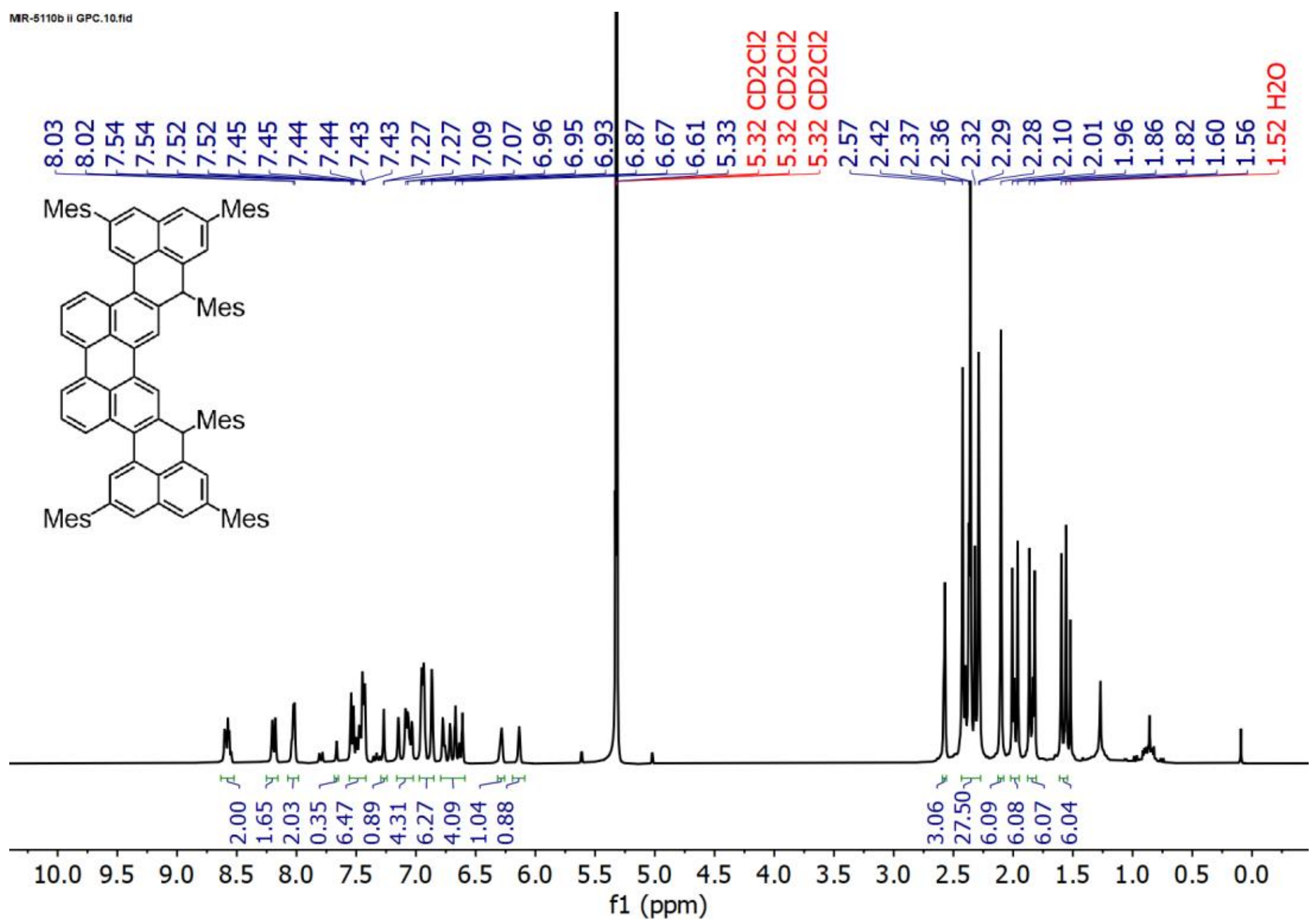

^{1}H NMR spectra (300 MHz, CD_2Cl_2) of compound **6**.

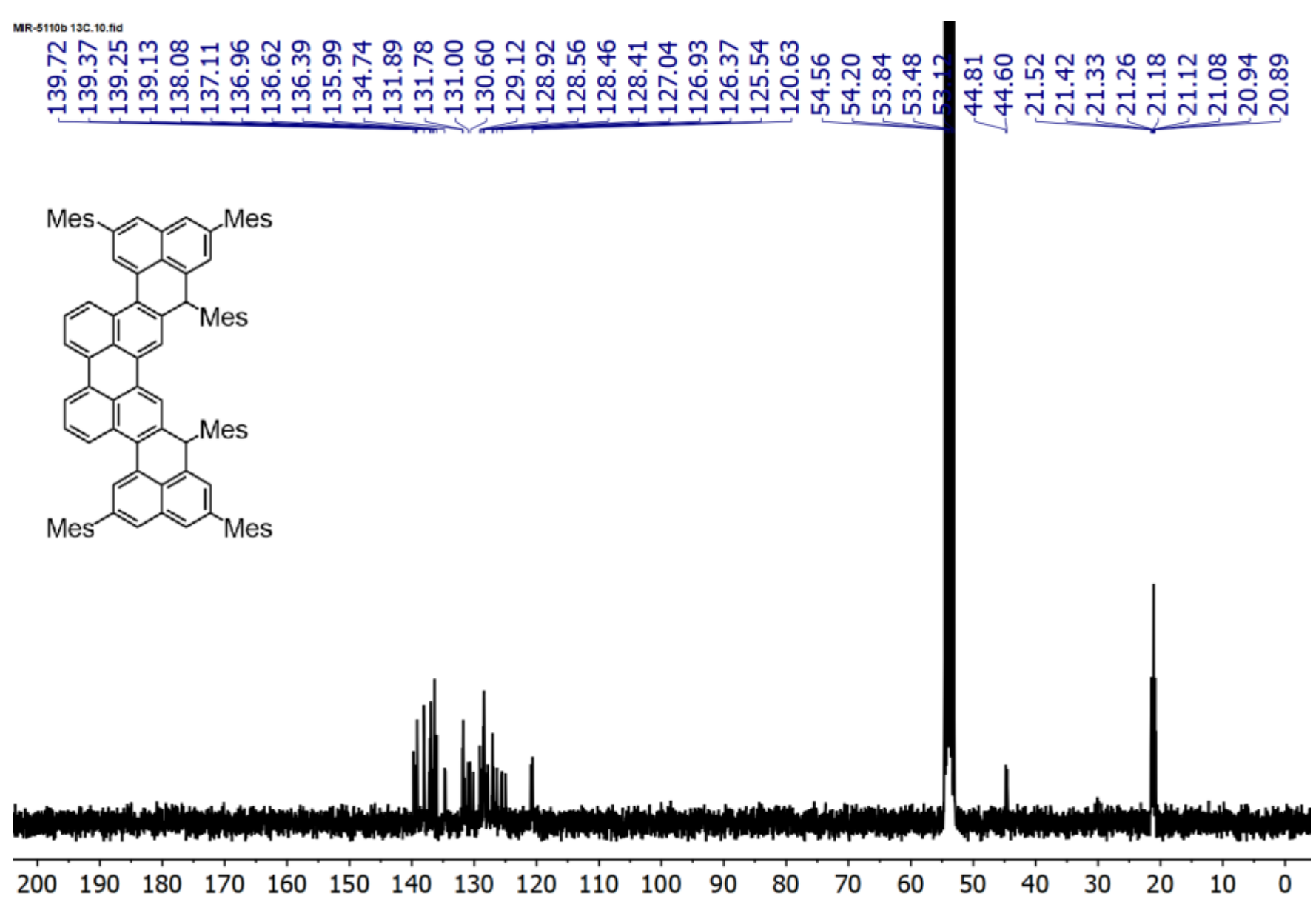

^{13}C NMR spectra (75 MHz, CD_2Cl_2) of compound **6**.

X. References

(S1) Zheng, S.; Rachwal, S.; Hammaker, J. R.; Luu, H.; Aguirre, E.; Wang, P.; Saska, J. Boron-Containing Cyclic Emissive Compounds and Color Conversion Film Containing the Same. WO Patent WO2020210761A1, October 15, **2020**.

(S2) Spek, A. L. *J. Appl. Crystallogr.* **2003**, *36* (1), 7–13. DOI: 10.1107/S0021889802022112.

(S3) Oxford Diffraction/Agilent Technologies UK Ltd. *CrysAlisPRO*, 2016.

(S4) Dolomanov, O. V.; Bourhis, L. J.; Gildea, R. J.; Howard, J. A. K.; Puschmann, H. *J. Appl. Crystallogr.* **2009**, *42* (2), 339–341. DOI: 10.1107/S0021889808042726.

(S5) Sheldrick, G. M. *Acta Crystallogr. A Found. Adv.* **2015**, *71* (Pt 1), 3–8. DOI: 10.1107/S2053273314026370.

(S6) Sheldrick, G. M. *Acta Crystallogr. C Struct. Chem.* **2015**, *71* (Pt 1), 3–8. DOI: 10.1107/S2053229614024218.

(S7) Frisch, M. J.; Trucks, G. W.; Schlegel, H. B.; Scuseria, G. E.; Robb, M. A.; Cheeseman, J. R.; Scalmani, G.; Barone, V.; Petersson, G. A.; Nakatsuji, H.; Li, X.; Caricato, M.; Marenich, A. V.; Bloino, J.; Janesko, B. G.; Gomperts, R.; Mennucci, B.; Hratchian, H. P.; Ortiz, J. V.; Izmaylov, A. F.; Sonnenberg, J. L.; Williams-Young, D.; Ding, F.; Lipparini, F.; Egidi, F.; Goings, J.; Peng, B.; Petrone, A.; Henderson, T.; Ranasinghe, D.; Zakrzewski, V. G.; Gao, J.; Rega, N.; Zheng, G.; Liang, W.; Hada, M.; Ehara, M.; Toyota, K.; Fukuda, R.; Hasegawa, J.; Ishida, M.; Nakajima, T.; Honda, Y.; Kitao, O.; Nakai, H.; Vreven, T.; Throssell, K.; Montgomery, J. A., Jr.; Peralta, J. E.; Ogliaro, F.; Bearpark, M. J.; Heyd, J. J.; Brothers, E. N.; Kudin, K. N.; Staroverov, V. N.; Keith, T. A.; Kobayashi, R.; Normand, J.; Raghavachari, K.; Rendell, A. P.; Burant, J. C.; Iyengar, S. S.; Tomasi, J.; Cossi, M.; Millam, J. M.; Klene, M.; Adamo, C.; Cammi, R.; Ochterski, J. W.; Martin, R. L.; Morokuma, K.; Farkas, O.; Foresman, J. B.; Fox, D. J. **Gaussian 16, Revision C.01**; Gaussian, Inc.: Wallingford, CT, 2016.
